# Toripalimab plus axitinib in patients with metastatic mucosal melanoma: 3-year survival update and biomarker analysis

**DOI:** 10.1136/jitc-2021-004036

**Published:** 2022-02-21

**Authors:** Siming Li, Xiaowen Wu, Xieqiao Yan, Li Zhou, Zhihong Chi, Lu Si, Chuanliang Cui, Bixia Tang, Lili Mao, Bin Lian, Xuan Wang, Xue Bai, Jie Dai, Yan Kong, Xiongwen Tang, Hui Feng, Sheng Yao, Keith T Flaherty, Jun Guo, Xinan Sheng

**Affiliations:** 1 Key Laboratory of Carcinogenesis and Translational Research (Ministry of Education/Beijing), Department of Genitourinary Oncology, Peking University Cancer Hospital and Institute, Beijing, China; 2 Key Laboratory of Carcinogenesis and Translational Research (Ministry of Education/Beijing), Department of Melanoma and Sarcoma, Peking University Cancer Hospital and Institute, Beijing, China; 3 Medical Department, Shanghai Junshi Biosciences Co., Ltd, Shanghai, China; 4 Medical Department, TopAlliance Biosciences, Inc, Rockville, Maryland, USA; 5 Cancer Center, Massachusetts General Hospital, Boston, Massachusetts, USA

**Keywords:** melanoma, immunotherapy, drug therapy, combination, biomarkers, tumor

## Abstract

**Background:**

Mucosal melanoma is an aggressive melanoma subtype with poor response to antiprogrammed cell death-1 (PD-1) monotherapy. Axitinib in combination with toripalimab, a humanized IgG4 mAb against PD-1, showed a promising response rate in patients with metastatic mucosal melanoma (MM) in a phase Ib study. Here, we report the updated overall survival (OS), duration of response (DoR), and biomarker analysis results.

**Methods:**

Patients with advanced MM received toripalimab 1 or 3 mg/kg intravenously every 2 weeks combined with axitinib 5 mg orally two times per day until disease progression or unacceptable toxicity. Tumor programmed cell death ligand-1 (PD-L1) expression, tumor mutational burden (TMB), and gene expression profile (GEP) by messenger RNA sequencing were evaluated for correlation with survival.

**Results:**

As of April 2, 2021, the median follow-up was 42.5 months. Among 29 chemotherapy-naïve patients with metastatic MM, the median OS was 20.7 months (95% CI 9.7 to 32.7 months); the median progression-free survival (PFS) was 7.5 months (95% CI 3.8 to 14.8 months); and the median DoR was 13.4 months (95% CI 5.5 to 20.6 months). The OS rates of 1, 2, and 3 years were 62.1%, 44.8%, and 31.0%, respectively. Biomarker analysis found that PD-L1 expression and TMB level were not associated with survival benefits. In contrast, a 12-GEP signature correlated with improved PFS (17.7 vs 5.7 months, p=0.0083) and OS (35.6 vs 17.6 months, p=0.039).

**Conclusions:**

The 3-year survival update confirmed the antitumor activity and long-term survival benefit of the toripalimab plus axitinib combination in patients with advanced MM. The 12-gene GEP is of value in predicting the outcomes of vascular endothelial growth factor receptor-tyrosine kinase inhibitor and PD-1 blockade combination therapy, but requires further validation.

**Trial registration numbers:**

NCT03086174.

## Introduction

Defined as a melanoma subtype originated from a mucous membrane, mucosal melanoma (MM) occurs most commonly in the oral and nasal cavities and gastrointestinal and genitourinary tracts with an occult but aggressive natural disease course and poor prognosis.^1^ MM represents a rare subtype of melanoma in the Caucasian population^2^ but constitutes the second most common subtype in the Asian population.^3^

MM is genetically distinct from cutaneous melanoma (CM)^4^ with higher incidences in KIT^5 6^ and NRAS mutations^7^ but a lower rate of BRAF V600 alterations.^8^ In general, MM harbors a much lower tumor mutational burden (TMB) than CM, as DNA mutations caused by chronic ultraviolet sun exposure are not a major disease mechanism for MM.^9^ Such distinctions at the molecular level may lead to different responses to standard treatment between these two melanoma subtypes. In the past decade, the emergence of immune checkpoint inhibitors (ICIs) represented by antiprogrammed cell death-1 (PD-1) antibody and anticytotoxic T-lymphocyte antigen-4 (CTLA-4) antibody brought enormous advances to the clinical management of CM but less so for MM. Data from clinical trials demonstrated that the overall response rates (ORRs) from PD-1 blockade in MM from both the Asian (0%–13.3%)^10 11^ and Caucasian population (23.3%)^12^ were much lower than that of CM (33.7%–43.7%).^13–15^ Even the dual inhibition of PD-1 and CTLA-4 pathways yielded limited improvement in response rates (37.1%–43.0%) for patients with MM in the Caucasian population, with a median progression-free survival (PFS) of only 5.8–5.9 months.^16 17^

Recently, a prospective phase II trial from China randomly assigned 114 patients with metastatic MM to a paclitaxel and carboplatin treatment with or without bevacizumab in the first-line setting. Chemotherapy combined with bevacizumab in this trial significantly prolonged both the PFS (4.8 vs 3.0 months) and overall survival (OS) (13.6 vs 9.0 months) when compared with chemotherapy alone.^18^ The efficacy of the bevacizumab-containing regimen emphasizes the importance of incorporating an antivascular endothelial growth factor (VEGF) therapy in the therapeutic paradigm for patients with MM.^4^ In vivo studies have also shown that angiogenesis inhibition, specifically simultaneous inhibition of the vascular endothelial growth factor receptor (VEGFR) and PD-1 pathways in a mouse colon cancer model, increased T-cell infiltration and suppressed tumor growth synergistically.^19^

We conducted a phase Ib combination study of axitinib, a VEGFR tyrosine kinase inhibitor, with toripalimab, a humanized immunoglobulin G4 monoclonal antibody against PD-1, to treat patients with metastatic MM (ClinicalTrials.gov).^20^ This was the first study testing the combination of immunotherapy and VEGFR-targeting therapy in treatment-naïve patients with advanced MM. In the first analysis as of December 19, 2018, the combination demonstrated a manageable safety profile and showed promising antitumor activity (ORR 48.3%, median PFS 7.5 months).^20^ Based on the results, the combination of toripalimab plus axitinib for the treatment of MM was granted the orphan-drug and fast-track designation by the US Food and Drug Administration (FDA) for the first-line treatment of MM. A global phase III trial of toripalimab in combination with axitinib versus pembrolizumab for the first-line treatment of patients with advanced MM is planned. Nevertheless, the median OS and the median duration of response (mDoR) was not mature by the cut-off date in the first report.^20^ Here we report the 3-year survival data and updated biomarker analyses.

## Methods

### Patients and study design

This was a phase Ib, single-center, open-label, two-part (part A dose escalation, and part B cohort expansion) clinical trial (ClinicalTrials.gov). Eligible patients with metastatic melanoma (part A) or pathologically confirmed metastatic MM (part B) with at least one measurable lesion per Response Evaluation Criteria in Solid Tumors (RECIST) V.1.1 at baseline, Eastern Cooperative Oncology Group performance status of 0 or 1 and adequate organ and bone marrow function were enrolled. Exclusion criteria included history of autoimmune diseases, ongoing infections, or prior PD-1 checkpoint inhibitor therapy.

### Treatment and end points

Details regarding the trial designs in parts A and B were provided in the original publication.^20^ Axitinib (5 mg) was given orally two times per day and toripalimab was intravenously infused at 1 or 3 mg/kg every 2 weeks (online supplemental figure 1) until disease progression or unacceptable toxicity. Responses were evaluated by investigators using both RECIST V.1.1 and immune-related RECIST (irRECIST). Patients who initially developed progressive disease (PD) per RECIST V.1.1 were allowed to continue therapy if the investigator considered patients to be benefiting from the treatment per irRECIST. The primary endpoint was safety, tolerability, and evaluation of dose-limiting toxicity of the combination treatment. The secondary endpoints included the pharmacokinetic profile and immunogenicity of toripalimab in the combination study, antitumor activity (ORR, disease control rate, DoR, PFS, and OS), and the status of antiprogrammed cell death ligand-1 (PD-L1) and other biomarkers as well as their correlations with clinical efficacy.

10.1136/jitc-2021-004036.supp1 Supplementary data

10.1136/jitc-2021-004036.supp2 Supplementary data

### PD-L1 expression analysis by immunohistochemistry (IHC)

Fresh or archival tumor biopsy samples were obtained from each patient before treatment initiation. PD-L1 expression was assessed by IHC staining using an anti-PD-L1 antibody (clone SP263, Ventana) on a Ventana (Tucson, Arizona, USA) autostainer by certified pathologists.^21^ PD-L1 positive expression was defined as the presence of membrane staining of any intensity in ≥1% of tumor cells or the presence of PD-L1 staining of any intensity in tumor-infiltrating immune cells covering ≥1% of tumor area occupied by tumor cells, associated intratumoral cells, and contiguous peritumoral stroma.

### Whole-exome sequencing (WES) and TMB analysis

WES was performed using the Sure-Select Human All Exon V6 kit (Agilent, Santa Clara, California, USA) on tumor tissue sections and matched peripheral blood samples. Genomic alterations were assessed, which included microsatellite stability status, single-nucleotide variants, insertions/deletions (indels), copy number variants, and gene rearrangement and fusions.

The TMB was determined by analyzing somatic mutations, including coding base substitution and indels per million base pairs. The TMB^High^ group was defined as TMB of ≥6 mutations per million base pairs (Mbp), according to the original publication.^20^

### Messenger RNA (mRNA) expression profile analysis

Tumor biopsy tissues were used to isolate mRNA, followed by complementary DNA synthesis, then sequencing on the NovaSEquation 5000/6000 platform (Illumina, San Diego, California, USA). The relative abundance of each annotated transcript was recorded as transcripts per million and log_2_ transformed before analysis. Expression panels included inflammation signature (IL-6, CXCL1, CXCL2, CXCL3, CXCL8, and PTGS2),^22^ angiogenesis signature (VEGFA, KDR, ESM1, PECAM1, ANGPTL4, and CD34),^22^ and interferon gamma (IFN-γ) signature (IDO1, CXCL10, CXCL9, HLA-DRA, STAT1, and IFNG).^23^ A 12-gene expression signatures of eight immune-related genes (CD274/PD-L1, CXCR6, CD27, CXCL9, IDO1, TIGIT, PDCD1LG2/PD-L2, and LAG3) and four angiogenesis-related genes (ANGPTL5, ANGPTL6, CD34, and KDR) were derived from panels^22 23^ with known association clinical benefits and were selected based on the best differential fit (responder vs non-responder). The abundance of RNA transcripts of selected genes was loaded into the logistic regression model to best fit coefficients to achieve the best receiver operating characteristic performance. The mean expression of the genes composing the signature was calculated to obtain a gene expression profile (GEP) score for the expression signature of each sample. The GEP cut-off of 450 was chosen so that the ORRs were 100% in the GEP high group and 0% in the GEP low group.

### Statistical analysis

Safety and efficacy analyses included all patients who received at least one dose of the study treatment. The ORR and its 95% exact CI were determined by the Clopper and Pearson method. PFS and OS were plotted using the Kaplan-Meier method, with medians and corresponding two-sided 95% CIs reported. A p value of <0.05 was considered statistically significant. Statistics analyses were performed using SAS V.9.4 or GraphPad Prism software (GraphPad Software, San Diego, California, USA).

## Results

### Patient population

A total of 33 patients with advanced melanoma were enrolled in the study from April 25, 2017, to April 2, 2018 (online supplemental figure 2 and table 1). The majority of patients (31 of 33 patients) were naïve to systemic chemotherapy. Among 31 treatment-naïve melanomas, 2 were of unknown primary and 29 were pathologically confirmed MMs. By the cut-off date of April 2, 2021, 3 patients remained on the study treatment, 1 patient discontinued treatment due to an adverse event (AE), and 29 patients discontinued treatment due to PD. No new treatment-related AEs emerged during the 28 months since the previous report by the cut-off date of December 19, 2018. Incidences of permanent discontinuation due to AE and the use of corticosteroids remained unchanged from the previous report.

10.1136/jitc-2021-004036.supp3 Supplementary data

### Updated antitumor activity

**Figure 1:**
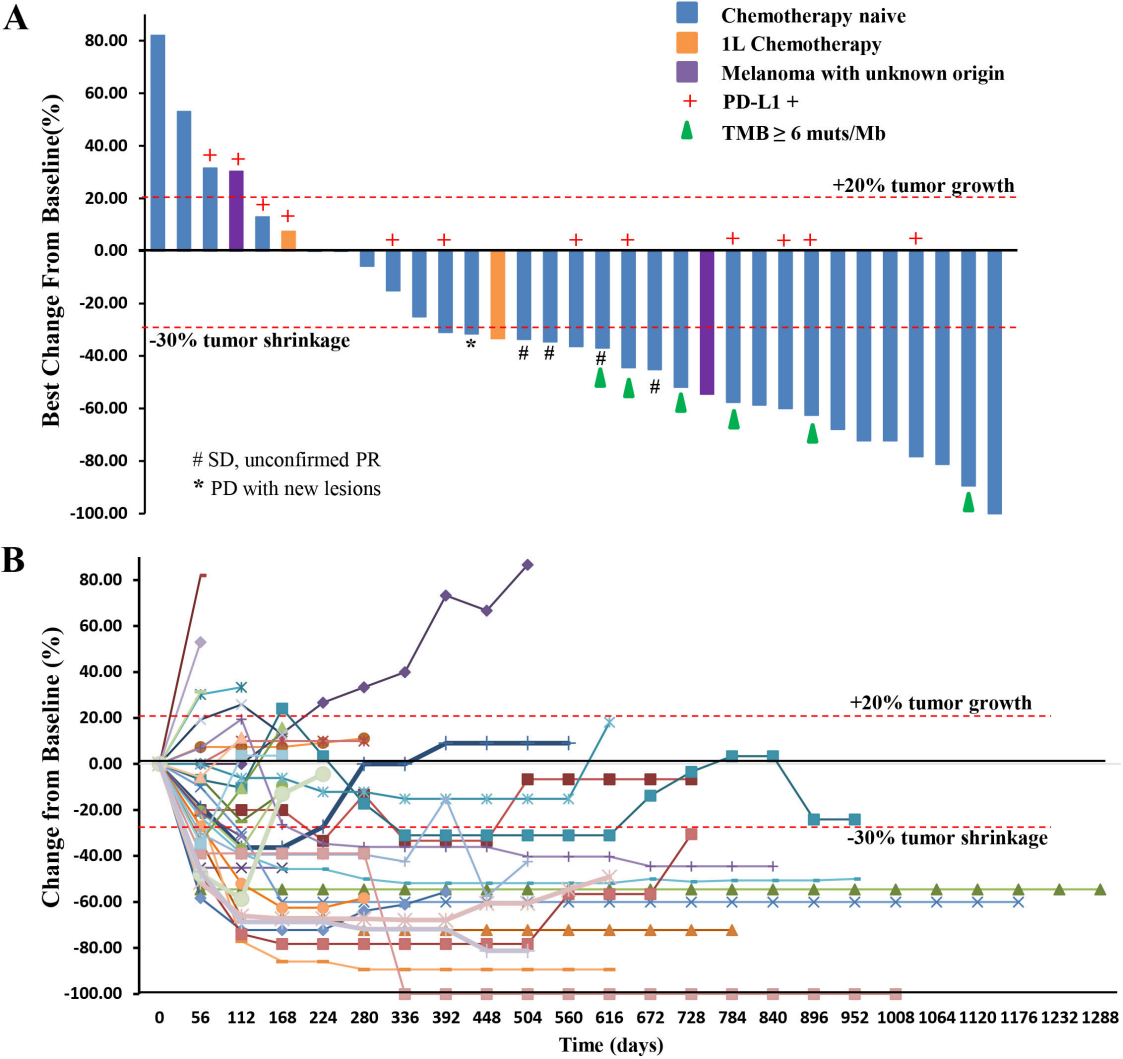
(A) Waterfall plot. Maximal change of tumor size from baseline assessed by investigator per RECIST V.1.1 (n=33). The length of the bar represents maximal decrease or minimal increase in target lesion(s). (B) Spider plot. Change in individual tumor burden over time from baseline assessed by investigator per RECIST V.1.1 (n=33). PD, progressive disease; PD-L1, programmed cell death ligand-1; PR, partial response; RECIST, Response Evaluation Criteria in Solid Tumors; SD, stable disease; TMB, tumor mutational burden.

**Figure 2:**
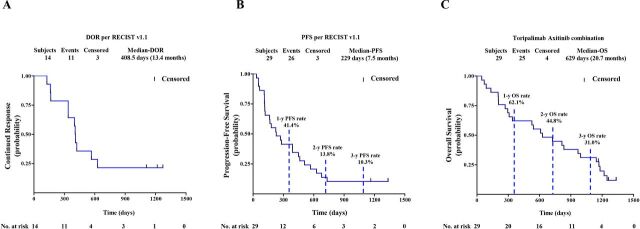
(A) DoR by RECIST V.1.1. (B) PFS by Response Evaluation Criteria in Solid Tumors V.1.1. (C) OS of 29 patients with chemotherapy-naïve mucosal melanoma. Probability of survival is shown at indicated time points. Censored patients are marked with a vertical line in the graph. Numbers of patients at risk at indicated time points are shown below the x-axis. DoR, duration of response; OS, overall survival; PFS, progression-free survival.

Among the 29 chemotherapy-naïve patients with pathologically confirmed MM, one patient, who previously experienced a partial response (PR) as the best response, responded further and became a confirmed complete response (CR) assessed by both RECIST and irRECIST. The overall responses per RECIST included 1 CR, 13 PRs, 11 stable diseases (SDs), and 4 PDs (figure 1A, B). The overall responses per irRECIST included 1 CR, 14 PR, 10 SD, and 4 PD. The ORR by RECIST and irRECIST were 48.3% (95% CI 29.4% to 67.5%) and 51.7% (95% CI 32.5% to 70.6%), respectively. The mDoR was immature in the first report. As of April 02, 2021, the mDoR per RECIST was 13.4 months (95% CI, 5.5 to 20.6 months). The median PFS per RECIST was 7.5 months (95% CI, 3.8 to 14.8 months). The PFS rates of 1, 2, and 3 years per RECIST were 41.4% (95% CI 23.65% to 58.27%), 13.8% (95% CI 4.35% to 28.61%), and 10.3% (95% CI 2.63% to 24.30%), respectively (figure 2A, B).

### Updated OS

By the cut-off date of the first report, 10 of 29 chemotherapy-naïve patients had died and the median OS was not reached. During the additional 28-month follow-up period after the first analysis, 15 additional OS events were recorded. After a median survival follow-up time of 42.5 months (range, 1.47 to 43.74 months), the OS rates of 1, 2, and 3 years were 62.1% (95% CI 42.06% to 76.90%), 44.8% (95% CI 26.52% to 61.57%) and 31.0% (95% CI 15.56% to 47.91%), respectively (figure 2C). The median OS was 20.7 months (95% CI 9.7 to 32.7 months).

### Biomarker analysis

**Figure 3:**
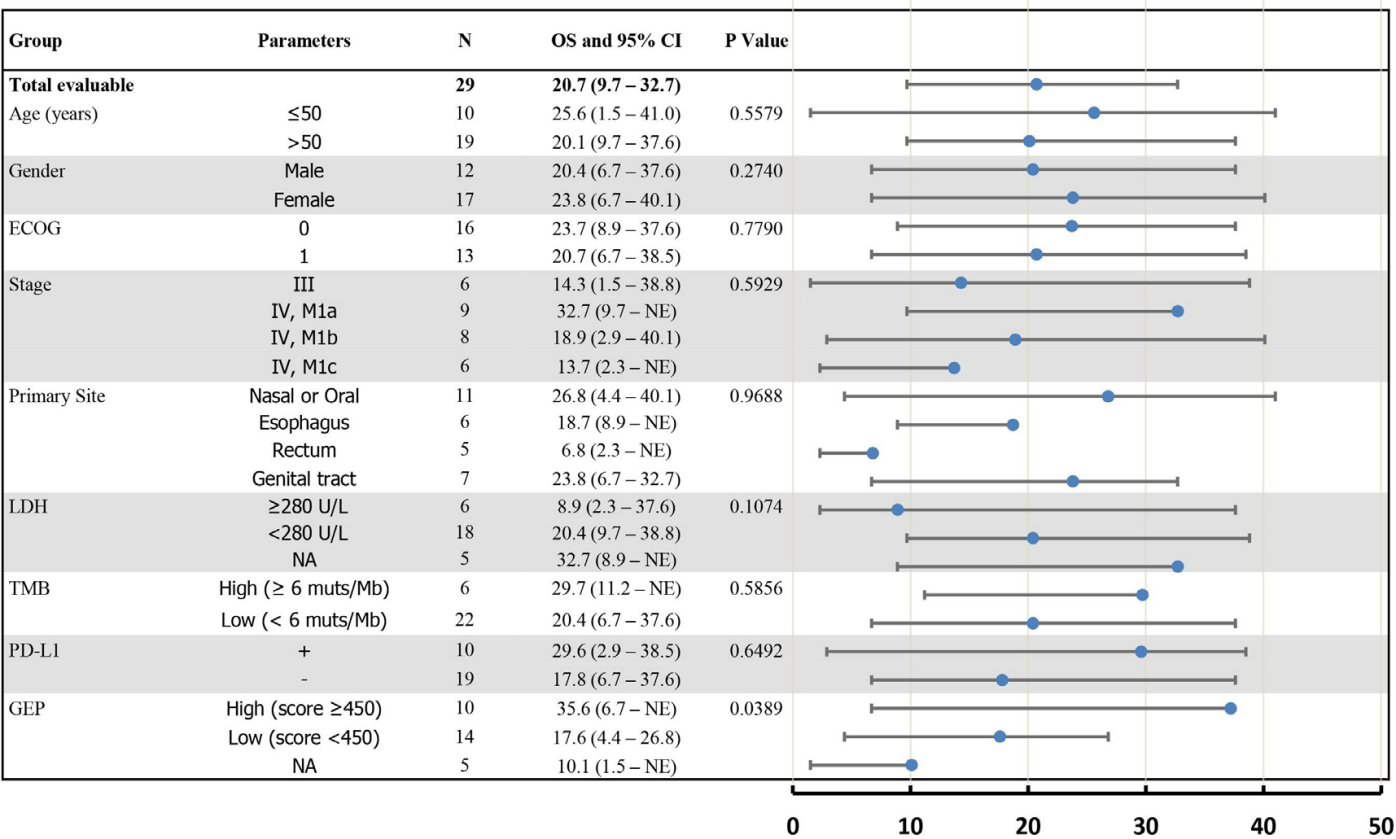
Forest plot. Subgroup analysis of overall survival of 29 patients with chemotherapy-naïve mucosal melanoma. The scale is months (0–50) in the forest plot. ECOG, Eastern Cooperative Oncology Group; GEP, gene expression profile; LDH, lactate dehydrogenase; NA, not applicable; OS, overall survival; PD-L1, programmed cell death ligand-1; TMB, Tumor mutational burden.

We conducted exploratory studies to evaluate the correlation of baseline biomarkers with OS and PFS in the chemotherapy-naïve patients with MM. The treatment effects on OS were analyzed across key subgroups (figure 3).

#### PD-L1 expression

**Figure 4:**
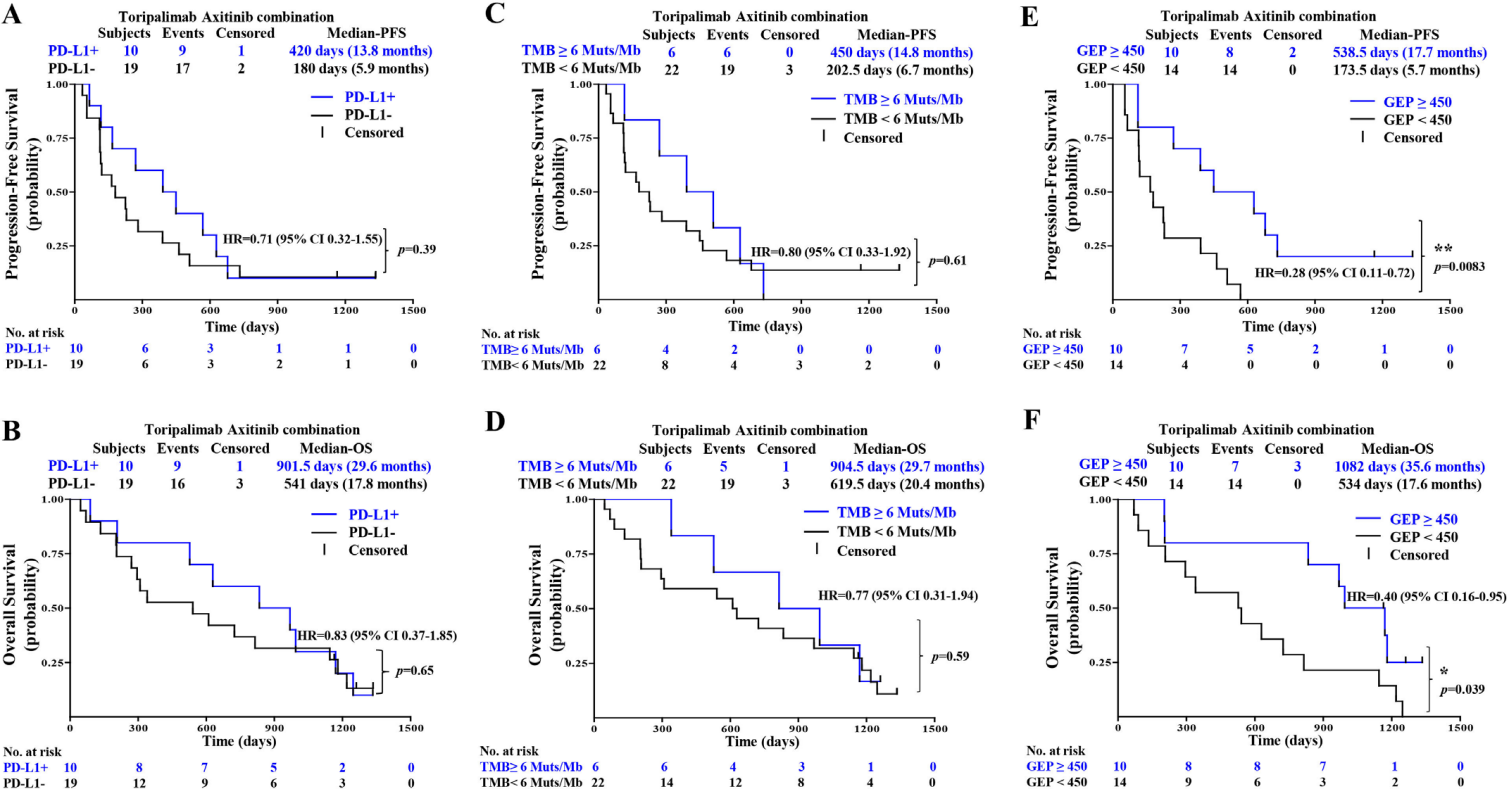
PFS and OS by biomarkers include (A, B) PD-L1, (C, D) TMB, and (E, F) GEP. GEP, gene expression profile; OS, overall survival; PFS, progression-free survival; PD-L1, programmed cell death ligand-1; TMB, tumor mutational burden

As indicated in the first report of this study, PD-L1-positive patients responded with better ORR and PFS than PD-L1-negative patients to the combination therapy.^20^ Follow-up results showed no significant differences in PFS and OS between PD-L1-positive and PD-L1-negative patients: median PFS of 13.8 vs 5.9 months (HR 0.71, 95% CI 0.32 to 1.55; p=0.39) and median OS of 29.6 vs 17.8 months (HR 0.83, 95% CI 0.37 to 1.85; p=0.65) (figure 4A, B).

#### Tumor mutational burden

WES of 28 baseline tumors and matched peripheral blood showed that TMB was generally low in patients with MM in this study, with no patients with TMB greater than 20 mutations/Mbp (range 0.5–15.3 mutations/Mbp). A cut-off of the top 20% of TMB in this study (6 mutations/Mbp) was selected, as suggested by Samstein *et al*,^24^ after a correlation study of TMB value with survival in multiple cancer types. In this follow-up report, no significant differences in PFS and OS were identified between TMB^High^ (≥6 mutations/Mbp) and TMB^Low^ (<6 mutations/Mbp) patients: median PFS 14.8 vs 6.7 months (HR 0.80, 95% CI 0.33 to 1.92; p=0.61) (figure 4C) and median OS 29.7 months vs 20.4 months (HR 0.77, 95% CI 0.31 to 1.94; p=0.59) (figure 4D).

#### 12-gene gene expression profiling (GEP) score

RNA sequencing and expression profiling results were available from 24 patients with chemotherapy-naïve MM. Three published signatures were compared with clinical outcomes as shown in online supplemental figure 3, including inflammation signature (IL-6, CXCL1, CXCL2, CXCL3, CXCL8, and PTGS2),^22^ angiogenesis signature (VEGFA, KDR, ESM1, PECAM1, ANGPTL4, and CD34),^22^ and IFN-γ signature (IDO1, CXCL10, CXCL9, HLA-DRA, STAT1, and IFNG).^23^ However, none of the expression signature scores are significantly different between responder (CR+PR) and non-responder (SD+PD). The 12-gene signatures combined eight selected immune-related genes (CD274/PD-L1, CXCR6, CD27, CXCL9, IDO1, TIGIT, PDCD1LG2/PD-L2, and LAG3) with four angiogenesis-related genes (ANGPTL5, ANGPTL6, CD34, and KDR) and were thus selected and evaluated with efficacy.^22 23^ The GEP score value of 450 was used as the cut-off in this study. The GEP cut-off of 450 was chosen so that the ORRs were 100% in the GEP high group and 0% in GEP low group. Patients with a GEP of ≥450 had a statistically significant improvement in PFS (median PFS 17.7 vs 5.7 months: HR 0.28, 95% CI 0.11 to 0.72; p=0.0083) and OS (median OS 35.6 vs 17.6 months: HR 0.40, 95% CI 0.16 to 0.95; p=0.039) when compared with those with GEP of <450.

#### Other biomarkers

Analysis of WES data shows that the prevalence of mutations in MM is relatively low, and no significant differential mutation profiling is observed between responders and non-responders. Baseline-level lactate dehydrogenase, which was deemed as a prognostic predictor for CM, had no significant impact on PFS and OS in this study (figure 3).

## Discussion

Establishing guidelines for the treatment of MM has been challenging due to the rarity of the disease. Chemotherapy was demonstrated to be less effective in MM than in CM.^25^ Antiangiogenic targeted therapy alone has not shown significant improvement compared with chemotherapy in melanoma. A multicenter phase II study of axitinib monotherapy in metastatic melanoma (predominantly CM) showed an ORR of 18.8%, while the median PFS and OS were only 3.8 and 6.6 months, respectively.^26^ We are currently conducting a randomized, three-arm, multicenter phase II study (NCT03941795) in patients with advanced MM to compare the efficacy and safety of toripalimab plus axitinib versus axitinib or toripalimab monotherapy in the first-line setting, which would address the individual contribution in treating MM. In the era of immunotherapy, the historical ORR obtained by a PD-1 inhibitor single-agent in MM was only 0%–23.3%,^10–12^ while the median PFS ranged from 1.9 to 2.8 months and the median OS ranged from 10.3 months to 11.3 months.^10–12^ The current study is the first to combine immunotherapy with antiangiogenic targeted therapy in treatment-naïve advanced MM. The combination had a tolerable safety profile and showed promising antitumor activity with an ORR of 48.3%, a median PFS of 7.5 months and a median OS rate of 20.7 months. The response was durable as the mDoR was 13.4 months.

As for the dual blockade of CTLA-4 and PD-1 pathways, a pooled analysis showed that among patients with MM who received the combination of nivolumab plus ipilimumab, the ORR (37.1%) and the median PFS (5.9 months) were only slightly improved than CTLA-4 or PD-1 blockade alone,^16^ while the median OS was not mature. Recently, the phase III CheckMate 067 study released the data from a subgroup analysis of MM.^17^ The ORRs were 7%, 30%, and 43%, in three arms treated with ipilimumab, nivolumab, and ipilimumab plus nivolumab, respectively. The median OS were 20.2 and 22.7 months in the nivolumab and ipilimumab plus nivolumab arms, respectively, while the median PFS were only 3.0 and 5.8 months. In CheckMate 067, after a minimum follow-up of 5 years in patients with untreated advanced melanoma (predominantly CM), the mDoR was not reached in the nivolumab monotherapy and the nivolumab plus ipilimumab group. The ongoing responses at 5 years were 62% and 61%, respectively.^27^ In contrast, in a pooled study evaluating the efficacy of anti-PD-1 agents in MM (n=35), the mDoR was 12.9 months.^28^ The mDoR observed in the current study was 13.4 months, which is similar to the reported anti-PD-1 monotherapy in MM but much shorter than that of CM, reflecting the divergent responses of these two melanoma subtypes to immunotherapy.

The OS result of the axitinib plus toripalimab was comparable to that of ipilimumab plus nivolumab (20.7 months vs 22.7 months). However, there were several major differences between the patients from the current study and the mucosal subgroup from CheckMate 067 that were treated with ipilimumab plus nivolumab. The patients were predominantly Caucasian in CM-067, while all were Asian in the current study. Sixty-eight percent of the patients in CM-067 had stage IV M1C disease, while only 18% of patients from the current study had M1C disease. It remains to be determined in a randomized trial which combination strategy will be the preferred first-line regimen for MM.

Compared with the limited efficacy by anti-PD-1 or axitinib monotherapy, the improved efficacy in this study showed synergistic effects of an antiangiogenic drug with immunotherapy. According to the theory of cancer-immunity cycle, activated T cells need to be trafficked and infiltrated into the tumor, and only when activated T cells overcome local inhibitory factors, in the tumor microenvironment (TME), they can recognize and eliminate tumor cells.^29^ The use of anti-VEGF-targeted drugs could enhance T-cell infiltration into the tumor and overcome the inhibition from the immune microenvironment. The theory of tumor vasculature normalization also supports this theory. Many studies showed that the use of anti-VEGF-targeted drugs can promote the normalization of tumor vasculature that can increase the infiltration of immune effector cells into tumors and convert the intrinsically immunosuppressive TME to become immunostimulatory.^30^ Thus, combining antiangiogenic therapies and immunotherapies might synergistically increase the effectiveness of immunotherapy.

Besides the combination with immunotherapy in the current study, antiangiogenic therapy also showed significant benefits when combined with chemotherapy in MM. In the phase II study, untreated patients with advanced MM were 2:1 randomized to receive front-line carboplatin plus paclitaxel with or without bevacizumab. Although the ORR was not statistically different, both the median PFS and median OS were significantly improved in the combination arm. Although the front-line anti-PD-1-based immunotherapy remains the preferred approach for advanced CM without BRAF mutations, incorporating VEGF-targeting therapy with immunotherapy could potentially improve the clinical response in patients with MM.

We also evaluated the predictive values of tumor PD-L1 expression, TMB, and inflammation and angiogenesis expression signatures for survival. In the first report of this study, PD-L1-positive expression was associated with significantly longer PFS. In this updated analysis, the OS between the PD-L1 and TMB subgroups had no statistically significant differences. The SP263 antibody was used for PD-L1 IHC staining in the study as it has shown concordant staining results with other commonly used PD-L1 IHC antibodies, including 22C3 and 28–8.^31^

PD-L1 expression has not been a reliable biomarker in predicting the clinical benefits of ICIs. Several studies have found no correlation between tumor PD-L1 expression and the clinical efficacy of ICIs, and some patients with negative PD-L1 expression have also achieved durable clinical benefit.^32 33^ Moreover, PD-L1 IHC staining method has several limitations,^34^ including the heterogeneity of PD-L1 expression, no standardized approach for PD-L1 testing, and the availability of tumor tissues.

TMB is used to quantify the number of somatic mutations in human tumors. A higher TMB value correlates with a higher frequency of neoantigens^35^ and a more favorable response to ICIs in certain solid tumors. However, TMB is not correlated with clinical efficacy of ICIs in several tumor types, such as breast cancer, glioma, and prostate cancer. A study performed WES on 294 microsatellite stable tumors (including 151 melanomas) and concluded that TMB did not have sufficient predictive power to distinguish tumor response from PD.^36^ Prediction incorporating multiple variables, such as TMB, MHC haplotype and T-cell receptor repertoire, might be needed.^35^ On the other hand, MM was demonstrated to be a low-TMB tumor,^9^ which may explain the lack of clinical efficacy correlation with TMB in this study.

Different from the correlation of a single biomarker with clinical outcomes, GEP comprehensively describes the characteristics of TMEs, incorporating multiple pathways related to antigen presentation, chemokine expression, cytolytic activity, and adaptive immune resistance.^23^ In the KEYNOTE 001 trial, Ayers *et al* used an IFN-γ signature (six genes including IDO1, CXCL10, CXCL9, HLA-DRA, STAT1, and IFN-γ) and an expanded immune (18-gene) signature to evaluate the correlation between gene signatures and clinical outcomes in a cohort of 62 patients with melanoma receiving pembrolizumab monotherapy. They found that these two sets of gene signatures were significantly associated with ORR and PFS benefits.^23^

However, previous GEP studies were primarily focused on CM, and the application of GEP to predict response to immunotherapy in MM remains unknown. Furthermore, unlike anti-PD-1 monotherapy, VEGFR-tyrosine kinase inhibitor and anti-PD-1 combination possibly needs tailored gene expression signatures specific to the combination regimens to predict response.

The 12-gene expression signatures in the current study were derived from panels with known association clinical benefits (McDermott *et al*^22^ and Ayers *et al*^23^) and were selected based on the best differential fit (responder vs non-responder). We also compared three published signatures with clinical outcomes as described in the Methods section and shown in online supplemental figure 3, including inflammation signature,^22^ angiogenesis signature,^22^ and IFN-γ signature.^23^ None of the expression signatures could significantly differentiate responders from non-responders. It is possible that an MM with a low mutational burden might compromise the predictability of these signatures. A derived panel to include genes involved in both immune regulation/inflammation and angiogenesis might be more suitable to predict the clinical response of the combination therapy. The 12-gene expression signatures of eight immune-related genes (CD274/PD-L1, CXCR6, CD27, CXCL9, IDO1, TIGIT, PDCD1LG2/PD-L2, and LAG3) and four angiogenesis-related genes (ANGPTL5, ANGPTL6, CD34, and KDR) were thus selected to construct a logistic regression model to differentiate patients with different efficacy.^22 23^ The inflammation and angiogenesis signature GEP scores were found to be associated with improved ORR and DCR in the first report of this study. In this updated analysis, patients with GEP of ≥450 had statistically significant longer PFS (17.7 vs 5.7 months) and OS (35.6 vs 17.6 months) than those with GEP of <450. To our knowledge, this is the first study reporting the utility of GEP to predict not only the ORR and PFS but also OS benefits in response to the combination of an anti-VEGF therapy plus an ICI therapy in patients with MM. Nevertheless, the utility of the 12-gene GEP to predict clinical response to the combination of axitinib and toripalimab requires further validation in a prospective randomized trial.

In conclusion, this updated report confirms the antitumor activity of the combination of toripalimab with axitinib in patients with advanced MM, including long-term survival benefits. A limitation of the current study is that the efficacy evaluation was assessed by the investigator in a single-arm study. The clinical efficacy of the combination therapy as well as the utility of the 12-gene GEP to predict clinical response is yet to be confirmed by an independent central radiology review in the phase III trial of toripalimab plus axitinib versus pembrolizumab as first-line treatment for patients with advanced MM (NCT04394975).

10.1136/jitc-2021-004036.supp4 Supplementary data

## Data Availability

Data are available upon reasonable request. The datasets used and/or analyzed during the current study are available from the corresponding author on reasonable request.
