# Supplementary material for: Toripalimab plus axitinib in patients with metastatic mucosal melanoma: 3-year survival update and biomarker analysis
Source: J Immunother Cancer. 2022 Feb 21;10(2):e004036. doi: 10.1136/jitc-2021-004036 (PMC9066368; doi:10.1136/jitc-2021-004036)
Supplement: Supplementary data [file jitc-2021-004036supp001.pdf]

Supplemental Figure 1 Design of phase IB combination study of toripalimab plus axitinib in treating patients with mucosal melanoma and clinical efficacy summary. DLT, dose-limiting toxicity; IV, intravenous; PD, progressive disease; PR, partial response; SD, stable disease.

Supplemental Figure 2 CONSORT diagram for phase IB combination study of toripalimab with axitinib in treating patients with mucosal melanoma.

Supplemental Figure 3 The correlation of clinical response with inflammation or angiogenesis signature panel scores. McDermott et al. [22] inflammation panel: IL-6, CXCL1, CXCL2, CXCL3, CXCL8, and PTGS2; McDermott et al. [22] angiogenesis panel: VEGFA, KDR, ESM1, PECAM1, ANGPTL4, and CD34; Ayers et al. [23] inflammation panel: IDO1, CXCL10, CXCL9, HLADRA, STAT1, and IFN-γ. Responders are those with complete or partial response; non-responders are those with stable or progressive disease.

Li S, *et al. J Immunother Cancer* 2022; 10:e004036. doi: 10.1136/jitc-2021-004036
