# Supplementary figures and images for: Toripalimab plus axitinib in patients with metastatic mucosal melanoma: 3-year survival update and biomarker analysis
Source: J Immunother Cancer. 2022 Feb 21;10(2):e004036. doi: 10.1136/jitc-2021-004036 (PMC9066368; doi:10.1136/jitc-2021-004036)

## Supplemental Figure 1

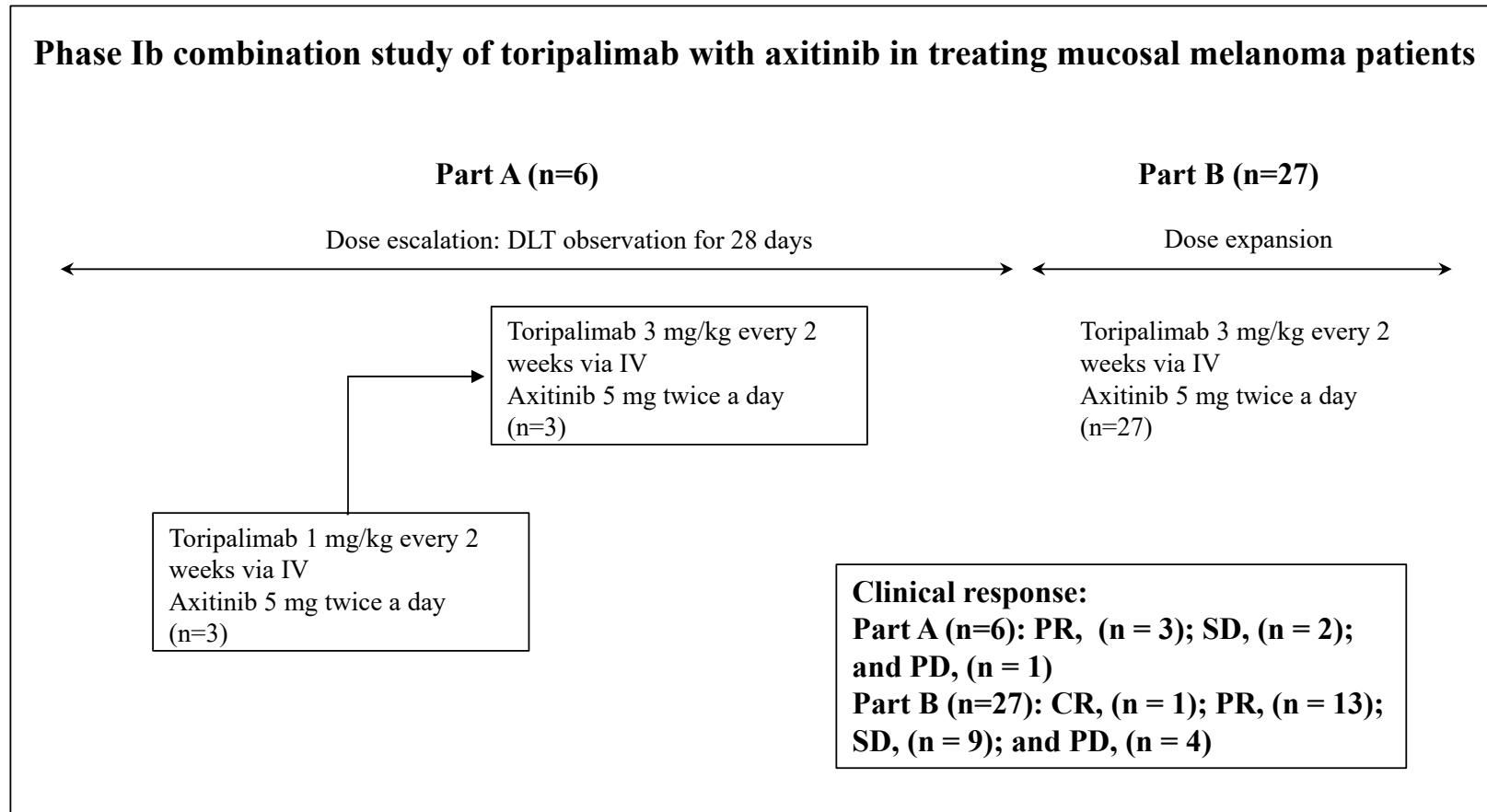

## Supplemental Figure 2

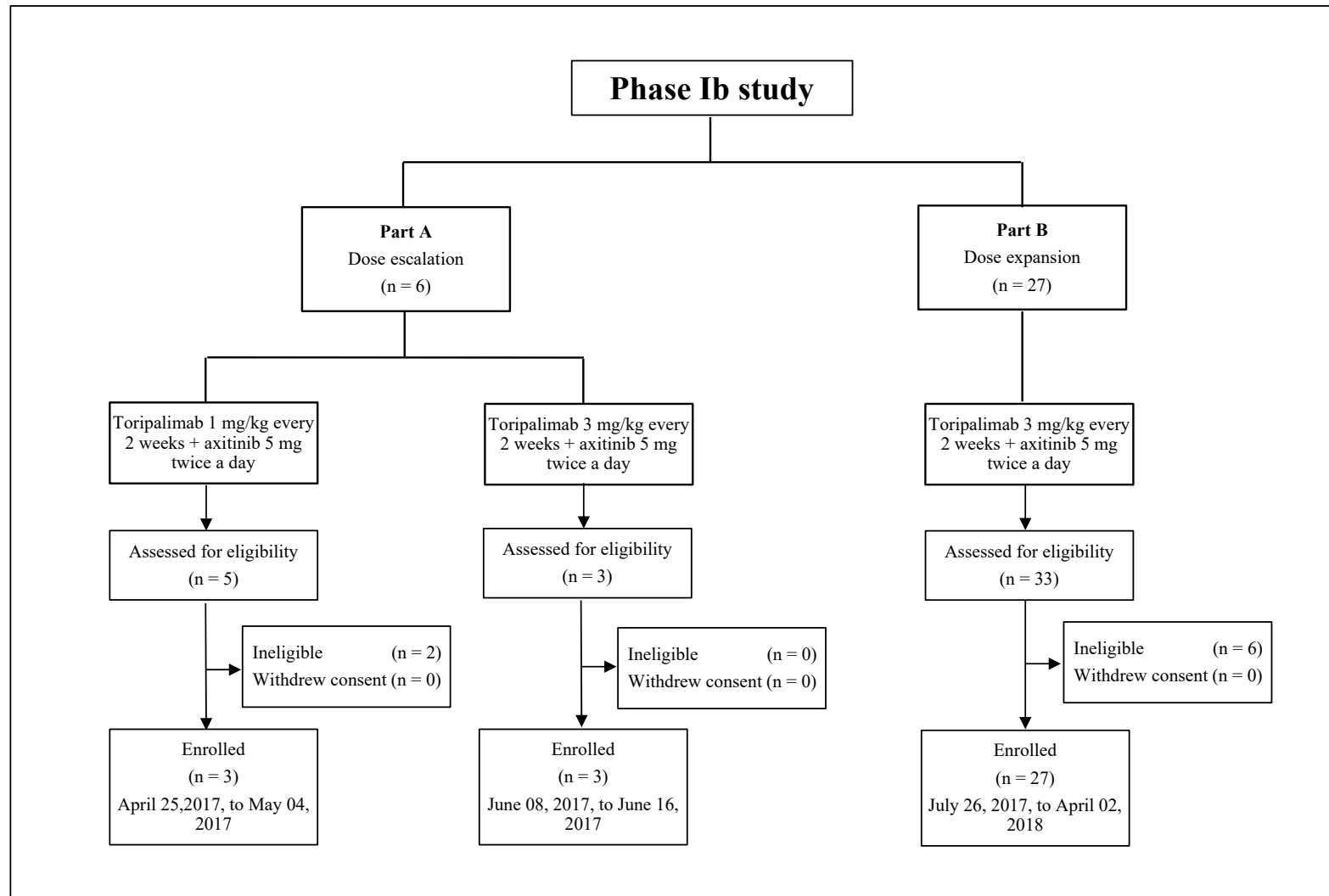

## Supplemental Figure 3

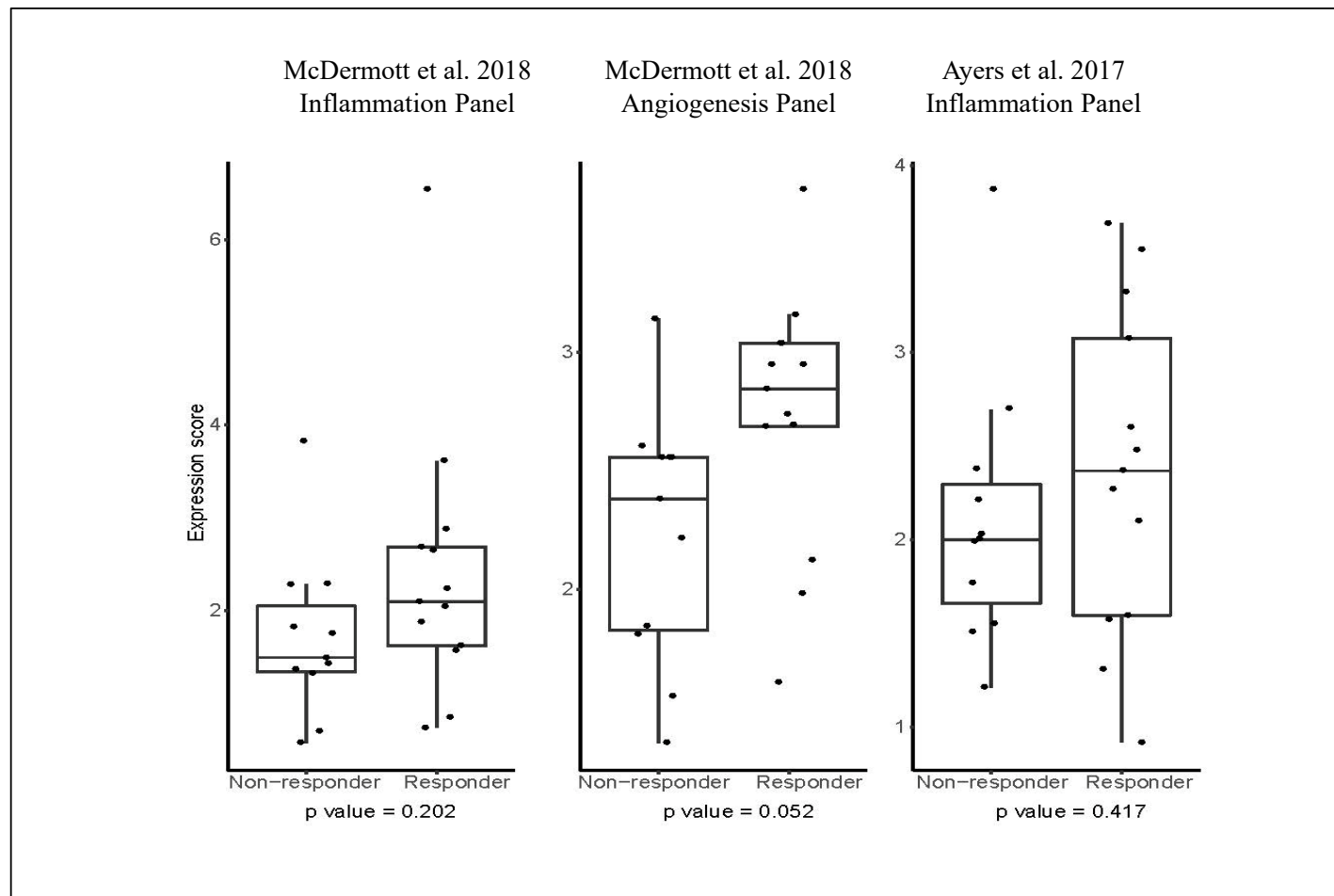

Supplement: Supplementary data [file jitc-2021-004036supp002.pdf]
