# Supplementary material for: Toripalimab plus axitinib in patients with metastatic mucosal melanoma: 3-year survival update and biomarker analysis
Source: J Immunother Cancer. 2022 Feb 21;10(2):e004036. doi: 10.1136/jitc-2021-004036 (PMC9066368; doi:10.1136/jitc-2021-004036)
Supplement: Supplementary data [file jitc-2021-004036supp003.pdf]

Supplemental Table 1. Baseline characteristics of patients with mucosal melanoma (*N* = 33).

**Characteristic**	**Number of patients, %**
Age, years	
Median	54.0
Range	27.0 - 70.0
Sex	
Male	13 (39.4)
Female	20 (60.6)
ECOG performance status	
0	20 (60.6)
1	13 (39.4)
Prior systemic chemotherapy	
None	31 (93.9)
First line	2 (6.1)
Location of primary lesion	
Nasal cavity	6 (18.2)
Oral cavity	5 (15.2)
Esophagus	7 (21.2)
Genital tract	7 (21.2)
Rectum	5 (15.2)
Unknown	2 (6.1)
Sinus	1 (3.1)
Stage at treatment	
III	7 (21.2)
IV, M1a	9 (27.3)
IV, M1b	11 (33.3)
IV, M1c	6 (18.2)
Prior systemic therapy	
Yes	2 (6.1)
No	31 (93.9)
LDH	
≤ ULN	24 (72.7)
> ULN and < 2 × ULN	9 (27.3)
> 2 × ULN	0
Mutation Status	
*BRAF**	1 (3.0)
RAS	9 (27.3)
NF1	4 (12.1)
BRAF/RAS/NF1 triple wild type	20 (60.6)
KIT	6 (18.2)
PD-L1 Result†	
Positive	12 (36.4)
Negative	21 (64.6)

NOTE: Values are numbers and percentages unless otherwise noted.

Abbreviations: ECOG, Eastern Cooperative Oncology Group; LDH, lactate dehydrogenase; PD-L1, programmed death ligand-1; ULN, upper limit of normal.
*BRAF mutant, n = 1 (V600E).
†Positive was defined as 1% or more of tissue cells expressing PD-L1 by SP263 immunohistochemistry staining.
