# Supplementary material for: Toripalimab plus axitinib in patients with metastatic mucosal melanoma: 3-year survival update and biomarker analysis
Source: J Immunother Cancer. 2022 Feb 21;10(2):e004036. doi: 10.1136/jitc-2021-004036 (PMC9066368; doi:10.1136/jitc-2021-004036)
Supplement: Supplementary data [file jitc-2021-004036supp004.pdf]

Clinical Trial Protocol

Tolerability and Pharmacokinetics of JS001 combined with Axitinib in Advanced Renal Cell Carcinoma and Advanced Melanoma: A Single-center, Open-Label, Dose Escalation, Phase Ib Clinical Study

Study Protocol Number：HMO-JS001-Ib-CRP-01

Protocol Version：1.2

Version Date：April 14, 2017

Confidentiality Statement

This document contains confidential information of Shanghai Junshi Biosciences Co., Ltd. and Suzhou Zhonghe Biosciences Co., Ltd., and it shall only be used for this clinical study purposes. Disclosure to anyone except for the staff participating the study and members of Institutional Review Board should be strictly prohibited. This information should not be used for any purpose other than evaluation or conduct of the clinical study without written authorization from Shanghai Junshi Biosciences Co., Ltd and Suzhou Zhonghe Biosciences Co., Ltd.

JS001 (V1.2/April 14, 2017)   Protocol Number：HMO-JS001-Ib-CRP-01

Investigator's Statement

I have read the clinical study protocol entitled "Tolerability and Pharmacokinetics of JS001 combined with Axitinib in Advanced Renal Cell Carcinoma and Advanced Melanoma After Standard Treatment Failure: A Single-center, Open-Label, Dose Escalation, Phase Ib Clinical Study" (Version 1.2, Date：April 14, 2017), and I agree to abide by all the terms set forth therein.

I agree to follow the guidelines in Good Clinical Practice and other applicable CFDA provisions/guidelines.

I agree to ensure that the following persons complete the financial disclosure statement before the study begins, during the study period, if there is a change affecting my financial disclosure status, and after the study:

● I (including - if applicable - my spouse [or legal partner] and dependent children)

● My assistant investigator (including - if applicable - their spouse [or legal partner] and dependent children).

I am hereby to guarantee that I will not use the confidential information contained in this document for any purposes other than the evaluations or conduct of this clinical study without written authorization from Shanghai Junshi Biosciences Co., Ltd. and Suzhou Zhonghe Biosciences Co., Ltd.

Name of Principal Investigator：Jun Guo

Signature: **Date:**

Sponsor's Statement

1．Sponsor

I am hereby to guarantee that the responsibilities of the sponsor, including initiation, application, organization, funding and monitoring of this clinical study will be fulfilled according to China GCP, and active treatments will be provided at our own cost to subjects who developed any serious adverse event related to the study drug during the course of clinical study in conformity with the stipulations in the applicable national laws and regulations, and reasonable economic compensation will be provided for any impairment caused by any serious adverse reaction which is indeed induced by the study drug. And this clinical study will be conducted according to this protocol design and requirements.

Sponsor： Shanghai Junshi Biosciences Co., Ltd.
Suzhou Zhonghe Biosciences Co., Ltd.

Project Leader (Signature): __________________ Date: ______________________

2．Contract Research Organization (CRO)

I will fulfill the duties of the CRO in accordance with the "Quality Management Regulations for Clinical Trials" and other relevant regulations, organize the implementation of this clinical trial on the basis of standardized procedures, and conduct audits, data management, quality control and assurance for clinical trials.

CRO： Beijing Halma Orient Medicine Technology Co. Ltd

Project Leader (Signature): __________________ Date: ______________________

Organization of the clinical trial

**Sponsor**	Shanghai Junshi Biosciences Co., Ltd.<br>Suzhou Zhonghe Biosciences Co., Ltd.<br>Tel：0512-86876925 Fax：0512-86876920
**Sponsor Contact**	Miaoxin Wang<br>Tel：15905191313
**Clinical research Unit**	Beijing Cancer Hospital
**Principal Investigator**	Prof. Jun Guo<br>Tel：010-88140650/13911233048
**Contract Research Organization (CRO)**	Beijing Halma Orient Medicine Technology Co. Ltd<br>Tel：010-82255819 Fax：010-82255829
**Inspector**	Huali Huang Tel：13693315578
**CRA**	Shumin Cui Tel：13810695428
**Data management and statistical unit**	Beijing Halma Orient Medicine Technology Co. Ltd
**Pharmacokinetics analysis unit**	United-Power Pharma tech Co., Ltd
**PI of Pharmacokinetics**	Lun Ou, Tel: 010-80704457-8001/13651324734

**analysis**	
**Clinical Center Laboratory for Biological Sample (blood/tissue)**	Beijing Cancer Hospital and United-Power Pharma tech Co., Ltd

Protocol Synopsis

**Title**	Tolerability and Pharmacokinetics of JS001 combined with Axitinib in Advanced Renal Cell Carcinoma and Advanced Melanoma: A Single-center, Open-Label, Dose Escalation, Phase Ib Clinical Study
**Protocol Number**	HMO-JS001-Ib-CRP-01
**Study Phase**	Phase Ib
**Trial Duration**	February 2017 – February 2019
**Study Objectives**	**Primary Objective**<br>To evaluate the tolerability and safety of JS001 (a recombinant humanized anti-PD-1 monoclonal antibody) combined with axitinib in advanced renal cell carcinoma and advanced melanoma naïve to systemic therapy or had limited prior systemic therapy. To evaluate dose-limiting toxicity (DLT), maximum tolerated dose (MTD) and recommended dose (RD) to provide the basis for the development of a dosing regimen for later stage of clinical trials.<br>**Secondary Objectives**<br>• To evaluate the pharmacokinetics of JS001 combined with axitinib after administration<br>• To evaluate the anti-tumor activity of JS001 combined with axitinib after administration.<br>• To evaluate the effect on the PD-1 target, and the association between drug metabolism and drug inhibitory functions, by evaluating PD-1 receptor occupancy of T lymphocytes in blood samples after administration.<br>• To investigate the immunogenicity of JS001, observe the changes of anti-

	drug antibody production in vivo after administration, and provide reference for the safety and efficacy of the drugs.<br>• To provide reference for population selection by detecting the expression of PD-L1 in tumor tissue samples and conducting correlational analysis of PD-L1 expression with clinical efficacy.
**Study Endpoints**	**Primary Endpoint**<br>Safety: incidence and severity of adverse events, and clinically significant laboratory abnormalities, ECG and vital signs.<br>**Secondary Endpoint**<br>• Pharmacokinetic（PK）parameters; Single dose C_{max}, T_{max} and AUC_{0-T}, $AUC_{0-\infty}$, $t_{1/2}$, CL, V; C_{min} on steady state after multiple administrations, C_{av}, fluctuation factor (DF)，Vss, etc.<br>• Target-related pharmacodynamic indicators, such as PD-1 receptor occupancy in the blood.<br>• Objective response rate (ORR) evaluated based on Response Evaluation Criteria in Solid Tumors (RECIST 1.1) and Immune-Related Response Criteria (irRC).<br>• Duration of response（DOR）.<br>• Disease control rate（DCR）.<br>• Time to response（TTR）.<br>• Progression free survival（PFS）.<br>• Overall survival（OS）.<br>**Exploratory Endpoints**<br>• Correlation analysis of PD-L1 expression in tumor tissue with the anti-tumor activity.
**Study Design**	This study is the first human study of recombinant humanized anti-PD-1 monoclonal antibody (JS001) combined with axitinib. It's designed to be a single-center, open-label, Dose Escalation, Phase Ib clinical study. It intends to enroll advanced RCC and melanoma patients. The study is divided into two parts: the dose escalation phase and the expansion phase.<br>The purpose of the dose escalation phase is to evaluate the safety and tolerability of JS001 combined with axitinib, the pharmacokinetics of JS001 and dose-limiting toxicity (DLT), maximum tolerated dose (MTD)/recommended dose (RD) of the combined therapy. Up to 18-24 patients is planned to be enrolled in this phase of the study.

JS001 (V1.2/April 14, 2017) **Protocol Number：HMO-JS001-Ib-CRP-01**

	At the end of the dose escalation phase, additional RCC and melanoma subjects will be enrolled in the expansion phase. For efficacy evaluation in the expansion phase, there will be at least 10 subjects in the recommended dose group. We will further explore the pharmacokinetic characteristics, dose, efficacy and safety of JS001 after co-administration with axitinib.
**Dose escalation method**	After determining the eligibility of the subjects, the subjects are assigned to a certain dose group according to the order in which they are selected. Dose is escalated independently in each disease. If DLT occurs at the initial dose, the dose is decreased to 0.3 mg/kg. At least 3 subjects were enrolled in each group, and at least 6 subjects were enrolled at the MTD dose level. The maximum dose of JS001 is 3 mg/kg. After climbing to the maximum dose, the sponsor and the investigator will make a decision whether continue dose escalation or initiate an intermediate dose group based on previous observations.<br><br>The dose escalation will be performed based on the 3+3 principle. Dose escalation is applied individually in each type of tumor (Mucosal melanoma, acral melanoma and renal cell carcinoma). The DLT incidence is monitored in the first 28 days after treatment, and DLT safety evaluation is performed on the 29th day. The toxicity is graded using the NCI-CTCAE 4.0 standard. (See the DLT definition below for details). The dose escalation method is described as follows:<br><br>- If the first 3 subjects in the first dose level group (JS001 1 mg/kg Q2W + axitinib 5 mg Bid) do not develop DLT within the first 28 days of treatment, the dose escalation will continue, and 3 subjects will be enrolled to the second group (JS001 3 mg/kg Q2W + axitinib 5 mg Bid).<br><br>- If one of the 3 subjects has a DLT, 3 subjects should be added to this dose group. If there is no DLT in the 3 new subjects (only 1 of 6 cases), then enter the next higher dose group.<br><br>- If DLT occur in 2 or more of the first 6 subjects in the 1 mg/kg JS001 dose group, DLT has reached and no higher dose will be initiated, and the protocol will be reassessed.<br><br>- If the first 3 subjects with JS001 dose escalation to the 3 mg/kg group do not develop DLT or 5 of the first 6 subjects did not develop DLT, the dose escalation is completed and the 3 mg/kg Q2W + axitinib 5 mg Bid dose will be the recommended dose for the study.<br><br>- If 2 of the 6 subjects in the JS001 3 mg/kg group achieve DLT criteria, the axitinib 5mg Bid is reduced to 5 mg QD to continue the dose escalation evaluation. If the first 3 subjects or 5 of the first 6 subjects do not develop DLT, the dose escalation is completed and JS001 3 mg/kg Q2W + axitinib 5 mg QD will be the recommended dose for the study.<br><br>- If 2 of the 6 subjects in the JS001 3mg/kg Q2W + axitinib 5mg QD dose group meet the DLT criteria, JS001 1mg/kg Q2W + axitinib 5mg QD will be the

	recommended dose for the study.<br>- After determining the recommended dose, an expansion phase will be performed at this dose.<br>At the end of the DLT observation period, patients with no disease progression and no DLTs will be determined by the investigator whether they are eligible for continuing treatment. If permitted, they will continue to receive JS001 in combination with axitinib at the same dose level conducted during the dose escalation period. The subject will be treated until disease progression, intolerable toxicity, voluntary withdrawn, or the investigator assesses that the subject can no longer benefit from treatment.
**Withdraw from the trial**	During the study, subjects with disease progression (PD) according to the Immune-Related Response Criteria (irRC) and Response Evaluation Criteria in Solid Tumors (RECIST 1.1) will discontinue from the study. In addition, subjects may withdraw from the study at any time if a DLT or disease progression occurs. Subjects may withdraw from the study at any time for any reason. If a concurrent illness, adverse event, or protocol violation occurs, the investigator can also discontinue the subject from the study.<br>If the study is terminated due to toxicity, the subject will be followed for at least 30 days until any drug-related toxicity returns to grade 1. In the event of an unplanned termination, the investigator should make every effort to contact the subject for a final evaluation.
**Sample size**	In the dose escalation phase of this study, 3-6 cases are expected in each dose group of each tumor, and totally 18-24 cases are expected to be enrolled. The actual sample size depends on the dose escalation study and will be adjusted according to the actual situation. The number of subjects in the expansion phase will also be decided based on the study status in the initial phase.
**Study population**	**Inclusion Criteria：**<br>**Subjects may be entered in the study only if they meet all of the following criteria：**<br>• Male and Female aged between 18 and 75 years;<br>• Easter Cooperative Oncology Group (ECOG) performance status score of 0 or 1;<br>• Histologically confirmed advanced RCC or melanoma who have disease progression or intolerance after at least one dose of routine systemic treatment, or refused routine systemic treatment;<br>• Agree to provide fresh or archival tumor tissue specimens (fresh tumor tissue is preferred for detecting PD-L1 expression and infiltrating lymphocytes);<br>• At least one measurable lesion (Only one measurable lymph node lesion was

excluded) (conventional CT scan ⩾ 20 mm, spiral CT scan ⩾ 10 mm, measurable lesions have not received radiotherapy) ;

- Life expectancy≥3 months；
- Patients may have a history of brain/meningeal metastases, but they must have local treatment (surgery/radiotherapy) prior to the start of the study and be clinically stable for at least 3 months (prior corticosteroids use is allowed, but patients with concurrent use of systemic corticosteroids should be excluded)；
- Sufficient Organ function (within 14 days before enrollment)：

（1）Must meet the blood routine examination standards (no blood transfusion or G-CSF treatment within 14 days)：

HB ≥90 g/L；

ANC≥1.5×10^9 /L；

PLT≥100×10^9 /L；

（2）Non-functional organic diseases, meeting the following criteria：

TBIL≤1.5×ULN（Upper Limit Of Normal）；

ALT and AST≤2.5×ULN； With hepatic metastasis: ALT and AST≤5×ULN；

Serum Cr≤1.25×ULN, and Ccr＞50ml/min（Cockcroft-Gault formula）；

International normalized ratio (INR), activated partial thromboplastin time (aPTT): ⩽1.5×ULN (this standard only applies to patients who have not received anticoagulant therapy; patients who receive anticoagulant therapy should place anticoagulants in the range of treatment requirements);

Urine protein ⩽ 1 +; if the urine protein > 1 +, collect 24 hours urine protein determination, the total amount should ⩽ 1 gram;

- No systemic corticosteroids is received within 4 weeks prior to treatment；
- Males with fertility or women who may become pregnant must use highly effective methods of contraception (such as oral contraceptives, intrauterine devices, libido or barrier contraceptives combined with spermicides) during the trial. Continue contraception for 12 months after the end of the therapy;
- Subjects must join the study voluntarily, must sign informed consent, and agree to comply to follow-up.

Exclusion Criteria

	**Subjects fulfilling any of the following conditions cannot be enrolled in the study:**<br>• Previous treatments with anti-PD-1 antibody, anti-PD-L1 antibody, anti-PD-L2 antibody or axitinib;<br>• Known to be allergic to recombinant humanized anti-PD-1 monoclonal antibody drugs and its components;<br>• Received other anti-tumor treatments (including corticosteroids, immunotherapy) or participating in other clinical studies within 4 weeks prior to the start of treatment, or haven't recovered from the last toxicity (except for grade 2 alopecia and grade 1 neurotoxicity);<br>• Pregnant or lactating;<br>• Positive for HIV;<br>• Active hepatitis B or hepatitis C patients:<br>Positive test for HBsAg, with HBV DNA copies detected as positive (quantitative measurements ⩾ 500 cps/mL); HBV DNA must be detected during screening of such patients;<br>Positive test for chronic Hepatitis C in blood screening test (HCV antibody positive); patients with positive HCV antibody test results can only be included in the study when the PCR results of HCV RNA are negative.<br>• Diagnosed with active tuberculosis (TB)；<br>• With a large amount of pleural or ascites with clinical symptoms or need to be treated symptomatically;<br>• With autoimmune diseases, or history of autoimmune diseases requiring systemic use of steroids/immunosuppressive agents, such as pituitary inflammation, pneumonia, colitis, hepatitis, nephritis, hyperthyroidism, thyroid function decrease, etc.;<br>• Other serious, uncontrollable concomitant diseases that may affect protocol compliance or interfere with the results, including active opportunistic infections or advanced (severe) infections, uncontrolled diabetes, cardiovascular disease (grade III or IV heart failure as defined by the New York Heart Association classification, heart block above grade II, myocardial infarction in the past 6 months, unstable arrhythmia or unstable angina, cerebral infarction within 3 months, or lung disease (interstitial pneumonia, obstructive pulmonary disease, and history of symptomatic bronchospasm);<br>• Subjects with active central nervous system (CNS) metastases. If the subject's CNS metastasis is adequately treated to meet the requirements specified in the inclusion criteria, and the subject's neurological symptoms can be restored to less than or equal to CTCAE grade 1 before enrollment (except for residual signs or symptoms associated with CNS treatment), after at least 2 weeks, the

	subject can participate in the study;<br>• Received hematopoietic stimulating factors such as colony stimulating factor and erythropoietin within 1 week before the start of the study;<br>• Received live vaccination within 4 weeks prior to the start of the study;<br>• Received allogeneic hematopoietic stem cell transplantation or solid organ transplantation;<br>• Received major surgery (excluding diagnostic surgery) within 4 weeks prior to the start of the study;<br>• Subjects with a history of substance abuse and are unable to quit or with a history of mental disorders;<br>• With a history of other malignant tumors in the past 5 years, except for cured skin basal cell carcinoma and cervical carcinoma in situ;<br>• Other severe, acute or chronic diseases, or psychiatric disorders or laboratory abnormalities that the investigator judges may increase the risk of participating in the study or may interfere with the interpretation of the findings.
**Investigational Products, Doses and Administrations**	The recombinant humanized anti-PD-1 monoclonal antibody is provided by the sponsor, specification: 240mg/6ml/bottle. The drug will be diluted with saline to complete the administration by intravenous drip for one hour.<br>Axitinib tablets are provided by the sponsor. Specifications: 5mg/28 tablets/box. According to the clinical research and the listed drug instructions of axitinib, the initial dosage of axitinib in the combination study is 5 mg bid, administered orally.<br>According to the clinical PK parameter analysis in the phase I clinical trial of JS001 by Peking University Cancer Hospital and Sun Yat-sen University Cancer Hospital, the drug basically reached the peak concentration level after the end of administration, and the half-life was about 5-12 days. At a dosing frequency of once every 2 weeks, JS001 trough blood concentration reached a steady state after 3 consecutive administration. The steady-state trough concentrations were about 1.5 μg/ml, 8 μg/ml, 25 μg/ml and 85 μg/ml at the dose of 0.3 mg/kg, 1 mg/kg, 3 mg/kg and 10 mg/kg respectively. A dose of 3 mg/kg Q2W achieved a desired peripheral blood concentration. At the same time, according to the PD-1 receptor occupancy (RO) analysis of the peripheral blood T lymphocytes in the phase I clinical trial of JS001 by Peking University Cancer Hospital and Sun Yat-sen University Cancer Hospital, all test dose groups, including 0.3, 1.0, 3.0, 10.0 mg/kg, and 240 mg fixed-dose every 2 weeks, maintained an average receptor occupancy rate of more than 80%.<br>Based on the PK/PD results of the JS001 Phase I clinical studies, as well as observed clinical efficacy in the 1 mg/kg and 3 mg/kg dose groups, the

	recommended initial dose of JS001 combined with axitinib is 1 mg/kg, with dose escalation to 3mg/kg.<br><br>Therefore, recombinant humanized anti-PD-1 monoclonal antibody JS001 combined with axitinib will be administered incrementally, and only the dose of JS001 will be adjusted during dose escalation. The planned dose for JS001 is 1 mg/kg and 3 mg/kg. The dose of axitinib will remain unchanged. Therefore, there will be four planned groups of advanced RCC and melanoma in two dose levels, 3-6 cases in each group:<br><br>Advanced RCC: 1.JS001 1 mg/kg Q2W + axitinib 5mg Bid<br>2.JS001 3 mg/kg Q2W + axitinib 5mg Bid<br><br>Advanced melanoma: 1.JS001 1 mg/kg Q2W + axitinib 5mg Bid<br>2.JS001 3 mg/kg Q2W + axitinib 5mg Bid<br><br>For each dose group, JS001 will be administered on the first day, and axitinib will be administered orally on the second day.
**Definition of maximum tolerated dose (MTD)**	MTD is defined as following:<br><br>MTD is the dose level before DLT dose. If DLT does not occur during the trial and the recommended effective dose for subsequent studies has been determined, 3 mg/kg JS001 will be the MTD for this trial.
**Definition of dose-limiting toxicity (DLT)**	The following treatment-related toxicity occurring within 28 days after administration will be defined as DLT (according to CTCAE 4.0):<br><br>• On the day of treatment, with pretreatment with glucocorticoids, paracetamol, promethazine, etc., patients have ≥grade 3 treatment-related adverse reactions;<br>• Or on any day other than day of treatment, any ≥grade 3 treatment-related non-hematologic adverse reaction occurs;<br>• Or ≥grade 4 hematological adverse reactions;<br>• Grade 3 hypertension (systolic blood pressure ≥160 mmHg and/or diastolic blood pressure ≥100 mmHg) which cannot be controlled to grade 2 (systolic blood pressure <160 mmHg and/or diastolic blood pressure <100 mmHg) or below within 7 days ;<br>• ≥Grade 3 diarrhea and gastrointestinal adverse reactions under supportive therapy.
**Concomitant drug/treatment**	During the study, investigators are permitted to provide supportive treatments to subjects according to clinical needs as described below. Any anti-tumor treatments undefined by the study protocol, such as surgery and radiotherapy are prohibited,

	excluding life-saving procedures during emergencies.<br><br>**Permitted concomitant drugs**<br><br>All concomitant medications need be recorded on the Case Report Form (CRF) during the study period, including all prescription, over-the-counter (OTC), and intravenous medications. If a change has occurred during the test, the dose, frequency, route of administration and date of the drug need to be recorded in the CRF.<br><br>**Prohibited concomitant drugs**<br><br>During the screening and treating periods, subjects are forbidden to receive:<br><br>• Systemic anti-tumor chemotherapy or biological therapy;<br><br>• Immunotherapy other than JS001;<br><br>• Investigational drug other than Axitinib;<br><br>• Radiotherapy (excluding local radiation therapy treating bone metastatic lesions);<br><br>• Vaccination within 4 weeks before/during administration, including but not limited to the vaccines for measles, epidemic parotitis, rubella, varicella, yellow fever, rabies, BCG and typhoid(oral);<br><br>• Any glucocorticoid other than treatment for adverse events due to immunotherapy (remarks: physiological doses of steroids may be allowed after communication with the sponsor);<br><br>• Inhibitors or inducers of CYP3A4/5;<br><br>• In general, traditional Chinese medicine (TCM) is not recommended during the study, and any use of TCM requires consultation with the sponsor.
**Study Visit**	Screen/baseline period (day -14~day 0)<br><br>Pre-enrollment screening and evaluation will be performed within 14 days prior to the initial dosing of the study drug when the signed ethics committee (IRB) approved informed consent for each subject is obtained. Screening will include complete medical history, physical examination, height, weight, vital signs (blood pressure and heart rate), ECG, ECOG scores, combined medication and clinical laboratory tests, including urine routine, blood routine (blood cell count), blood biochemistry Examination, coagulation (PT and PTT), thyroid function, lymphocyte subsets, HIV, HBV and HCV screening, pregnancy test (only in women of childbearing age). An imaging examination of the clinical staging of the tumor must be completed within 4 weeks (8 weeks of brain MRI) prior to the study drug administration. Pathological specimen tissue and blood samples will be collected for tumor marker analysis.<br><br>Treatment period

	Subjects will be administered at the indicated dose levels. Multi-dosing studies will be performed every 2 weeks, and there are four consecutive administrations per each dosing cycle. Patients who don't develop DLT during the observation period, if the investigator evaluates that they may continue to benefit, may continue to receive treatment until disease progression, intolerable toxicity or voluntary withdrawal.<br><br>JS001 will be administered on day 1, and blood will be collected before and after intravenous administration for PK analysis. The PK sampling time might be adjusted based on the results of previous dose group. On the second day, axitinib will be administered orally 5 mg bid.<br><br>On each visit, the combined medication and clinical symptoms of the subjects will be recorded, including any adverse events. All subjects entering the extended observation phase will be evaluated for clinical response every 8 weeks according to RECIST version 1.1 and irRC criteria until the end of the study.<br><br>Subjects with disease progression (PD) will discontinue from the study. In addition, subjects will discontinue from the study at any time if a DLT or disease progression occurs.<br><br><u>Post-treatment visit</u><br><br>About 4 weeks after the completion of the last treatment, the subjects will return to the research center for clinical evaluation, including a complete physical examination (including ECOG scores), vital signs, collection of adverse events and combined medication information, weight and laboratory evaluation. At the end of study, laboratory tests (blood routine, urine routine, blood biochemical tests, coagulation function, thyroid function, lymphocyte subsets), pregnancy tests (only for women of childbearing age), PK blood tests, and tumor marker tests will be included.<br><br>Patients who withdraw from the trial will be tracked for 30 consecutive days after the last administration or until the patients receive another anti-cancer treatment, whichever occurs first, to follow the outcome of any safety event associated with the study drug.
**Research assessment**	**Safety:**<br><br>If applicable, patient safety such as adverse events, physical exams, electrocardiograms (ECGs), vital signs, clinical laboratory assessments, medical history, and past/concomitant medications will be assessed throughout the trial. The safety parameters will be evaluated with reference to NCI CTCAE version 4.0.<br><br>**Pharmacokinetics:**<br><br>Blood samples will be taken for PK evaluation during the dose escalation phase and the expansion phase.<br><br>**Dose escalation phase:**

JS001 (V1.2/April 14, 2017) Protocol Number：HMO-JS001-Ib-CRP-01

	Continuous plasma sample is taken prior to each dose of JS001 for determination of plasma concentration-time profiles. All patients will receive a concentration sample before each dose in a subsequent cycle.<br><br>**Expansion phase:**<br><br>Patients' peripheral blood samples (about 8 ml) will be taken before the treatment of JS001. After the last drug administration, peripheral blood specimen (about 8ml) will be collected.<br><br>**Efficacy:**<br><br>The clinical efficacy will be evaluated by CT or MRI throughout the trial every 8 weeks after administration, according to the irRC and RECIST 1.1 criteria. After the disease progression or discontinuation from the study, survival follow-up will be conducted by phone every 3 months.<br><br>**PD-1 receptor occupancy：**<br><br>The PD-1 receptor occupancy in the blood will be measured to determine the inhibitory effect of the drug on the PD-1 target.
**Statistical analysis**	**Safety:**<br><br>All patients who enroll in the trial and receive at least one dose of study drug will be included in the safety analysis set. All patients will be evaluated for DLT events. AE frequency, classified by severity, and relationship to study drug will be analyzed. Descriptive statistical analysis will be used to report laboratory variables and vital signs. All adverse events and laboratory abnormal values will be evaluated with reference to CTCAE Version 4.0.<br><br>**Pharmacokinetic (PK) parameters:**<br><br>Serum drug concentration over time for all subjects will be summarized. Non-compartmental model analysis will be used. The drug concentration of JS001, peak concentration (Cmax), blood area-time curve area (AUC), peak time (Tmax), serum terminal elimination rate constant (λz) , terminal half-life (t1/2), apparent volume of distribution (Vd), volume at steady state (Vdss), and systemic clearance (CL) will be determined for each subject and the linear relationship of PK/PD parameters to dose will be determined.<br><br>**Efficacy:**<br><br>All patients who receive at least one dose of study drug will be included in the efficacy analysis. The objective response rate, duration of response (DOR), PFS, and OS will be collected and calculated per the irRC and RECIST 1.1 criteria. Descriptive results will be given by dose groups.

List of Abbreviations and Relevant Terms

Initials/Abbreviations	Terms
ADA	Anti-drug Antibody
ADR	Adverse Drug Reaction
AE	Adverse Event
ALP	Alkaline Phosphatase
ALT(SGPT)	Alanine Aminotransferase
aPTT	active partial thrombin time
AST(SGOT)	Aspartate Aminotransferase
ANC	Absolute Neutrophils Count
AUC	Area under the Serum Drug Concentration-Time Curve
BUN	Blood Urea Nitrogen
CBC	Complete Blood Count
CFDA	China Food and Drug Administration
CHO	Chinese Hamster Ovary
Cl^-	Chlorine
CRP	C-Reactive Protein
CT	Computed Tomography
CTCAE	Common Terminology Criteria for Adverse Events
DLT	Dose-Limiting Toxicity
DSMB	Data Safety Monitoring Board
EC	Ethics Committee
ECG	Electrocardiogram
ECOG	Eastern Cooperative Oncology Group
eCRF	Electronic Case Report Form
EDC	Electronic Data Capture
FAS	Full Analysis Set

GCP	Good Clinical Practices
GLP	Good Laboratory Practices
HCC	Hepatocellular Carcinoma
HIV	Human Immunodeficiency Virus
IC	Informed Consent
ICF	Informed Consent Form
ICH	International Conference on Harmonization
INR	International Normalized Ratio
IRB	Institutional Review Board
IV	Intravenous
K^+	Potassium
Kd	Dissociation Constant
kg	Kilogram
LDH	Lactic Dehydrogenase
Mab	Monoclonal Antibody
MedDRA	Medical Dictionary for Regulatory Activities
mg	Milligram
mL	Milliliter
MRI	Magnetic Resonance Imaging
MTD	Maximum Tolerated Dose
Na^+	Sodium
NK	Natural Killer Cells
PBMC	Peripheral Blood Mononuclear Cell
PBSCT	Peripheral Blood Stem Cell Transplantation
PD	Progressive Disease
PK	Pharmacokinetics
PS	Performance Status
PT	Prothrombin time
PLT	Platelet
RBC	Red Blood Cell
RECIST	Response Evaluation Criteria in Solid Tumors
RO	Receptor Occupancy
SAE	Serious Adverse Event
SAP	Statistical Analysis Plan
SOP	Standard Operating Procedure

$t_{1/2}$	Half-Life
TCR	Tissue Cross Reactivity
WBC	White Blood Cell

Table of Contents

Investigator’s Statement........2
Sponsor’s Statement........3
Organization of the clinical trial........4
Protocol Synopsis........5
Protocol........错误!未定义书签。
1.Background........21
1.1 Introduction........21
1.2 JS001 relevant information........22
1.2.1 Preliminary summary of phase I study in Beijing cancer hospital（As of November 11, 2016）........22
1.2.1.1 Preliminary analysis of the basic situation........23
1.2.1.2 Preliminary analysis of safety data:........24
1.2.1.1 Preliminary analysis of effectiveness........25
1.2.1.2 Preliminary analysis of pharmacokinetics........25
1.2.1.3 Preliminary analysis of receptor occupancy (RO)........25
1.2.1.4 Preliminary summary of the phase I study........25
1.2.2 Preliminary summary of phase I study in Sun Yat-sen University Cancer Hospital........26
2. Study Objectives........31
2.1 Primary Objective........31
2.2 Secondary Objectives........31
4. Study Endpoints........31
4.1 Primary Endpoint........31
4.2 Secondary Endpoint........31
4.3 Exploratory Endpoints........32
5. Study Design........32
5.1 Overall design........32
5.1.1 Dose escalation phase........32
5.1.2 Dose expansion phase........33
5.1.3 Definition of maximum tolerated dose (MTD)........34
5.1.4 Definition of dose-limiting toxicity (DLT)........34
5.2 Study Procedures........34
5.2.1 Patient Screening........34
5.2.2 Patient enrolled........35
5.2.3 Patient dose distribution........35
5.2.4 Duration of the test........35
6. Study population........35
6.1 Inclusion Criteria:........35
6.2 Exclusion Criteria........37
6.3 Patient withdrawal criteria........38
6.4 Withdraw from the trial........38
7. Investigational Products........39
7.1 Investigational Products........39

7.2 Package and Tag....39
7.3 Management of Investigational Products....40
7.3.1 Reception and Storage of JS001....40
7.3.2 Disposal of JS001....40
7.4 Administration....40
7.4.1 Dose determination basis....40
7.5 Drug preparation and record of JS001....41
7.6 Notice of JS001....42
7.7 Concomitant drug/treatment....43
7.8 Drug adherence....43
7.9 Standard for dose adjustment of the combination administration....44
8 Research visit and evaluation....44
8.1 Informed consent....44
8.2 Medical history....45
8.3 Demographics....45
8.4 Medical examination....45
8.5 ECOG score....45
8.6 ECG....45
8.7 Vital signs....45
8.8 Laboratory inspection....46
8.9 Tumor evaluation....46
8.10 Immunogenicity test....47
8.11 Pharmacodynamic test....47
8.12 Tumor sample collection....47
8.13 Combination therapy....48
8.14 Evaluation of adverse events....48
9. Safety assessment....48
9.1 Parameters of Safety Assessment....48
9.2 Adverse Events (AE)....49
9.2.1 Definition of Adverse Events (AE)....49
9.2.2 Laboratory abnormal results....49
9.2.3 Serious adverse events (SAE)....49
9.2.4 Causality Assessment....50
9.2.5 Adverse Drug Reactions (ADR)....51
9.2.6 Assessment of severity....51
9.2.7 Assessment of severity....52
9.2.8 Measures taken for the treatment of patients....52
9.2.9 Measures taken to test drugs....52
9.2.10 final result....52
9.3Recording and reporting of adverse events....52
9.3.1 Recording of adverse events....52
9.3.2 Report of serious adverse events....53
9.4 Pregnancy report and follow-up....53
9.5 Data and Safety Monitoring Board (DSMB)....53

10. Pharmacokinetic evaluation 54
10.1 Evaluation parameters 54
10.2 Assessment time point 54
10.2.1Blood collection of recombinant humanized anti-PD-1 monoclonal antibody injection 55
11. Effectiveness evaluation 56
11.1 Tumor evluation 56
11.2 ECOG PS 56
12. Statistic analysis 56
12.1 Data sets 56
12.1.1 Safety 56
12.1.2 Pharmacokinetics 57
12.1.3 Full Analysis Set (FAS) 57
12.2 Processing of missing data 57
12.3 Analysis method 57
12.3.1 Demographic and baseline disease characteristics 57
12.3.2 Safety 57
12.3.3 Pharmacokinetics 58
12.3.4 Effectiveness 58
12.3.4.1 Antineoplastic activity 58
12.3.4.2 ECOG PS 58
12.4 Estimated number of patients 58
12.5 Mid-term analysis 59
13. Test Quality Control 59
14. Ethnical 59
14.1 Informed consent 59
14.2 Signature of informed consent 59
14.3 Amendment of informed consent 60
14.4 Institutional Ethics Committee (EC) 60
14.5 Protection of patients’rights and interests 60
14.6 Compensate 60
15. Data Processing and Record Preservation 61
15.1 Case report form 61
15.2 Record keeping 61
16 Source Data Management 62
16.1 Types of raw data 62
16.2 Direct access to raw data/records 62
17. Public Publication Policy 62
18. Revision of test plan and deviation of test plan 63
18.1 Revision of test plan 63
18.2 Test scheme deviation 63
19. Test interruption and termination 64
19.1 The sponsor asked for interruption of the test 64
19.2 End of test 64

References........65
Appendix1：Study Flow Chart........69
Appendix2: Eastern Cooperative Oncology Group (ECOG) Performance Status........74
Appendix3: NCI Common Terminology Criteria for Adverse Events version 4.0（NCI CTCAEversion 4.0）........75
Appendix4: Response Evaluation Criteria in Solid Tumors (RECIST1.1)........76
Appendix5: Immune-Related Response Criteria (irRC)........94
Appendix6: Content of Informed Consent........96
Appendix7: Statute of the Data and Safety Monitoring Board........98

1.Background

1.1 Introduction

Malignant tumors are major diseases that plague human health. The main treatment means are surgery, radiotherapy and chemotherapy. From the 1970s to the 1980s, biological immunotherapy methods such as antibody therapy and cytokines have been continuously explored. In recent years, the PD-1/L1 pathway-mediated immune regulation mechanism is the breakthrough of tumor immunotherapy. Tumor cells can escape the immune surveillance of activated T cells through the PD-1/L1 pathway. PD-1 is expressed on the surface of activated T cells. Under normal conditions, its normal function is to down-regulate unwanted or excessive immune responses, including autoimmune responses. PD-1, a member of the immunoglobulin (Ig) superfamily, has been shown to negatively regulate antigen receptor signaling when PD-1 binds to its ligand (PD-L1 and/or PD-L2) [1-2]. Although normal organs can only express a small amount (if any) of PD-L1, studies have confirmed that various tumor cells can express a large amount of such T cell inhibitors. It has been found that high expression of PD-L1 in tumor cells (lower levels of PD-L2) is associated with poor prognosis and survival of various tumors, including renal cell carcinoma (RCC), pancreatic cancer, hepatocellular carcinoma, ovarian cancer, and Non-small cell lung cancer (NSCLC). [3-6]. In addition, the study also found that PD-1 can regulate the expansion of tumor-specific T cells in melanoma patients [7]. This suggests that the PD-1/PD-L1 pathway plays an important role in tumor invasion and is therefore a high-profile therapeutic intervention target [8].

Ipilimumab (trade name: Yervoy), a drug targeting CTLA-4, developed by Squibb, is the first immunological checkpoint antibody against the world, and its indication is advanced malignant melanoma [9-10]. In 2014, the PD-1 inhibitor Nivolumab (trade name: Opdivo) developed jointly by Bristol-Myers Squibb and Japan Ono Pharmaceuticals was first marketed in Japan for the treatment of advanced melanoma [11] and was subsequently approved for the treatment of advanced RCC [12]. Merck's PD-1 inhibitor, Pembrolizumab (trade name: Keytruda), is also marketed in the United States for advanced melanoma treatment [13-14]. Compared with traditional chemical therapy and targeted therapeutic drugs, these immune checkpoint antibody drugs have the feature of broad spectrum, specificity, low toxicity, long-lasting efficacy, etc., these indications of drugs soon spread to lung cancer, lymphoma and other fields. The anti-PD-L1 antibody Atezolizumab (trade name: Tecentriq), developed by Roche in the United States, was successful in advanced bladder urothelium and non-small cell lung cancer, respectively [15-16].

Immunological checkpoint molecules play a key role in the process of tumor evasion of immune surveillance. PD-1 can inhibit T cell activation by binding to its ligand PD-L1 or PD-L2. When CTLA-4 binds to CD80 and CD86 on the surface of

antigen-presenting cells (APC), T cells can also be inactivated. The above mechanism can be blocked by competitive inhibition of PD-1 or CTLA-4 by antibodies, thereby enhancing the killing activity of T cells. The results of the CheckMate-067 showed that the combination of Nivolumab and Ipilimumab increased the ORR to 57%-61% and the mPFS to 11.5 months. This clinical study validated the significant efficacy of combined immunotherapy [18]. Therefore, on October 1, 2015, FDA approved the combination of Nivolumab and Ipilimumab for the treatment of unresectable or metastatic malignant melanoma. In addition to melanoma, PD-1 monoclonal antibody combined with CTLA-4 monoclonal antibody is also undergoing clinical research in solid tumors such as non-small cell lung cancer, and the preliminary results are encouraging [19-22].

In addition to the combination of immune checkpoint inhibitors, research on immunological checkpoint inhibitors combined with vaccines and targeted drugs is also underway. The results of various studies are different. In this aspect, advanced RCC and melanoma treatment is ahead of other type of tumors. A preliminary study of PD-L1 monoclonal antibody Atezolizumab combined with MEK inhibitor Cobimetinib in the treatment of BRAF wild-type metastatic melanoma was reported at the 2016 melanoma SMR annual meeting. The results showed that 75% of tumors decreased with different degree and duration of response reached 14.9 months. In the field of advanced renal cancer, which is dominated by anti-angiogenic tyrosine kinase inhibitors (TKI), the 2016 ESMO conference reported two PD-1 antibodies or PD-L1 monoclonal antibody in combination with axitinib for the treatment of advanced RCC. The phase I clinical study of cancer has a very significant efficacy. One was preliminary data of a Phase Ib trial of Pembrolizumab combined with axitinib: 71.2% of the 52 patients achieved objective response, 94% of the patients had tumor shrinkage; Another PD-L1 monoclonal antibody Avelumab combined with axitinib In the Ib phase dose study of patients with newly diagnosed advanced renal cell carcinoma, all the 6 patients were initially reported to have partial response, and 5 patients (83.3%) when the analysis was reported. Therefore, the treatment of immunotherapy combined with targeted drugs deserves further expectation.

1.2 Property of JS001

JS001 is a neutralizing blocking antibody targeting on human PD-1. It binds to PD-1 with high affinity and selectively blocks the binding of PD-1 with its ligands PD-L1 and PD-L2 to activate T-lymphocytes and enhance the proliferation of T-lymphocytes and secretion of cytokines, especially IFN-γ.

The preclinical pharmacodynamic trial has proved that, in animal models with graft-versus-host disease (GVHD) induced by adoptive transfer of human PBMC, JS001 could significantly stimulate the proliferation of CD4+ and CD8+ T cells to promote the activation of human effector/memory T cells. Meanwhile, the study in animal

models implanted with 624 MEL human melanomas has proved that, JS001 in combination with cytotoxic T lymphocytes (CTL) could eliminate the immunosuppressive effects and enhance the killing effect of CTL on tumor cells, thus achieving the expected good therapeutic effect.

The difference in CDR sequences and structure of the antigen-binding site between JS001, Nivolumab and Pembrolizumab mainly manifests in 6 CDR sequences, which determines the various physical, chemical and biological characteristics of the three drugs. Based on the affinity test results using SPR or ELISA methods, JS001 has higher affinity than Pembrolizumab and Nivolumab (See Table 1).

Table 1 Comparison of Structures and Physical and Chemical Properties between JS001 and Drugs with the Same Target

Specimen	Subtype of Antibody		Affinity (SPR)	Binding EC50 (Elisa)	Source
	Heavy Chain	Light Chain			
JS001	IgG4	Kappa	0.92 nM	64 pM	Humanized
Pembrolizumab	IgG4	Kappa	Not Reported	70 pM	Humanized
Nivolumab	IgG4	Kappa	2.64 nM	Not Reported	Fully Humanized

JS001 formulation is developed as injection, with the strength of 240mg/6mL/vial. The drug clinical trial on JS001 is approved by China Food and Drug Administration on December 27, 2015, with an approval number of 2015L05752.

1.2.1 Preliminary summary of JS001 phase I study in Beijing cancer hospital（As of November 03, 2016）

1.2.1.1 Preliminary analysis of enrollment

A phase I study (project number: HMO-JS001-I-CRP-01), in the Beijing Cancer Hospital, aimed at investigating the tolerability, safety and dose-limiting toxicity (DLT) of one dose and multiple intravenous infusions of JS001s in patients with malignant melanoma and advanced urological tumors. As of November 3, 2016, a total of 28 subjects were enrolled in the dose-escalation study according to the program. It is currently in the dose expansion phase. The trial information is summarized as below:

Table 2. List of subjects in HMO-JS001-I-CRP-01 clinical trials

Item	Cases	Notes
Number of enrolled	28	19 Melanoma, 5 RCC, 3 bladder cancer, and 1 ureteral carcinoma
Dose cohorts	1mg/kg dose escalation group	3

	3mg/kg dose escalation group	4
	10mg/kg dose escalation group	3
	1mg/kg dose expansion group	11
	3mg/kg dose expansion group	7
SAE	2	1mg/kg group: 01K1013<br>3mg/kg group: 01006

1.2.1.2 Preliminary analysis of safety data:

As of November 3, 2016, 28 subjects who were enrolled did not have an infusion reaction. Common AE included rash, fatigue, decreased white blood cells, decreased thyroid stimulating hormone, anemia, and elevated AST. All the AEs were grade 1-2, without grade 3-4. SAE occurred in 2 cases. Preliminary analysis found no dose-effect relationship between the drug-related toxicity and dose.

Table3. List of AE of each cohort in HMO-JS001-I-CRP-01 clinical trial

Type of AEs	Incidence of 1mg/kg dose group（N=14）	Incidence of 3mg/kg dose group（N=11）	Incidence of 10mg/kg dose group（N=3）	Total percentage（N=28）
SAE	1 case of fever leads to prolonged hospital stay, not related to the study drug	1 case of disease progression (intestinal obstruction) leads to death, not related to the study drug		
Gastrointestinal reaction	4（29%）	0	0	4（14%）
Rash	4（29%）	2（18%）	0	6（21%）
Hypothyroidism	2（14%）	2（18%）	0	4（14%）
Fever	2（14%）	1（9%）	1（33%）	4（14%）
Fatigue	2（14%）	1（9%）	0	3（11%）
Hepatotoxicity	2（14%）	0	0	2（7%）

anemia	2（14%）	0	0	2（7%）

1.2.1.1 Preliminary analysis of clinical efficacy

As of November 3, 2016, 17 patients had been evaluated for clinical response after treatment. 10 mg/kg dose escalation cohort and the 3 mg/kg dose expansion cohort had not yet reached the evaluation time. The initial clinical response was as follows:

Table 4. Clinical efficacy of each cohort in HMO-JS001-I-CRP-01 clinical trial

Dose group	CR	PR	SD	PD	ORR（CR+PR）
1 mg/kg（N=13）	0	1	6	6	8%
3mg/kg（N=4）	1	1	0	2	50%
10 mg/kg	Not yet evaluated				

1.2.1.2 Preliminary analysis of pharmacokinetics

According to the clinical PK analysis of Phase I JS001 studies in Beijing Cancer Hospital and Sun Yat-sen University Cancer Hospital, the drug basically reached the peak concentration level at the end of infusion, and the half-life was about 5-12 days. At a dosing frequency of once every 2 weeks, JS001 trough blood concentration reached a steady state after 3 consecutive administrations. The steady-state trough concentrations were about 1.5 µg/ml, 8 µg/ml, 25 µg/ml and 85 µg/ml at the dose of 0.3 mg/kg, 1 mg/kg, 3 mg/kg and 10 mg/kg respectively. A dose of 3 mg/kg Q2W achieved a desired peripheral blood concentration.

Based on the PK/PD results of the JS001 Phase I clinical studies, as well as observed clinical efficacy in the 1 mg/kg and 3 mg/kg dose groups, the recommended initial dose of JS001 combined with axitinib is 1 mg/kg, with dose escalation to 3mg/kg.

1.2.1.3 Preliminary analysis of receptor occupancy (RO)

At the same time, according to the PD-1 receptor occupancy (RO) analysis of the peripheral blood T lymphocytes in the phase I clinical trial of JS001 by Peking University Cancer Hospital and Sun Yat-sen University Cancer Hospital, all test dose groups, including 0.3, 1.0, 3.0, 10.0 mg/kg, and 240 mg fixed-dose every 2 weeks, maintained an average receptor occupancy rate of more than 80%.

1.2.1.4 Preliminary summary of the phase I study HMO-JS001-I-CRP-01

As the data cut-off date, none of 28 patients had a DLT event nor an infusion reaction. Based on the PK/PD results of the JS001 Phase I clinical studies, as well as observed clinical efficacy in the 1 mg/kg and 3 mg/kg dose groups, the recommended phase II dose was 3mg/kg Q2W.

1.2.2 Preliminary summary of phase I study in Sun Yat-sen University Cancer Hospital

The JS001-I-CRP-1.0 was a Phase I, dose escalation study conducted at Sun Yat-sen University Cancer Hospital in a variety of advanced solid tumors refractory to standard treatment. The dose escalation followed the 3+3 principle. The dose cohorts included 0.3 mg/kg, 1 mg/kg, 3 mg/kg, and 10 mg/kg JS001 every 2 weeks. The first subject was enrolled on March 7, 2016. As of July 28, 2016, 19 subjects were enrolled in the study. The subjects enrolled in each dose group were listed in Table 5:

Table 5. List of subjects in JS001-I-CRP-1.0

Dose group (mg/kg)	No.	Gende r	Primary Tumor	Time to response (week)	Best tumor response	Duration of response(week)
0.3	0001	Male	esophageal cancer with bilateral lung, pleural, lymph node metastasis	none	PD	none
0.3	0002	Male	laryngeal carcinoma with Lung and multiple lymphatic metastasis	The 6th week	PR	7 weeks
0.3	0003	Male	poorly differentiated gastric adenocarcinoma with liver metastasis	none	PD	none
1	0004	Male	pancreatic cancer with multiple metastases in liver, lung and abdominal cavity	none	PD	none
1	0005	Male	esophageal squamous cell carcinoma, with liver and lung metastases	none	PD	none
1	0006	Male	gastric adenocarcinoma with liver and abdominal lymph node metastasis	none	PD	none
1	0007	Male	nasopharyngeal carcinoma with liver metastasis	none	PD	none
3	0008	Male	pancreatic adenocarcinoma with multiple peritoneal metastases, right abdominal wall metastasis	none	PD	none
3	0009	Male	esophageal cancer with lung	The 6th	PR	none

			metastasis	week		
3	0010	Male	nasopharyngeal carcinoma with liver metastasis	none	PD	none
10	0011	Female	gastric cancer with multiple metastases	none	PD	none
3 Expansion	0012	Male	gastric cancer with liver metastasis	none	PD	none
10	0013	Male	Nasopharyngeal carcinoma with multiple metastases of liver, lung and bone	none	PD	none
10	0014	Male	Nasopharyngeal carcinoma with liver and bone metastasis	none	PD	none
3 Expansion	0015	Male	hepatobiliary cell carcinoma with lung metastasis	none	PD	none
3 Expansion	0016	Female	nasopharyngeal carcinoma, multiple lung, bone, and lymph nodes metastasize,	none	PD	none
10 Expansion	0017	Female	Melanoma with multiple metastases (lung, bilateral pleura, lymph nodes)	none	PD	none
10 Expansion	0018	Female	Gastric adenocarcinoma with multiple liver metastases	none	PD	none
10 Expansion	0019	Female	metastatic melanoma	The 6th week	PR	none

As of July 28, 2016, no subjects had any protocol-defined DLT events during the scheduled DLT observation period (28 days after the first dose). The most common AEs that occurred so far (incidence rates over 10%) are listed in Table 6, with most of the AEs at Grade 1-2 according to CTCAE 4.0.

Table 6. List of common AEs

Any events	JS001 dose group			
	0.3mg/kg N=3	1mg/kg N=4	3mg/kg N=6	10mg/kg N=6
Total				
Mental, blood, neuromuscular system events				
Insomnia	0	1（25%）	1（33%）	0
Anemia	1（33%）	2（50%）	1（17%）	0
Gastrointestinal disease				
Nausea	1（33%）	1（25%）	1（17%）	1（17%）
Vomiting	1（33%）	1（25%）	0	0

Any events	JS001 dose group			
	0.3mg/kg N=3	1mg/kg N=4	3mg/kg N=6	10mg/kg N=6
Constipation	1（33%）	3（75%）	3（50%）	0
Diarrhea	0	0	1（17%）	0
Stomachache	1（33%）	1（25%）	1（17%）	0
Abdominal distension	0	1（25%）	0	1（17%）
Anorexia	0	1（25%）	2（33%）	0
Peptic ulcer	0	0	0	1（17%）
Hepatic and gall diseases				
Ascites	0	0	1（17%）	0
Skin and systemic symptoms and local injection response events				
Rash	2（67%）	1（25%）	1（17%）	0
Pruritus	1	1（25%）	1（17%）	0
Fatigue	2（67%）	1（25%）	5（83%）	1
Fever	0	1（25%）	1（17%）	1（17%）
Respiratory, chest and mediastinal organ events				
Cough	1（33%）	0	1	2（34%）
Metabolic and endocrine events				
FT3 increase	0	1（25%）	0	0
FT3 decrease	1（33%）	1（25%）	0	0
FT4 increase	0	1（25%）	0	0
FT4 decrease	1（33%）	0	0	1（17%）
Hyperkalemia	0	1（25%）	0	0
Hypokalemia	0	1（25%）	1（17%）	0
Hyponatremia	1（33%）	2（50%）	1（17%）	0
Hypocalcemia	0	0	1（17%）	0
Hypoalbuminemia	0	1（25%）	1（17%）	0
Kidney and urinary system diseases				
Proteinuria	0	3（75%）	2（33%））	1
Hematuria	0	0	1（17%）	0
Medical examination				
Leukopenia	0	0	0	1（17%）
Neutropenia	0	0	0	1（17%）

Any events	JS001 dose group			
	0.3mg/kg N=3	1mg/kg N=4	3mg/kg N=6	10mg/kg N=6
ALP elevation	2（66%）)	1（25%）	0	1（17%）
γ glutamine transferase elevation	1（33%）	3（75%）	1（17%）	3（50%）)
Alanine aminotransferase elevation	1（33%）	2（50%）	0	0
Aspartate aminotransferase elevation	1	4（100%）	2（33%）	2（33%）
Total bilirubin elevation	0	3（75%）	0	0
Direct bilirubin elevation	0	3（75%）	0	0
Indirect bilirubin elevation	0	1（25%）	0	0
Hyperglycemia	1（33%）	1（25%）	1（17%）	0
Hyperuricemia	0	1（25%）	0	0
Hypercholesterolemia	2（66%）	2（50%）	0	1（17%）
Hypertriglyceridemia	0	3（75%）	0	0
ECG QT interval extension	1（33%）	0	0	1（17%）

As of July 28, 2016, the incidence of SAE is listed in table 7.

Table 7. List of SAE

Event name	CTCAE Grade	JS001 dose group	No. of the subjects	Gender	Relation with research drugs
Hospitalization for abdominal pain	Grade 3	0.3mg	0003	Male	Unrelated
Hospitalization for liver damage	Grade 3	1mg	0005	Male	Unrelated
Hospitalization for liver damage	Grade 3	1mg	0007	Male	Unrelated

Overall, the incidence rate of SAE was 15.8% (3/19). The three SAE cases were listed in detail below.

0003 patient with poorly differentiated gastric adenocarcinoma and liver metastasis, received first dose of JS001 on March 29, 2016 and received the last dose

on April 26, 2016. On May 9, 2016, the subject was admitted to the hospital due to hepatic pain and CT showed that liver metastases increased both in size and number. The investigator judged that the SAE was not related to the study drug.

0005 patient was diagnosed with esophageal squamous carcinoma with liver and lung metastases. He received the first dose of JS001 on April 15, 2016 and received the last dose on May 13, 2016. On May 25, the patient showed grade 3 TBIL elevation, grade 4 DBIL elevation, grade 3 AST and grade 2 ALT elevation. On May 26, CT examination showed multiple liver metastases, which increased both in size and number, suggesting disease progression. The investigator judged that the SAE was not related to the study drug.

0007 patient with nasopharyngeal carcinoma and liver metastasis received the last dose of JS001 on May 20, 2016. On June 2, the patient showed grade 2 TBIL elevation, grade 3 DBIL elevation, grade 1 AST and grade 3 ALT elevation. On June 2, CT showed diffuse liver metastases, which increased significantly, suggesting disease progression. The patient was hospitalized on June 2 and the investigator judged that the SAE was not related to the study drug.

Based on the above results, JS001 was well tolerated in Phase I dose escalation studies, and clinical responses were observed in advanced RCC and melanoma patients.

1.3 Axitinib

Axitinib (trade name Inlyta) is a new generation of VEGFR multi-target tyrosine kinase inhibitors. The main targets of axitinib are VEGFR-1, VEGFR-2, VEGFR-3, PDGFR-β and c -KIT. The most common (≥20%) adverse reactions are diarrhea, hypertension, fatigue, loss of appetite, nausea, dysphonia, HFS, weight loss, vomiting, fatigue, and constipation. [23-24]。

The clinical data of axitinib in patients with advanced renal cell carcinoma who had disease progression after initial systemic therapy is based on a randomized controlled multicenter international phase III clinical trial which compare effectiveness of axitinib versus sorafenib in advanced renal cell carcinoma after cytokine or TKI. [23-24] The trial (AXIS study) showed that axitinib treatment significantly prolonged the median PFS to 6.7 months, with an ORR of 19% and a mOS of 20.1 months. The stratified analysis showed that for the patients who received sunitinib in the previous line, axitinib group significantly increased mPFS compared with the sorafenib group, which was 4.8 months and 3.4 months, respectively. Domestic patients are based primarily on a registry clinical trial of axitinib in patients with metastatic renal cell carcinoma as second-line therapy in Asia [25]. Most of the patients in the trial are Chinese. The design of the trial is similar to AXIS studies, in which the mPFS of axitinib is 6.5 months, the ORR of axitinib is 23.7%. Subgroup analysis showed that mPFS for second-line axitinib in patients receiving previous sunitinib treatment was 4.7 months. Therefore, CFDA approved axitinib as the second-line treatment for advanced RCC

with the dosage of 5mg Bid.

The data for the treatment of advanced melanoma with axitinib is mainly from a multicenter phase II clinical study. A total of 32 patients with advanced melanoma who were treated no more than one line were enrolled and treated with axitinib (5 mg Bid). Result shows that ORR is 18.8%, median duration of response is 5.9 months, the PFS rate at the 6th month is 33.9%, and 1-year survival rate was 28.1% [26].

2. Study Objectives

2.1 Primary Objective

To evaluate the tolerability and safety of JS001 (a recombinant humanized anti-PD-1 monoclonal antibody) combined with axitinib in advanced renal cell carcinoma and advanced melanoma subjects naïve to systemic therapy or had limited prior systemic therapy. To evaluate dose-limiting toxicity (DLT), maximum tolerated dose (MTD) and recommended dose (RD) to provide the basis for the development of a dosing regimen for later stage of clinical trials.

2.2 Secondary Objectives

To evaluate the pharmacokinetics of JS001 combined with axitinib after administration

- To evaluate the anti-tumor activity of JS001 combined with axitinib after administration.
- To evaluate the effect on the PD-1 target, and the association between drug metabolism and drug inhibitory functions, by evaluating PD-1 receptor occupancy of T lymphocytes in blood samples after administration.
- To investigate the immunogenicity of JS001, observe the changes of anti-drug antibody production in vivo after administration, and provide reference for the safety and efficacy of the drugs.
- To provide reference for population selection by detecting the expression of PD-L1 in tumor tissue samples and conducting correlational analysis of PD-L1 expression with clinical efficacy.

4. Study Endpoints

4.1 Primary Endpoint

Safety: incidence and severity of adverse events, and clinically significant laboratory abnormalities, ECG and vital signs.

4.2 Secondary Endpoint

- Pharmacokinetic（PK）parameters; Single dose C_{max}, T_{max} and AUC_{0-T}, $AUC_{0-\infty}$, $t_{1/2}$, CL, V; C_{min} on steady state after multiple administrations, C_{av}, fluctuation factor (DF)， Vss, etc.

- Target-related pharmacodynamic indicators, such as detection of PD-1 receptor occupancy in the blood.
- Objective response rate (ORR) evaluated based on Response Evaluation Criteria in Solid Tumors (RECIST 1.1) and Immune-Related Response Criteria (irRC).
- Duration of response (DOR)
- Disease control rate (DCR)
- Time to response (TTR)
- Progression free survival (PFS)
- Overall survival (OS)

4.3 Exploratory Endpoints

- Correlation analysis of PD-L1 expression in tumor tissue with the anti-tumor activity

5. Study Design

5.1 Overall design

This study is the first human study of recombinant humanized anti-PD-1 monoclonal antibody (JS001) combined with axitinib. It's designed to be a single-center, open-label, Dose Escalation, Phase Ib clinical study. It intends to enroll advanced RCC and melanoma patients. The study is divided into two parts: the dose escalation phase and the expansion phase.

The purpose of the dose escalation phase is to evaluate the safety and tolerability of JS001 combined with axitinib, the pharmacokinetics of JS001 and dose-limiting toxicity (DLT), maximum tolerated dose (MTD)/recommended dose (RD) of the combined therapy. Up to 18-24 patients is planned to be enrolled in this phase of the study.

At the end of the dose escalation phase, additional RCC and melanoma subjects will be enrolled in the expansion phase. For efficacy evaluation in the expansion phase, there will be at least 10 subjects in the recommended dose group. We will further explore the pharmacokinetic characteristics, dose, efficacy and safety of JS001 after co-administration with axitinib.

5.1.1 Dose escalation phase

After determining the eligibility of the subjects, the subjects will be assigned to a certain dose group according to the order in which they are selected. Dose is escalated independently in each disease. If DLT occurs at the initial dose, the dose is decreased to 0.3 mg/kg. At least 3 subjects were enrolled in each group, and at least 6 subjects were enrolled at the MTD dose level. The maximum dose of JS001 is 3 mg/kg. After climbing to the maximum dose, the sponsor and the investigator will make a decision whether continue dose escalation or initiate an intermediate dose group based on

previous observations.

The dose escalation will be performed based on the 3+3 principle. Dose escalation is applied individually in each type of tumor. The DLT incidence is monitored in the first 28 days after treatment, and DLT safety evaluation is performed on the 29th day. The toxicity is graded using the NCI-CTCAE 4.0 standard. (See the DLT definition below for details). The dose escalation method is described as follows:

- If the first 3 subjects in the first dose level group (JS001 1 mg/kg Q2W + axitinib 5 mg Bid) do not develop DLT within the first 28 days of treatment, the dose escalation will continue, and 3 subjects will be enrolled to the second group (JS001 3 mg/kg Q2W + axitinib 5 mg Bid).
- If one of the 3 subjects has a DLT, 3 subjects should be added to this dose group. If there is no DLT in the 3 new subjects (only 1 of 6 cases), then enter the next higher dose group.
- If DLT occur in 2 or more of the first 6 subjects in the 1 mg/kg JS001 dose group, DLT has reached and no higher dose will be initiated, and the protocol will be reassessed.
- If the first 3 subjects with JS001 dose escalation to the 3 mg/kg group do not develop DLT or 5 of the first 6 subjects did not develop DLT, the dose escalation is completed and the 3 mg/kg Q2W + axitinib 5 mg Bid dose will be the recommended dose for the study.
- If 2 of the 6 subjects in the JS001 3 mg/kg group achieve DLT criteria, the axitinib 5mg Bid is reduced to 5 mg QD to continue the dose escalation evaluation. If the first 3 subjects or 5 of the first 6 subjects do not develop DLT, the dose escalation is completed and JS001 3 mg/kg Q2W + axitinib 5 mg QD will be the recommended dose for the study.
- If 2 of the 6 subjects in the JS001 3mg/kg Q2W + axitinib 5mg QD dose group meet the DLT criteria, JS001 1mg/kg Q2W + axitinib 5mg QD will be the recommended dose for the study.

5.1.2 Dose expansion phase

After determining the recommended dose, an expansion phase will be performed at this dose.

At the end of the DLT observation period, patients with no disease progression and no DLTs will be determined by the investigator whether they are eligible for continuing treatment. If permitted, they will continue to receive JS001 in combination with axitinib at the same dose level conducted during the dose escalation period. The subject will be treated until disease progression, intolerable toxicity, voluntary withdrawn, or the investigator assesses that the subject can no longer benefit from treatment.

5.1.3 Definition of maximum tolerated dose (MTD)

MTD is defined as following:

MTD is the dose level before DLT dose. If DLT does not occur during the trial and the recommended effective dose for subsequent studies has been determined, 3 mg/kg JS001 will be the MTD for this trial.

5.1.4 Definition of dose-limiting toxicity (DLT)

The following treatment-related toxicity occurring within 28 days after administration will be defined as DLT (according to CTCAE 4.0):

- On the day of treatment, with pretreatment with glucocorticoids, paracetamol, promethazine, etc., patients have ≥grade 3 treatment-related adverse reactions;
- Or on any day other than day of treatment, any ≥grade 3 treatment-related non-hematologic adverse reaction occurs;
- Or ≥grade 4 hematological adverse reactions;
- Grade 3 hypertension (systolic blood pressure ≥160 mmHg and/or diastolic blood pressure ≥100 mmHg) which cannot be controlled to grade 2 (systolic blood pressure <160 mmHg and/or diastolic blood pressure <100 mmHg) or below within 7 days ;

≥Grade 3 diarrhea and gastrointestinal adverse reactions under supportive therapy.

5.2 Study Procedures

5.2.1 Patient Screening

The investigator is responsible for keeping records of all screened patients, including patients entering the trial and patients who have been excluded. The above records should be included in the main test documents.

Patients must sign an informed consent form (ICF) before starting the screening process. After the patients have signed the ICF, each potential subject will be assessed for inclusion and exclusion criteria and recorded in the Case Report Form (CRF).

A complete medical history of each patient must be obtained, including all previous treatments, current medications, and all medications used in 28 days prior to enrollment.

Each patient will undergo a complete physical examination, which includes vital signs, weight and height. Patient demographic information will also be recorded. A standard 12-lead electrocardiogram (ECG) examination will be performed within 14 days prior to Day 1 to identify patients who is believed by the investigators that may has clinically significant abnormalities which have not been diagnosed. A tissue or blood sample for laboratory testing is required.

At the time of enrollment, each patient's ECOG status will be evaluated (see Annex 3: Eastern Oncology Cooperative Group (ECOG) - Physical Status Score Sheet).

All enrolled patients will be assessed periodically according to pre-arrangements

throughout the trial (see Annex 1: Study Flow Chart).

5.2.2 Patient enrollment

Once the patient is determined to be eligible to participate in the study, a validated standard information of inclusion and exclusion will be provided and sent to the primary investigator and clinical monitor for review by fax or email. When it is confirmed that the patient has met all the inclusion and exclusion criteria, the sponsor or his client will assign a unique patient identification number to the subject. If the patient discontinues the trial, the patient number will no longer be used, and the patient will not be allowed to re-enter the trial.

5.2.3 Patient dose distribution

The patient will be assigned to the dose level according to the disease type based on the time when the patient is enrolled in the trial. The patient will receive the prescribed dose level. If the DLT observation period is completed, the disease does not progress and the investigator judges that the subject may benefit, the subject will continue to be treated until the disease progresses or is intolerable.

5.2.4 Duration of the test

For each patient in the primary trial, the time to participate in the trial is expected to be around 12–24 weeks. This includes up to 28 days for screening, once every 2 weeks after treatment initiation, 2 consecutive doses, and continuous oral axitinib treatment for DLT observation, followed by administration once every 2 weeks, 4 times for one observation cycle. The recruitment time is estimated to be 6 months, and the total duration of the trial is about 12 months, so that there is enough time to collect patient data and solve relative problems. This does not include patients who are eligible to continue treatment during the extended period as described below.

Patients with no disease progression and no DLTs at the end of the first cycle of treatment, as the investigator judges may benefit, are allowed to continue receiving JS001 at the same dose level and with reference to the same schedule until disease progresses or DLT occurs. Treatment will continue until the withdraw criteria are reached.

6. Study population

This study will select patients with advanced renal and malignant melanoma who have not previously received sysmetic treatment, or no effective treatment, or who cannot tolerate existing treatment, or received limited systemic treatment. The specific inclusion criteria are as follows.

6.1 Inclusion Criteria:

Subjects may be entered in the study only if they meet all of the following

criteria：

- Male and Female aged between 18 and 75 years;
- Easter Cooperative Oncology Group (ECOG) performance status score of 0 or 1;
- Histologically confirmed advanced RCC or melanoma who have disease progression or intolerance after at least one dose of routine systemic treatment, or refused routine systemic treatment;
- Agree to provide fresh or archival tumor tissue specimens (fresh tumor tissue is preferred for detecting PD-L1 expression and infiltrating lymphocytes);
- At least one measurable lesion (Only one measurable lymph node lesion was excluded) (conventional CT scan ≥ 20 mm, spiral CT scan ≥ 10 mm, measurable lesions have not received radiotherapy) ;
- Life expectancy≥3 months；
- Patients may have a history of brain/meningeal metastases, but they must have local treatment (surgery/radiotherapy) prior to the start of the study and be clinically stable for at least 3 months (prior corticosteroids use is allowed, but patients with concurrent use of systemic corticosteroids should be excluded)；
- Sufficient Organ function (within 14 days before enrollment)：

  （1）Must meet the blood routine examination standards (no blood transfusion or G-CSF treatment within 14 days)：

  HB ≥90 g/L；

  ANC≥1.5×10^9 /L；

  PLT≥100×10^9 /L；

  （2）Non-functional organic diseases, meeting the following criteria：

  TBIL≤1.5×ULN（Upper Limit Of Normal）；

  ALT and AST≤2.5×ULN； With hepatic metastasis: ALT and AST≤5×ULN；

  Serum Cr≤1.25×ULN, and Ccr＞50ml/min（Cockcroft-Gault fomula）；

  International normalized ratio (INR), activated partial thromboplastin time (aPTT): ≤1.5×ULN (this standard only applies to patients who have not received anticoagulant therapy; patients who receive anticoagulant therapy should place anticoagulants in the range of treatment requirements);

  Urine protein ≤ 1 +; if the urine protein > 1 +, collect 24 hours urine protein determination, the total amount should ≤ 1 gram;
- No systemic corticosteroids is received within 4 weeks prior to treatment；
- Males with fertility or women who may become pregnant must use highly effective methods of contraception (such as oral contraceptives, intrauterine devices, libido or barrier contraceptives combined with spermicides) during the trial. Continue contraception for 12 months after the end of the therapy;
- Subjects must join the study voluntarily, must sign informed consent, and agree to

comply to follow-up.

6.2 Exclusion Criteria

Subjects fulfilling any of the following conditions cannot be enrolled in the study:

- Previous treatments with anti-PD-1 antibody, anti-PD-L1 antibody, anti-PD-L2 antibody or axitinib;
- Known to be allergic to recombinant humanized anti-PD-1 monoclonal antibody drugs and its components;
- Received other anti-tumor treatments (including corticosteroids, immunotherapy) or participating in other clinical studies within 4 weeks prior to the start of treatment, or haven't recovered from the last toxicity (except for grade 2 alopecia and grade 1 neurotoxicity);
- Pregnant or lactating;
- Positive for HIV;
- Active hepatitis B or hepatitis C patients:

  Positive test for HBsAg, with HBV DNA copies detected as positive (quantitative measurements ≥ 500 cps/mL); HBV DNA must be detected during screening of such patients;

  Positive test for chronic Hepatitis C in blood screening test (HCV antibody positive); patients with positive HCV antibody test results can only be included in the study when the PCR results of HCV RNA are negative.
- Diagnosed with active tuberculosis (TB)；
- With a large amount of pleural or ascites with clinical symptoms or need to be treated symptomatically;
- With autoimmune diseases, or history of autoimmune diseases requiring systemic use of steroids/immunosuppressive agents, such as pituitary inflammation, pneumonia, colitis, hepatitis, nephritis, hyperthyroidism, thyroid function decrease, etc.;
- Other serious, uncontrollable concomitant diseases that may affect protocol compliance or interfere with the results, including active opportunistic infections or advanced (severe) infections, uncontrolled diabetes, cardiovascular disease (grade III or IV heart failure as defined by the New York Heart Association classification, heart block above grade II, myocardial infarction in the past 6 months, unstable arrhythmia or unstable angina, cerebral infarction within 3 months, or lung disease (interstitial pneumonia, obstructive pulmonary disease, and history of symptomatic bronchospasm);
- Subjects with active central nervous system (CNS) metastases. If the subject's CNS metastasis is adequately treated to meet the requirements specified in the inclusion criteria, and the subject's neurological symptoms can be restored to less than or equal to CTCAE grade 1 before enrollment (except for residual signs or symptoms

associated with CNS treatment), after at least 2 weeks, the subject can participate in the study;

- Received hematopoietic stimulating factors such as colony stimulating factor and erythropoietin within 1 week before the start of the study;
- Received live vaccination within 4 weeks prior to the start of the study;
- Received allogeneic hematopoietic stem cell transplantation or solid organ transplantation;
- Received major surgery (excluding diagnostic surgery) within 4 weeks prior to the start of the study;
- Subjects with a history of substance abuse and are unable to quit or with a history of mental disorders;
- With a history of other malignant tumors in the past 5 years, except for cured skin basal cell carcinoma and cervical carcinoma in situ;
- Other severe, acute or chronic diseases, or psychiatric disorders or laboratory abnormalities that the investigator judges may increase the risk of participating in the study or may interfere with the interpretation of the findings.

6.3 Patient withdrawal criteria

In the following cases, the patient will withdraw from the trial:

1) When the patient asks to quit.

2) Failure to comply with the requirements of the protocol, the investigator or sponsor determines that the patient is unable to continue the therapy.

4) The patient is pregnant.

5) Disease progression according to irRC and RECIST criteria

6) Have suspended JS001 and axitinib for any reason for more than 2 weeks

7) When the investigator judges that continued treatment is no longer appropriate for the patient.

6.4 Withdraw from the trial

During the study, subjects with disease progression (PD) according to the Immune-Related Response Criteria (irRC) and Response Evaluation Criteria in Solid Tumors (RECIST 1.1) will discontinue from the study. In addition, subjects may withdraw from the study at any time if a DLT or disease progression occurs. Subjects may withdraw from the study at any time for any reason. If a concurrent illness, adverse event, or protocol violation occurs, the investigator can also discontinue the subject from the study.

If the study is terminated due to toxicity, the subject will be followed for at least 30 days until any drug-related toxicity returns to grade 1. In the event of an unplanned termination, the investigator should make every effort to contact the subject for a final

evaluation.

7. Investigational Products

7.1 Investigational Products

The recombinant humanized anti-PD-1 monoclonal antibody injection is provided by the sponsor, specification: 240mg/6ml/bottle. The drug was diluted with saline to complete the administration by intravenous drip for one hour.

Axitinib tablets are provided by the sponsor. Specifications: 5mg/28 tablets/box. According to the clinical research and the listed drug instructions of axitinib, the dosage of axitinib combination is determined 5 mg bid, administered orally.

7.2 Package and Tag

Once the patients are verified to be administered, JS001 will be transported to GCP drug storage by the sponsor. Drug must be transported protected from light under temperature of 2-8°C. The drug must be provided by sponsor according to GCP standards.

As a marketed pharmaceutical product in China, Axitinib is packaged and tagged in accordance with national relevant regulations.

The design of the package of JS001 for trial use is as follows:

（1）Tag on bottle:

Batch No：2015L05752

Recombinant humanized anti-PD-1 mAb injection

（Only for clinical trials）

Strength: 240 mg/6 mL/vial

Drug Number：XXXX

Suzhou Zhonghe Biosciences Co., Ltd.

（2）Tag on box:

Batch No：2015L05752

Recombinant humanized anti-PD-1 mAb injection

（Only for clinical trials）

Strength: 240 mg/6 mL/vial

Suzhou Zhonghe Biosciences Co., Ltd.

【Usage and Dosage】A solution with a concentration of 1 to 10 mg/ml is prepared using 0.9% physiological saline than intravenously instilled. The dosage is determined according to the test protocol. When injecting this drug, an in-line filter (0.2 or 0.22 μm) should be used for intravenous injection within 60 minutes. In the case of an infusion reaction, the infusion rate can be slowed down, or the instillation can be interrupted, and the necessary treatment given

until the patient's symptoms improve, and the infusion continues at half the rate.

【Storage】Preseved from light, under cold storage of 2-8℃

【Period of validity】24 months

Batch No：201601001

Expiration Date：20180122

7.3 Management of Investigational Products

7.3.1 Reception and Storage of JS001

Investigators or authorized personnel are responsible for comparison of the drug labels with drug transportation list. Investigators or authorized personnel will check the accuracy of information on the table, sign it with signature and date, then return it back to the sponsor. The file would be rechecked during the trial process and retrieved after follow-up ends by monitors. Copies should be preserved in investigators record, in the sponsor' and in drug distribution office of the centers.

The study drug can only be used in this study and managed by the personnel authorized by the investigator. To completely control the dispensing and use of the study drug, inventory recording should be performed at each drug administration visit of the subjects.

7.3.2 Disposal of JS001

All unused JS001 will be stored in the storage location assigned by the clinical pharmacology institution of the study site according to specified storage conditions (protected from light and cold storage at 2-8℃). After finishing the trial, investigators should return the rest JS001s to sponsor or authorized individuals or dispose of them in the center according to relevant rules by demands of sponsor. If any drug is lost or damaged, the detailed condition should be recorded. All the partially delivered JS001s must be disposed of based on the demands set in protocol.

7.4 Administration

7.4.1 Dose determination basis

The dose levels planned for this dose escalation phase trial are: 1 mg/kg and 3 mg/kg Q2W.

According to the clinical PK parameter analysis in the phase I clinical trials of JS001 by Peking University Cancer Hospital and Sun Yat-sen University Cancer Hospital,

the drug basically reached the peak concentration level after the end of administration, and the half-life was about 5-12 days. At a dosing frequency of once every 2 weeks, JS001 trough blood concentration reached a steady state after 3 consecutive administration. The steady-state trough concentrations were about 1.5 μg/ml, 8 μg/ml, 25 μg/ml and 85 μg/ml at the dose of 0.3 mg/kg, 1 mg/kg, 3 mg/kg and 10 mg/kg respectively. A dose of 3 mg/kg Q2W achieved a desired peripheral blood concentration.
At the same time, according to the PD-1 receptor occupancy (RO) analysis of the peripheral blood T lymphocytes in the phase I clinical trial of JS001 by Peking University Cancer Hospital and Sun Yat-sen University Cancer Hospital, all test dose groups, including 0.3, 1.0, 3.0, 10.0 mg/kg, and 240 mg fixed-dose every 2 weeks, maintained an average receptor occupancy rate of more than 80%.

Based on the PK/PD results of the JS001 Phase I clinical studies, as well as observed clinical efficacy in the 1 mg/kg and 3 mg/kg dose groups, the recommended initial dose of JS001 combined with axitinib is 1 mg/kg, with dose escalation to 3mg/kg.

The dose of axitinib remained unchanged. Therefore, there are totally four groups of advanced RCC and melanoma in two dose levels, 3-6 cases in each group:
Advanced RCC: 1.JS001 1mg/kg Q2W + axitinib 5mg Bid
2.JS001 3mg/kg Q2W + axitinib 5mg Bid
Advanced melanoma: 1.JS001 1mg/kg Q2W + axitinib 5mg Bid
2.JS001 3mg/kg Q2W + axitinib 5mg Bid

For each dose group, JS001 was administered on the first day, and axitinib was administered orally on the second day.

7.5 Drug preparation and record of JS001

JS001 is sterile, colorless, liquid with/without opalescence. It should be checked closely before usage to confirm that no damage on the bottles and no aggregation, turbidity or deposition within. After the check, use 0.9% saline to dilute the liquid to the final concentration of 1.0-10 mg/ml under sterile condition.

The bottles should be preserved in a dark place at 2-8°C. The prepared infusion solution can be maintained at room temperature for up to 4 hours. If the prepared solution could not be immediately used, it should be preserved at a fridge (2-8°C) for up to 16 hours. Because JS001 does not contain any antibiotics, the preparation must be conducted under sterile situation.

The volume of the study drug infused into subjects would be accurately recorded during the trial.

7.6 Precautions when adminstrating JS001

1) prophylactic treatment

Any infusion reaction must be treated in time. When infusion reaction occurs, every administration afterwards should be pretreated with antipyretic analgesics (e.g. paracetamol), antihistamines (e.g. diphenhydramine) and glucocorticoids (e.g. dexamethasone) within 30-60 minutes before infusion starts for prophylactic use, in order to lower the risk of infusion reaction to happen again.

The recombinant humanized anti-PD-1 mAb should never be injected intravenously or quickly. The peripheral or central IV channel is recommended. Before infusion, sufficient epinephrine, diphenhydramine and recovery devices must be prepared for potential serious allergy reactions. After infusion, the IV channel should remain open for potentially needed drug delivery. Given no complications, the IV channel could be withdrawn following 1 hour of post-infusion observation.

2) IV infusion speed

To infuse JS001, an inline filter (0.2 or 0.22μm) should be used for IV infusion. If infusion reaction occurs, the dripping speed should be decreased or stopped for necessary measures. The dripping speed could be recovered to half of the original speed when the infusion reaction is improved.

3) monitor during IV infusion

The first use of JS001 should be under guidance of experienced doctors. The vital signs (temperature, breath, BP, HR), color of face, sweat or headaches must be monitored before, within and 1 hour at minimum after infusion to discover any signs of infusion reactions on early stage.

Some of the infusion reactions may happen in later administrations, which should be monitored by doctors even if no infusion reactions presented at the first infusion of JS001. As for the patients with serious infusion reactions like severe dyspnea, bronchospasm and hypoxia, the dripping must be stopped immediately. When all the symptoms vanish and lab test results recover, investigator could decide if the infusion should be continued; if it is continued, the infusion speed should not be more than half of the original speed. If the same infusion reaction occurs again, permanent discontinuation should be considered.

4) measures for infusion reactions

In spite of the preventative use of antihistamines and glucocorticoids for allergic reactions before administration, the infusion of an antibody could still cause infusion reactions. Investigators may use different measures to treat infusion reactions per NCI CTCAE v4.03: generally grade I/II reactions do not need treatment other than lowering infusion speed and monitoring of the infusion process; for those who have grade III/IV reactions (like bronchospasm, body temperature>40.0℃, etc.), the infusion must stop immediately, and corresponding treatments must be delivered ASAP, such as bronchial antispasmodic, epinephrine, cortical steroids, antihistamines,

fluid infusion, oxygen supplement therapy and close monitoring. Investigators should decide if the infusion continues after the symptoms are totally relieved.

When serious reactions happen, including serious dyspnea, hypotension and cardiac problems which could not been relieved by discontinuation of infusion, glucocorticoids can be used. Once allergic shock symptoms are found, the anti PD-1 mAb injection should be discontinued immediately, and the patients should be lied flat and given oxygen therapy.

7.7 Concomitant drug/treatment

During the study, investigators are permitted to deliver supportive treatments to subjects according to clinical needs, as following description. The off-protocol anti-tumor treatments, like surgery, radiotherapy, etc., are in principle prohibited, but the treatments toward tumor emergency which might cost patients' lives are excluded.

Permitted concomitant drugs

All concomitant medications will be recorded on the Case Report Form (CRF) during the study period, including all prescription, over-the-counter (OTC), and intravenous medications. If a change has occurred during the test, the dose, frequency, route of administration and date of the drug are recorded in the CRF.

Prohibited concomitant drugs

During the screening and treating periods, subjects are forbidden to receive:

- Systemic anti-tumor chemotherapy or biological therapy;
- Immunotherapy other than JS001;
- Investigational drug other than Axitinib;
- Radiotherapy (excluding local radiation therapy treating bone metastatic lesions);
- Vaccination within 4 weeks before/during administration, including but not limited to the vaccines for measles, epidemic parotitis, rubella, varicella, yellow fever, rabies, BCG and typhoid(oral);
- Any glucocorticoid other than treatment for adverse events due to immunotherapy (remarks: physiological doses of steroids may be allowed after communication with the sponsor);
- Inhibitors or inducers of CYP3A4/5;
- In general, traditional Chinese medicine (TCM) is not recommended during the study, and any use of TCM requires consultation with the sponsor.

7.8 Drug compliance

The subject's administration will be accurately recorded during the trial.
The dose and time of administration for each subject should be recorded in the medical record and CRF. The reasons for delayed dosing, drug reduction, or missed

delivery should also be recorded in the medical record and CRF, and the subjects should use the drug as required by the protocol.

7.9 Standard for dose adjustment of the combination administration

In this study, no dose adjustment of JS001 (a recombinant humanized anti PD-1 mAb) injection would be allowed, while JS001 administration should be discontinued due to any of following situation:

- Any toxicity defined as JS001 related DLT. See 5.1.4: Definition of dose-limiting toxicity (DLT);
- Any Grade 3 and above non-DLT toxic reaction (hypertension or hand foot skin reaction).
- Hematological, blood biochemical and organ functional lab tests

—serum AST：>5.0 × ULN；

—serum ALT：>5.0 × ULN；

—total bilirubin：> 3.0 × ULN；

—platelets：<50,000/μL；

—ANC：<1,500/μL；

—serum creatinine >2.0 × ULN。

If patients have been recovered from AEs within 2 weeks, they could be re-administered. No more administration of JS001 for the patients who have had any JS001-related DLT.

During the study and treatment, if ≥grade 3 hypertension or HFS occurs, axitinib treatment will be suspended until the symptoms improved to ≤ grade 1, and then give axitinib again, but the dose will be adjusted to 5 mg QD. If there is still no improvement in 4 weeks, stop treatment.

If it is not possible to determine which drug the adverse reaction is due to, one should first consider suspending JS001. If the related adverse events cannot improve, consider suspending axitinib.

8 Research visit and evaluation

Patients enrolled in the study will be regularly visited and assessed during the trial period in accordance with the prescribed visit assessment process. (See Annex 1: Research Flow Chart).

During clinical trials, if there are clinical indications, an unscheduled assessment will be conducted. Data for unplanned evaluations should be recorded on the CRF.

8.1 Informed consent

The patient (or authorized representative) is required to sign the IRB-approved Informed Consent (ICF) by the researcher at the research site or an authorized person. An original copy will be given to the patient and another original copy will be kept at

the base. There must be a trial title, version number, and date on each page of the ICF. Informed consent must be obtained before the patient performs any procedure specific to the trial.

8.2 Medical history

The medical history includes the state of various concurrent diseases. Researchers need to have an overall medical history of the previous and current disease and information about the drug currently in use. Special attention points on the specific tumor type should also be provided.

8.3 Demographics

Demographic data includes race, age, and gender. After the patient has signed the ICF, demographic data will be recorded at screening.

8.4 Medical examination

Comprehensive routine examination of the main body systems (eyes, ENT, cardiovascular, respiratory, gastrointestinal, musculoskeletal/connective, neurological, endocrine/metabolism, hematopoietic/lymphatic, dermatological, mental, genitourinary) are carried out. It's recorded whether the patient has a known drug reaction or other allergies. At subsequent follow-up, repeated physical examinations of the same body system will be performed at screening time (1–14 days prior to Day 1), before each JS001 treatment is initiated, and at the end of follow-up to see if a significant change from baseline is present. Height will only be measured at screening.

8.5 ECOG score

The ECOG PS score should be taken at screening (1–14 days prior to Day 1), before each cycle of treatment, and at the end of follow-up.

8.6 ECG

A standard 12-lead electrocardiogram (ECG) examination should be performed within 28 days prior to day 1 to identify patients who have not yet been diagnosed with clinically significant abnormalities. ECG examination was performed before the start of JS001 treatment in each cycle and at the end of follow-up.

8.7 Vital signs

Vital signs: including body temperature, heart rate, respiration, blood pressure, multiple dose test: before the first dose, every 15 minutes after the start of administration, 30 minutes after the end of the dose, 1 time each test; follow-up Dosing every 30 minutes before administration, during administration, and within 1 hour after the end of administration: blood pressure, respiration, pulse, and body temperature are

measured once. If abnormal, increase the number of measurements and extend the measurement time to normal. After 30 minutes;

8.8 Laboratory inspection

The following laboratory assessments must be performed throughout the trial. The assessment will be conducted in the laboratory where the research center is located.

1) Blood routine examinations, including complete blood count (CBC) and white blood cell (WBC) differential counts, red blood cell (RBC) counts, and platelet counts

2) Women of childbearing age are required to undergo serum or urine pregnancy tests

3) Blood chemistry: total protein, albumin, total bilirubin, aspartate aminotransferase (AST), alanine aminotransferase (ALT), alkaline phosphatase (ALP), lactate dehydrogenase (LDH), total cholesterol, HDL, LDL, triglycerides, uric acid, blood urea nitrogen (BUN), creatinine, creatinine clearance, electrolytes [sodium (Na+), potassium (K+), chlorine (CI-)], calcium , phosphorus, amylase, blood sugar and C-reactive protein (CRP).

4) Coagulation: prothrombin time (PT), activated part thromboplastin time (aPTT), fibrinogen (FIB) and international normalized ratio (INR).

5) Thyroid function test: thyroid stimulating hormone (TSH), serum free triiodothyronine (FT3), serum free thyroxine (FT4).

6) Viral testing: full hepatitis B, hepatitis C, HIV (HIV), syphilis test (TPPA), HBV-DNA copy number (during 4 weeks before treatment);

7) Lymphocyte subsets: T cell population (CD3+, CD4+, CD8+), natural killer cells (CD16/56+), immunoglobulin (IgG, IgA, IgM); complement (C3) (taken within 7 days prior to treatment));

8) Urine analysis: urine sugar, urine protein, urobilinogen and other measurements.

As for blood draw and treatment methods, laboratory procedures of research institutions should be followed. If the patient is female, in order to be enrolled in the trial, the patient must receive birth control surgery or at least 1 year after menopause, or effective contraception should be taken during the trial and for 12 consecutive months after the last dose. If the patient is male, the patient must agree to take effective contraception during the trial and for the 12th consecutive month after the last dose.

8.9 Tumor evaluation

Imaging evaluation of the tumor at baseline (1–28 days before day 1), including: head, chest, abdomen, and basin (if there are other sites, the corresponding site needs to be examined; preferred enhanced CT, if the subject for allergic reactions to contrast agents, MRI or CT scans can be used), bone scans. During treatment follow-up, refer

to the tumor immunotherapy efficacy evaluation (irRECIST) and RECIST1.1 criteria, in multiple doses (administered once every 2 weeks, 4 times per cycle) every 2 cycles (± 2 weeks) and withdrawal follow-up (Only if the tumor progression has not been determined), the patient underwent a chest, abdomen, and pelvic examination for tumor evaluation. If clinical indications were included, a CT/MRI scan and a bone scan should be included. Osteolytic lesions or osteolytic-osteogenic lesions with defined soft tissue components as assessed by CT or MRI transverse slice imaging techniques can be considered as measurable lesions of soft tissue components.

The same imaging method as the disease at baseline should be used throughout the trial. In addition, tumor size and necrosis were calculated by an independent radiologist. Details on the collection procedures for imaging data will be further described in the Image Guidelines.

8.10 Immunogenicity test

ADA-generated conditions were examined to assess the effect of antibody production on the efficacy and safety of the study drug, as well as the pharmacokinetics.

Specimen collection time: before the first injection, before the third injection, before the fifth injection, and before each two injections until the end of the treatment, blood collection 3 ml / time.

8.11 Pharmacodynamic test

In the course of this study, the PD-1 receptor occupancy in the blood will be tested to assess the inhibition of the target drug by the study drug, and the PK/PD exploration analysis is planned.

Receptor occupancy sampling time: before the first injection, 24 hours after the end of the first injection, before the second injection, before the third injection, blood collection: 12ml (3ml / time, a total of 4 times).

8.12 Tumor sample collection

Pathological tissue specimens should be taken before the patient is enrolled in the study; if possible, biopsy fresh specimens should be taken as much as possible. At the same time, the biopsy tissue specimens for evaluating the progress of the disease or achieving effective remission were collected for the detection of PD-L1 expression. All specimens that have been collected for biomarker evaluation are processed by the research center laboratory. The Research Center Laboratory will provide a full range of equipment to collect and transport these specimens to designated laboratories for analysis.

The newly acquired primary tumor tissue (or site of metastasis if the primary tumor is not obtained) should be obtained no later than 2 weeks before the first dose.

The obtained specimens will be fixed in 10% formalin, embedded in paraffin, serially sectioned, and stained with H&E for tumor diagnosis; immunohistochemical staining will be performed at the central laboratory.

Detailed information on handling procedures, sample storage and transportation will be described in a separate laboratory manual. Tumor analysis will be conducted under the CFDA Guidelines for Non-Clinical Testing Quality Management (GLP) guidelines. Specimen collection information will be recorded in the specimen request form and CRF.

8.13 Combination therapy

All prescription and over-the-counter medications, including supportive care, Chinese herbal preparations, etc., must be recorded on the CRF from 28 days prior to the first study administration to the end of the trial.

8.14 Evaluation of adverse events

All adverse events from the time of the first dose of recombinant humanized anti-PD-1 monoclonal antibody injection to the end of the trial should be recorded. The patient will be informed to report all adverse events and to ask the patient's health status questions at each time of vital sign measurement. All adverse events, whether reported spontaneously or inquiries, should be recorded on the CRF.

According to the results of preclinical toxicity test of recombinant humanized anti-PD-1 monoclonal antibody injection and the experience of the same target drug, the inflammatory reaction of the gastrointestinal tract, kidney, liver, thyroid, lung, etc. caused by immunogenicity, and allergy Weakness, rash, loss of appetite, and diarrhea need to be focused on research.

When a patient temporarily discontinues or permanently discontinues a study drug due to abnormal AE or laboratory findings, it must be followed at least once a week for 4 weeks, followed by every 4 weeks until the event is alleviated or achieved. Steady state (whichever occurs first). If the patient needs to delay the administration time by more than 14 from the date of the scheduled administration, the patient must withdraw from the study. However, the patient's toxicity should be followed up as described above. After the last study drug infusion, all patients were followed up on the 30th day after the last dose, and follow-up to 90 days to investigate the occurrence of serious drug-related adverse events.

9. Safety assessment

9.1 Parameters of Safety Assessment

The main focus of this trial is patient safety at all time points. Adverse events will be monitored during treatment and 30 days after the last administration or until the event is remitted or stabilized.

The following parameters will be included:

1. Incidence and severity of adverse events;
2. Incidence and severity of clinical laboratory outcomes beyond the prescribed limits, including hematology, blood biochemistry, coagulation, etc.
3. Incidence and severity of dose-limiting toxicity;
4. Assessment of major medical changes, including physical findings, vital signs and ECGs.

9.2 Adverse Events (AE)

9.2.1 Definition of Adverse Events (AE)

Adverse events refer to all kinds of adverse medical events that may or may not have a causal relationship with treatment during treatment or during administration.

The name of adverse event should be recorded on CRF with the name of diagnosis or disease. If the name of adverse event cannot be limited or the name of diagnosis or disease cannot be used as the name of adverse event, clinical symptoms or symptoms will be recorded on CRF as the name of adverse event.

9.2.2 Laboratory abnormal results

Researchers should consider the following guidelines when deciding whether laboratory numerical changes are adverse events:

1. Abnormal laboratory results lead to changes in the use of experimental drugs (e.g. suspension of administration or permanent diagnosis).
2. In order to alleviate abnormal laboratory outcomes, combined and/or surgical interventions are needed.
3. Abnormal laboratory results are associated with clinical symptoms.
4. Abnormal laboratory results are associated with severe adverse events.
5. When researchers decide that abnormal laboratory results should be adverse events.

9.2.3 Serious adverse events (SAE)

A serious adverse event refers to any adverse medical event which meets one or more of the following criteria:

1. Results in death;
2. Is life-threatening;
3. Requires hospitalization or prolongation of existing hospitalization; Note: In this study, the following hospitalizations do not belong to SAE:

Patients were not hospitalized (except for important medical or life-threatening events) when they visited the emergency room or other departments of the hospital for less than 24 hours.

Selective surgery scheduled before signing the informed consent.

According to the research plan, the patients were hospitalized for the original medical operation/surgical operation.

Routine medical examinations (e.g. routine colonoscopy) that require hospitalization to assess the baseline level/trend of health status.

It is not to save the treatment of disease, but to be hospitalized in the Department of internal medicine/surgery before entering the study; but these situations need to be documented.

Hospitalization due to other living conditions has nothing to do with health status and does not require medical/surgical intervention (such as lack of housing, financial constraints, temporary absence of caregivers, family environment, management reasons).

In the absence of any other SAE, the patient was admitted to hospital for anticancer treatment.

4. Results in disability/incapacity; leading to permanent or significant disability/disability;
5. Is a congenital abnormality/birth defect of newborns/infants;
6. Other major medical diseases.

In other cases, if a major medical event may not immediately endanger life or cause death or hospitalization but may endanger the patient or may require intervention to prevent one of the other consequences listed in the above definition from occurring, whether the report should be expedited should be decided by medical and scientific judgement.

9.2.4 Causality Assessment

The relationship between adverse events and experimental drugs will be determined by researchers based on their clinical judgments and the following definitions:

Certainly unrelated

This category applies to a class of adverse events that are clearly and indisputably only external causes (disease, environment, toxic factors, etc.) and do not meet the low probability, high probability or clear drug-related criteria listed below.

Possibly unrelated

If there are the following circumstances (the first two must be required), adverse events are considered as potentially unrelated adverse events:

There is a time-sequence relationship between events and the administration of research drugs, but it does not occur at the same time.

There is reason to believe that events are caused by clinical conditions, such as environmental or toxic factors or other treatments given to patients.

Events are not consistent with known characteristics of reactions to research drugs.

Whether or not the suspension or reduction of doses of research drugs has alleviated or improved remains unclear or lacks information.

Possibly related

This category applies to such adverse events that cannot be explicitly excluded

from their relationship with research drugs. Adverse events can be regarded as possible related adverse events if the following conditions are met (the first two items must be required):

There is a reasonable chronological relationship between events and drug administration.

Events are caused by clinical conditions, such as environmental or toxic factors, or other treatments given to patients.

Events are consistent with known adverse drug reaction characteristics of research drugs.

- Suspension of dosage or reduction of dosage of research drugs to alleviate or improve events.

Related

This category is applicable to such adverse events that are highly clearly considered to be related to the study of drugs. Adverse events can be regarded as affirmative related adverse events, if the following conditions are met (the first two items must be required):

There is a reasonable chronological relationship between events and drug administration.

There is no reason to believe that events are caused by clinical conditions, such as environmental or toxic factors or other treatments given to patients.

Events are consistent with known adverse drug reaction characteristics of research drugs.

- Suspension of dosage or reduction of dosage of research drugs to alleviate or improve events.

Events reappear after re-administration.

9.2.5 Adverse Drug Reactions (ADR)

All harmful and accidental reactions that are dose-related and cannot be ruled out as drug-related should be regarded as adverse drug reactions. If there are such causal relationships as "possibly unrelated", "possibly relevant" or "affirmative correlation", AE will be treated as ADR.

9.2.6 Assessment of severity

The severity of AE will be evaluated with reference to CTCAE version 4.0. If adverse events do not fall within the scope of this criterion, researchers must categorize the severity of each adverse event according to clinical judgment. The severity of CTCAE version 4.0 is defined as follows:

Grade 0 No adverse events occurred or within normal limits.

Grade 1 Mild adverse events

Grade 2 Medium-to-moderate adverse events

Grade 3 Serious and unpleasant adverse events

Grade 4 A life-threatening or disability-causing adverse event.

Grade 5 Deaths associated with adverse events

9.2.7 Assessment of severity

Classification of severity is determined by referring to the following criteria:

1. Yes.
2. No.

9.2.8 Measures taken for the treatment of patients

Measures taken to treat patients are defined as follows:

1. Yes (to be explained);
2. No.

9.2.9 Measures taken to test drugs

The measures adopted for testing drugs are defined as follows:

1. Interruption of test drugs;
2. Suspension of administration;
3. No.

9.2.10 Final result

The final results of AE are defined as follows:

1. Resolved without sequelae

Patients have recovered from adverse events without any residual reactions.

2. Improved without sequelae

The patient has almost recovered from adverse events without any residual response.

3. Resolved with sequelae

Patients have recovered from adverse events with residual reactions.

4. Not resolved

The patient did not recover from adverse events.

5. Lethal

The patient died of adverse events.

9.3 Recording and reporting of adverse events

During each required follow-up during the trial, all adverse events that have occurred since the previous follow-up must be recorded. Researchers must determine the severity of each adverse event and the relationship between each adverse event and the drug tested (see Section 10.2.4: Causality Assessment and Section 10.2.6: Severity Assessment).

9.3.1 Recording of adverse events

All AEs/SAEs appearing during clinical trials should be recorded on CRF as soon as possible after receiving patient reports. The collection of AEs/SAEs began on the first day.

The following information for each adverse event should be collected:

1. Duration (occurrence date, mitigation date);
2. Causality;
3. Severity;
4. Seriousness;
5. Measures taken for the treatment of patients;
6. Measures taken to test drugs;
7. The final result.

9.3.2 Report of serious adverse events

Whether or not it is related to treatment, during treatment or within 90 days of the last treatment, the guardian shall immediately take appropriate protective measures against all serious adverse events of the subjects and report to the supervisor by telephone within 24 hours. The guardian shall also report in writing to the State Food and Drug Administration, the Ethics Committee and the sponsor, and sign and date the report.。These adverse events will be followed up until they are remitted or stabilized at an acceptable level for researchers.

At the end of the follow-up period, the researchers were not responsible for initiatively collecting new adverse events. However, after the trial phase, including the end of the follow-up period, researchers must report SAEs in the same way as above if they have reason to believe that they are related to the drug in question.

9.4 Pregnancy report and follow-up

When researchers learn that a female patient is pregnant, they must ask the patient to withdraw from the trial and follow up until the termination of pregnancy or delivery. When a researcher learns that a male patient's spouse is pregnant, the researcher must obtain his or her spouse's written informed consent and follow up the pregnancy as much as possible. Researchers should report all pregnancies to the sponsor immediately.

9.5 Data and Safety Monitoring Board (DSMB)

The trial will be supervised by an independent data and safety monitoring committee composed of three clinicians. The committee will meet regularly to discuss the development of the trials, with particular attention to the occurrence of various dose-limiting toxicities, increasing doses and whether the maximum tolerable dose has been achieved (focusing on the cumulative toxicity of various levels 4 in long-term use).DSMB will specially assess adverse event data, as well as summary reports of all serious adverse events and laboratory data obtained. DSMB can also assess individual cases if necessary or when measures must be taken to determine whether safety problems occur. After evaluation, DSMB may recommend new measures for specific treatment groups, or other appropriate measures to illustrate possible safety problems in routine trials. DSMB may, if necessary, recommend that the entire test program be

suspended at any point in time or that the entire test be permanently interrupted.

Regular DSMB meetings will be held at the following event points:

All three patients in a dose group have completed the observation period in turn and can start recruiting the next dose group.

When MTD is expected to be found;

- If level 4 toxicity occurs during the extension period, further meetings will be held at the request of DSMB or bidders. The safety data assessment will be recorded in a separate document (see Annex 8: Statute of the Data and Safety Monitoring Committee).

10. Pharmacokinetic evaluation

10.1 Evaluation parameters

In this experiment, the pharmacokinetic parameters of recombinant humanized anti-PD-1 monoclonal antibody will be evaluated by non-atrioventricular model method as follows:

1. Serum drug concentration at each sampling time point;
2. Peak time (T_{max})
3. Serum peak concentration (C_{max}) and dose normalized C_{max} (C_{max}/D);
4. Area under serum concentration-time curve (AUC_{0-t});
5. The final elimination rate constant (λ_z) and the end half-life ($t_{1/2}$);
6. To infinite time AUC ($AUC_{0-\infty}$);
7. Apparent Distribution Volume (V_d);
8. Distributed volume (V_{dss}) at steady state;
9. Total clearance rate (CL);
10. Average residence time (MRT).

10.2 Assessment time point

Blood samples were collected for PK evaluation during dose escaltion and expansion.

Dose escalation phase: Continuous plasma samples were collected before administration of each recombinant humanized anti-PD-1 monoclonal antibody injection for determination of plasma concentration-time curve. Samples of trough concentration before administration of each recombinant humanized anti-PD-1 monoclonal antibody injection were collected from all patients in subsequent cycles.

Expansion phase: Peripheral blood samples (about 8ml) will be collected before and after the treatment of recombinant humanized anti-PD-1 monoclonal antibody injection, and at the end of each therapeutic evaluation stage, peripheral blood samples (about 8ml) will be collected.

10.2.1Blood collection of recombinant humanized anti-PD-1 monoclonal antibody

The time of blood collection was shown in the table below. Blood samples were collected from elbow vein at 5.0 ml (the time point of blood collection was shown in Table 8). Serum was separated and frozen at - 80℃. The frozen samples are transported from the testing unit to the PK testing unit through a professional cold chain.

Within 30 minutes (0 hours) before first administration, immediately after administration (within 5 minutes after administration), 0.5 hours, 2 hours, 6 hours, 24 hours, 48 hours, 96 hours and 168 hours after administration, within 30 minutes (0 hours) before the beginning of the second and third administration and 0.5 hours after administration, within 30 minutes (0 hours), 0.5 hours, 2 hours, 6 hours, 24 hours, 48 hours, 96 hours, 168 hours and 336 hours after the fourth administration, JS00Serum samples were collected within 30 minutes (0 hours) before and 0.5 hours after the first four times of single administration.

Table 8. Time Points of Blood Collection in PK Study

Follow up time	Blood collection time point	Testing items
1. First administration (day 1)	Before medication	JS001 PK, ADA, RO
	Immediately after infusion	JS001PK
	0.5Hours (+5 minutes)	JS001PK
	2Hours (+5 minutes)	JS001PK
	6Hours (+15 minutes)	JS001PK
2. First administration (day 2)	24Hours (+1 hours)	JS001PK, RO
3. First administration (day 3)	48Hours (+1 hours)	JS001PK
4. First administration (day 5)	96Hours (+1 hours)	JS001PK
5. First administration (day 8)	168Hours (+1 hours)	JS001PK
6. Second administration (day 1)	Before medication	JS001PK, RO
	0.5Hours (+5 minutes)	JS001PK
7. Third administration (day 1)	Before medication	JS001 PK, ADA, RO
	0.5Hours (+5 minutes)	JS001PK
8. Fourth administration (day 1)	Before medication	JS001PK
	0.5Hours (+5 minutes)	JS001PK
	2Hours (+5 minutes)	JS001PK
	6Hours (+15 minutes)	JS001PK

9. Fourth administration (day 2)	24Hours (+1 hours)	JS001PK
10. Fourth administration (day 3)	48Hours (+1 hours)	JS001PK
11. Fourth administration (day 5)	96Hours (+1 hours)	JS001PK
12. Fourth administration (day 8)	168Hours (+1 hours)	JS001PK
13. Fourth administration (day 15)	336Hours (+1 hours)	JS001PK
14. The fifth dose (day 1) and every two subsequent doses	Before medication	JS001ADA

In order to reduce the pain or infection caused by repeated venipuncture, venous blood was collected regularly by left elbow venipuncture with disposable venous indwelling needle.

Details of PK sample analysis method, sample processing flow, sample storage and transportation convenience will be described in a separate human pharmacokinetic scheme.

* If no samples or analysis data are obtained at the time of withdrawal from follow-up, the remaining blood samples collected for PK samples can also be used for the determination of tumor markers.

11. Efficacy evaluation

11.1 Tumor evaluation

The objective response, the time of disease progression and the duration of response will be evaluated by irRC and RECIST. At baseline, the researchers assessed the tumor burden through physical examination and imaging. Tumor markers are also used for evaluation, but they cannot be used alone for response evaluation. At the end of each two cycles of treatment after multiple consecutive doses, the researchers used the same detection techniques to confirm the response of tumors.

11.2 ECOG PS

ECOG PS assessments were performed at screening and subsequent weekly treatments to assess how the disease affects the patient’s ability to live on a daily basis (See Annex 3: ECOG-Physical State Score Table).

12. Statistical analysis

12.1 Data sets

12.1.1 Safety

All patients who have been selected for the trial and have received at least one recombinant humanized anti-PD-1 monoclonal antibody injection will be included in

the safety analysis. A complete trial treatment cycle has been completed, including the population assessed for DLT assessment.

12.1.2 Pharmacokinetics

All patients who have received the test drug and have at least one PK data result, including in the PK analysis set.

12.1.3 Full Analysis Set (FAS)

All patients who received at least one recombinant humanized anti-PD-1 monoclonal antibody was included in FAS analysis.

12.2 Processing of missing data

For missing data, no numerical allocation will be made. Standard clinical monitoring and data management specifications will be used to ensure data integrity.

12.3 Analysis method

Statistical analysis is mainly descriptive, because the purpose of this experiment is to determine the MDD of recombinant humanized anti-PD-1 monoclonal antibody. Therefore, no statistical hypothesis test has been arranged.

For continuous variables, standard descriptive statistics include mean, standard deviation, minimum and maximum, while for categorized variables, they include quantity and percentage.

Descriptive statistical indicators will be classified according to the dose level (initial dose level/patient) and the combined descriptive statistical indicators will be given for all patients.

12.3.1 Demographic and baseline disease characteristics

Continuous variables will use descriptive statistics, aggregate demographic and baseline disease characteristics data, and list the categorized variables. Safety analysis will be categorized by dose group and descriptive results for the general population will be given. The data presented in the list will include at least one demographic feature, such as gender, age and race, as well as disease-specific status (e.g. ECOG physical status, past treatment, etc.) and medical history.

12.3.2 Safety

All safety data will be summarized according to dose groups of recombinant humanized anti-PD-1 monoclonal antibody. The incidence of DLT in single/multiple doses and in the whole trial will be summarized. In addition, the causes of DLT will be summarized according to the patient classification list.

Adverse events will be expressed in terms of MedDRA-preferred terminology and body system, and related adverse events (judging possible unrelated, possible related, affirmative related adverse events), serious adverse events, interruption tests caused by adverse events, and adverse events at Level 3 and above will be listed separately.

The vital signs data, ECG data, laboratory data and findings from physical examinations will be presented in a time table.

Laboratory events will be classified according to dose level and summarized in a

list (at least one level of laboratory test results will be added to the classification). Laboratory values at Level 3 and above will be classified according to dose level.

12.3.3 Pharmacokinetics

Non-atrioventricular model was used to analyze serum drug concentration and PK parameters (T_{max}, C_{max}, C_{max}/D, AUC_{0-t}, AUC_{0-t}/D, $AUC_{0-\infty}$, λ_z, $t_{1/2}$, V_d, V_{dss}, CL, MRT).

12.3.4 Effectiveness

All validity data will be analyzed based on FAS population.

12.3.4.1 Antineoplastic activity

Medical examination and imaging (CT or MRI) will be used to evaluate the efficacy of tumors in patients with measurable lesions at screening, after 2 cycles of treatment and after withdrawal from follow-up, according to the criteria of IRRC and RECIST version 1.1. The same measurement method as the baseline was used throughout the experiment. The main efficacy indicators are defined as follows:

- ORR is defined as the proportion of patients who achieves complete response (CR) or partial response (PR); response was determined by researchers using RECIST v1.1. Patients who did not undergo any assessment were considered to have no response.
- PFS is defined as the time from randomization to first recording of disease progression, or death from any cause, whichever occurs first. Patients who did not have any incidents during follow-up or treatment will be deleted from the final cancer assessment. Patients who did not undergo post-baseline assessment were deleted at random.
- DOR is defined as the time from the first recorded response (CR or PR) to the first recorded disease progression or death (whichever occurs first). These variables were calculated only for patients who achieved optimal overall response of CR or PR. Patients who have not progressed or died after response will be deleted on the date of the last tumor test.
- OS is defined as the time from randomization to death for any cause. Patients with no incident will be deleted from the known final survival date. Patients who do not provide any follow-up information will be deleted at random.

12.3.4.2 ECOG PS

The descriptive statistical results of ECOG PS are given according to the classification of visiting time points.

12.4 Estimated number of patients

There is no formal sample size calculation in this experiment. The expected number of patients with incremental doses ranges from 18 to 24.The actual sample size will be determined according to the actual dose-limiting toxicity induced during the dose process. The extended research phase plan will be determined based on the final

determination of the extended dose and effectiveness observation.

12.5 Mid-term analysis

The mid-term analysis of primary endpoints will be performed after the dose increment phase is determined by MTD. In addition, after the end of the main observation period of the last patient in the dose increment phase, the parameters of PK were analyzed in the medium term, and the sampling time of subsequent PK might be adjusted. The final analysis is carried out at the end of the experiment.

13. Test Quality Control

In order to ensure the accuracy, consistency, integrity and reliability of the test data generated under this test plan, the research should be carried out in accordance with the SOPs, GCP guidelines and CFDA regulations.

The trial will be monitored by clinical monitors from bidders or their designated units. Field follow-up will be conducted before the start-up test and at appropriate time points during the implementation. Communication records will also include telephone meetings and correspondence.

According to the GCP guidelines, the test inspector must allow access to the original documentation of the researcher in order to check: the consistency of data recorded in CRFs; the safety and protection of patients' rights and interests; whether the test is carried out in accordance with the currently approved test plan and all effective regulatory requirements.

The researcher agreed in writing to cooperate with the relevant inspections in accordance with the requirements of the test plan and to allow authorized personnel to access all documents directly. Materials directly recorded on CRFs and considered as raw materials will be locked before the test is carried out.

Each research base may be inspected by inspectors appointed by the sponsor or by the management. In the event of such inspections/inspections, the researcher should agree to allow inspectors to access the original documents directly and to schedule time for discussion of findings with the relevant staff.

14. Ethnics

14.1 Informed consent

Bidders will provide samples of patients' informed consent, which can be changed by researchers if necessary. The content of the informed consent is shown in Annex 7.

14.2 Signature of informed consent

After fully explaining the purpose, method, expected benefits and potential risks of the trial, the researcher or the person designated by the researcher (if approved by local law) shall be responsible for obtaining written informed consent from each patient participating in the trial. The researcher or designated person must also explain that the patient is free to refuse to participate in the trial or withdraw the informed consent for

any reason at any time. The informed consent should be evaluated and updated if new security information is obtained and significant changes in risk/benefit assessment occur. In these cases, all patients (including those already receiving treatment) should be informed of their new safety information, given copies of their modified forms and reassesses their consent to continue the trial. The signed informed consent must be kept in the patients' file. Copies of IRB/EC-approved informed consent from each base will be kept in the subject's office. Informed consent must be expressed in a language that the patient can read and understand. Researchers must provide subjects with a copy of their informed consent.

14.3 Amendment of informed consent

If significant new safety information about recombinant humanized anti-PD-1 monoclonal antibody injection is obtained during the experiment, the informed consent will be revised accordingly. Copies of the revised patient information manual and informed consent approved by IRB/EC of the research base will be kept at the sponsor's office. After obtaining IRB/EC approval from the research base, each participating and newly recruited trial patient will be required to provide a consent for the latest approved version of the informed consent.

14.4 Institutional Ethics Committee (EC)

The sponsor and the researcher prepare all relevant materials, including the test plan, informed consent, copy of the researcher's manual, the approval and drug test report of the relevant authorities, and any advertisement for the recruited subjects, which are provided to IRB/EC by the researcher. The written approval of ethics shall be submitted by the researcher to Suzhou Zhonghe Biomedical Technology Co., Ltd. The test shall not commence until the researcher has obtained IRB/EC approval for the test scheme and informed consent, and the sponsor has received a copy of the ethical approval letter. According to the IRB/EC regulations of the testing center, all amendments, periodic progress reports and reports of serious adverse events must be submitted to IRB/EC in real time.

14.5 Protection of patients' rights and interests

In order to ensure that the clinical trial is conducted in accordance with ethical requirements, researchers should follow internationally recognized guidelines to obtain scientific knowledge derived from the trial and help to better study diseases when the risk of participants is minimized.

Subjects will receive written informed consent to prove their willingness to participate in the trial. Bidders will provide researchers, institutional ethics committees and patients with updated safety information so that patients can be informed of relevant and constantly updated information that may have an impact on their willingness to continue participating in the trial.

14.6 Compensate

In addition to standard health care for patients, the financial operations of the

additional workload at the trial base will be signed separately. However, all reasonable temporary advances (e.g. transportation costs) incurred by patients participating in the trial may be reimbursed by the sponsor. If the test-related injuries occur, the patients will be compensated and/or treated accordingly.

15. Data Processing and Record Preservation

15.1 Case report form

Data from this trial will be collected in the form of case report form (CRF). CRF filling will be completed and signed by the principal researcher or his authorized representative at each base.

Records of the initial entry and subsequent changes should be retained, including the following information: the time and date of entry, the signature of the person who made the entry or changes.

If the patient fails to complete the trial and withdraws from the trial, the reason for withdrawal must be recorded on the CRF. If the patient withdraws from the trial due to a dose-limiting adverse event, the final results should be recorded as clearly as possible.

Researchers should ensure the accuracy, completeness and real-time nature of the information reported to bidders in CRF and in all required reports.

15.2 Record keeping

The documents to be kept by the researchers are:

- The pilot scheme for signatures and all amendments to date annotations.
- The signature of the researcher's statement.
- Distribution records of all drugs given in the trial, including patient ID and date and quantity of administration.
- IRB/EC approval letters, correspondence records and forms.
- Informed consent of each patient.
- CRFs for each patient.
- Pre-enrollment history and basic information related to CRFs, results of all diagnoses, past treatments and other information about patients' symptoms.
- The detailed medical history of each patient may contain:
    - Medical history during the trial;
    - Recorded results of selection;
- The results of physical examination before treatment;
- Combined or concurrent treatment;
    - Observations of patients' symptoms during the trial;
- Factors that may alter the effects of experimental drugs;
- Follow-up after treatment;
- Copies of test and inspection results required by the test program, including normal laboratory values.
- Copies of interim and final reports sent to IRB or researchers.
- Records of all contacts with medical inspectors and/or designated trial inspectors, including communication content.

- Copies of specific reports on each serious adverse reaction, death or life-threatening problem.

All records should be kept by the researcher until the time required by national regulations (usually five years after the end of the clinical study).In order to avoid possible errors, researchers must contact the sponsor in writing before destroying the test records. Such records shall not be destroyed without prior written authorization from the sponsor. If the test record is accidentally lost or damaged, the researcher shall notify the sponsor in writing.

16 Source Data Management

16.1 Types of raw data

The original data includes all the information in the original records and copies of clinical records of clinical findings, observations or other activities in the clinical trials needed for the organization and evaluation of the trials. Such as:

1) Records of treatment;

2) Inspection inquiry;

3) Laboratory results.

When information is recorded in CRFs, CRFs will be treated as raw data. For this test, CRFs will be the raw material for the following items:

1) In-patient/outpatient records;

2) doctor’s and nurse’s orders;

3) Notebook;

4) Primitive laboratory reports;

5) ECG, EEG, X-ray;

6) Pathological and special evaluation reports;

7) A signed informed consent;

8) Inquiry letters;

9) Patient screening and enrollment records.

16.2 Direct access to raw data/records

In the event of test-related inspections, inspections, IRB/EC assessments, and/or regulatory authorities’ inspection requirements, the researcher/institution shall agree to consult the original materials/documents by the sponsor or its authorized representative.

The Ombudsman shall be responsible for ensuring that:

1) CRF is valid for all data recorded.

2) The safety and rights of patients can be protected.

3) The test is carried out in accordance with the currently approved test scheme, GCP and all the regulations currently in force.

17. Publication Policy

The purpose, content and results of this clinical trial and all future information

must be kept strictly confidential. Copyright of all materials and results shall be owned by the sponsor.

At the end of the experiment or when the test data is sufficient (the bidder has made a reasonable judgement), the researcher can prepare the published data from the experiment. Such information shall be submitted to the sponsor for review and comment prior to the publication of the publication form. In order to ensure that bidders can express their opinions and make relevant recommendations for public dissemination, materials for public dissemination should be submitted to the bidders for review at least 60 days before submission for public publication, public dissemination or review by the public publication committee.

The researcher must agree that all reasonable opinions made by the sponsor relating to the articles to be published by the researcher shall be added to the articles by the researcher.

During the review of the articles to be published publicly, in order to enable the sponsor to take measures to protect their patent information, the sponsor will have the right to postpone the publication of the articles. All materials relating to the test shall not be published publicly without prior written consent of the sponsor. Except for legal reasons, the sponsor or the principal researcher shall not disclose the test results to a third party until a bilateral agreement on data analysis and interpretation has been reached.

18. Revision of test plan and deviation of test plan

18.1 Revision of test plan

If the test plan needs to be changed after approval, a written amendment signed by the same person shall be provided. The ethics committee and CFDA involved in the amendment should be notified for the record. Changes that have a significant impact on the safety of patients in the trial should be approved by the responsible ethical committee and filed with CFDA.

The amendments should be distributed to all persons involved in the pilot. All personnel shall be notified of all process changes required by the amendment.

18.2 Test scheme deviation

Major test program deviations are defined as events or behaviors of patients or researchers who make the main purpose of this trial program unreliable to assess or evaluate results.

The ethics committee shall formulate, record and follow up its procedures, which shall include: no deviation from or alteration of the test plan shall be made before the corresponding amendment is approved in writing, except in cases necessary to eliminate the immediate risk to the subject, or only in respect of the logistical or managerial aspects of the test.

Without the consent of the sponsor and the prior review and written approval of

the amendment by the Ethics Committee, the researcher shall not implement any deviation from the test plan or make any changes to the test plan, except where necessary to eliminate the immediate risk to the test subjects, or only involve changes in the logistical or management aspects of the test (such as the replacement of the inspector, the replacement of the telephone number).Except code. In order to eliminate the immediate risk to the test subjects, the researchers can implement any deviation from the test program or make any changes to the test program without prior access to the ethics committee. Researchers should submit as soon as possible the deviations or changes that have been implemented, the reasons for the deviations or changes, and the proposed revisions to the test plan, if appropriate:

- To be submitted to the Ethics Committee for review and approval;
- Submitted to the sponsor for consent;
- Submit it to the regulatory authorities.

The researcher or the person designated by the researcher shall record and explain all cases of deviation from the approved test plan.

19. Test interruption and termination

19.1 The sponsor asked for interruption of the test

Bidders reserve the right to suspend or permanently suspend a research base or all research bases from conducting this test at any time for various reasons, including, but not limited to, safety or ethical issues or serious violations of relevant requirements.

In exceptional circumstances, the trial may be terminated at a separate test base if the sponsor has reasonable reasons, such as suspecting that the trial was counterfeited or that the trial was not conducted in accordance with the guidelines of the drug clinical trial quality management code.

If the sponsor decides that such action is necessary, the sponsor will discuss the issue with the researcher (including the reasons for such action). Prior to its implementation, the bidder will inform the researchers in advance of the suspension of the experiment.

If the test is suspended or terminated for safety reasons, the sponsor will immediately notify all other researchers and/or institutional organizations conducting the test, and will also notify the regulatory authorities of the suspension or termination of the test and the reasons for it. If the relevant regulations require, the researcher must immediately notify the ethics committee and provide the reasons for suspension or termination.

19.2 End of test

Once the experiment is completed, the inspectors will work with researchers or base staff to carry out the following tasks:

- Return all the test data (except the unnamed original material) to the sponsor.
- Data inquiry;

- Traceability, coordination and disposal of unused research drugs;
- Check the integrity of base test records;
- Send pharmacokinetic/biological samples to the analytical laboratory.

If the test is permanently interrupted, all test data must be returned to the sponsor. In addition, all unused research drugs will be disposed of according to the corresponding test procedures. The financial compensation for researchers and/or institutional organizations will be based on the agreement reached between researchers and bidders.

The sponsor will inform the ethics committee that the trial has been completed within 90 days after the end of the clinical trial. If the test is terminated in advance, the length of time will be reduced to 15 days and the cause of the interruption will be explained clearly.

References

1. Greenwald RJ, Freeman GJ, Sharpe AH. The B7 family revisited. Annu Rev Immunol2005; 23:515-48.
2. Okazaki T, Maeda A, Nishimura H, Kurosaki T, Honjo T. PD-1 immunoreceptor inhibits B cell receptor-mediated signaling by recruiting src homology 2-domaincontaining tyrosine phosphatase 2 to phosphotyrosine. Proc Natl Acad Sci USA2001; 98:13866-71.
3. Thompson RH, Dong H, Lohse CM, Leibovich BC, Blute ML, Cheville JC, et al. PD-1is expressed by tumor-infiltrating immune cells and is associated with poor outcome for patients with renal cell carcinoma. Clin Cancer Res 2007; 13:1757-61.
4. Gao Q, Wang XY, Qiu SJ, Yamato I, Sho M, Nakajima Y, et al. Overexpression of PDL1 significantly associates with tumor aggressiveness and postoperative recurrence inhuman hepatocellular carcinoma. Clin Cancer Res 2009;15:971-9
5. Hamanishi J, Mandai M, Iwasaki M, Okazaki T, Tanaka Y, Yamaguchi K, et al.Programmed cell death 1 ligand 1 and tumor-infiltrating CD8+ T lymphocytes areprognostic factors of human ovarian cancer. Proc Natl Acad Sci USA 2007;104:3360-5
6. Mu CY, Huang JA, Chen Y, Chen C, Zhang XG. High expression of PD-L1 in lungcancer may contribute to poor prognosis and tumor cells immune escape throughsuppressing tumor infiltrating dendritic cells maturation. Med Oncol. 2011; 28:682-8.
7. Liotta F, Gacci M, Frosali F, uerci V, Vittori G, Lapini A, et al. Frequency of regulatoryT cells in peripheral blood and in tumour-infiltrating lymphocytes correlates with poorprognosis in renal cell carcinoma. BJU Intern 2010; 107:1500-

6.
8. Postow MA, Callahan MK, Wolchok JD. Immune Checkpoint Blockade in Cancer Therapy. J Clin Oncol. 2015 Jun 10; 33(17):1974-82.
9. Hodi FS，O’Day SJ，McDermott DF，et al. Improved survival with ipilimumab in patients with metastatic melanoma. N Engl J Med, 2010, 363(8): 711-723.
10. Robert C, Thomas L,Bondarenko I, et al. Ipilimumab plus dacarbazine for previously untreated metastatic melanoma. N Engl J Med, 2011, 364(26): 2517-2526.
11. Robert C，Long GV，Brady B，et al. Nivolumab in previously untreated melanoma without BRAF mutation. N Engl J Med, 2015, 372(4): 320-330.
12. Motzer RJ, Escudier B, McDermott DF, et al. Nivolumab versus Everolimus in Advanced Renal-Cell Carcinoma. N Engl J Med. 2015 Nov 5; 373(19):1803-13.
13. Hamid O, Robert C, Daud A, et al. Safety and tumor responses with lambrolizumab（anti-PD-1）in melanoma. N Engl J Med, 2013, 369(2): 134-144.
14. Robert C，Ribas A，Wolchok JD，et al. Anti- programmed- death- receptor-1 treatment with pembrolizumab in ipilimumabrefractory advanced melanoma：a randomised dose-comparison cohort of a phase 1 trial. Lancet，2014，384（9948）：1109-1117.
15. Rosenberg JE, Hoffman-Censits J, Powles T, et al. Atezolizumab in patients with locally advanced and metastatic urothelial carcinoma who have progressed following treatment with platinum-based chemotherapy: a single-arm, multicentre, phase 2 trial. Lancet. 2016 May 7; 387(10031):1909-20.
16. Rittmeyer A, Barlesi F, Waterkamp D, et al. Atezolizumab versus docetaxel in patients with previously treated non-small-cell lung cancer (OAK): a phase 3, open-label, multicentre randomised controlled trial.
17. Mahoney KM, Rennert PD, Freeman GJ. Combination cancer immunotherapy and new immunomodulatory targets. Nature reviews Drug discovery, 2015, 14(8): 561-84.
18. Larkin J, Chiarion-Sileni V, Gonzalez R, et al. Combined Nivolumab and Ipilimumab or Monotherapy in Untreated Melanoma. The New England journal of medicine, 2015, 373(1): 23-34.
19. Antonia SJ GS, Chow LQM, Juergens RA, Borghaei H, Shen Y, et al. Nivolumab (anti–PD-1; BMS-936558, ONO-4538) and ipilimumab in first-line NSCLC: interim phase I results. J Clin Oncol, 2014, 32(5s): (suppl; abstr 8023).
20. Matthew David Hellmann SNG, Jonathan Wade Goldman et al. CheckMate 012:

Safety and efficacy of first-line (1L) nivolumab (nivo; N) and ipilimumab (ipi; I) in advanced (adv) NSCLC. J Clin Oncol 2016, 34((suppl; abstr 3001).

21. Antonia S, Goldberg SB, Balmanoukian A, et al. Safety and antitumour activity of durvalumab plus tremelimumab in non-small cell lung cancer: a multicentre, phase 1b study. The Lancet Oncology, 2016, 17(3): 299-308.
22. Amita Patnaik MAS, Matthew A. Gubens et al. Phase 1 study of pembrolizumab (pembro; MK-3475) plus ipilimumab (IPI) as second-line therapy for advanced non-small cell lung cancer (NSCLC): KEYNOTE-021 cohort D. J Clin Oncol 2015, 33 (suppl; abstr 8011).
23. Rini BI, Escudier B, Tomczak P, et al. Comparative effectiveness of axitinib versus sorafenib in advanced renal cell carcinoma (AXIS): a randomized phase 3 trial. Lancet, 2011, 378(9807): 1931-1939.
24. Motzer RJ, Escudier B, Tomczak P, et al. Axitinib versus sorafenib as second-line treatment for advanced renal cell carcinoma: overall survival analysis and updated results from a randomised phase 3 trial. Lancet Oncol, 2013, 14(6): 552-562.
25. Qin S, Bi F, Jin J, et al. Axitinib versus sorafenib as a second-line therapy in Asian patients with metastatic renal cell carcinoma: results from a randomized registrational study. Onco Targets Ther, 2015, 8: 1363-1373.
26. Fruehauf J, Lutzky J, McDermott D, et al. Multicenter, phase II study of axitinib, a selective second-generation inhibitor of vascular endothelial growth factor receptors 1, 2, and 3, in patients with metastatic melanoma. Clin Cancer Res. 2011 Dec 1; 17(23):7462-9.

Appendixes

Appendix1： Study Flow Chart

Appendix2: Eastern Cooperative Oncology Group (ECOG) Performance Status

Appendix3: NCI Common Terminology Criteria for Adverse Events version 4.0（NCI CTCAE version 4.0）

Appendix4: Response Evaluation Criteria in Solid Tumors (RECIST1.1)

Appendix5: Immune-Related Response Criteria (irRC)

Appendix6: Content of Informed Consent

Appendix7: Statute of the Data and Safety Monitoring Board

BMJ Publishing Group Limited (BMJ) disclaims all liability and responsibility arising from any reliance placed on this supplemental material which has been supplied by the author(s)

Appendix1：Study Flow Chart

	Screening/baseline period		First treatment cycle				Second treatment cycle				Third and subsequent treatment cycles				Early termination
**Time window**	-28 to 0 days	-14 to 0 days	Week 1-2	Week 3-4	Week 5-6	Week 7-8	Week 1-2	Week 3-4	Week 5-6	Week 7-8	Week 1-2	Week 3-4	Week 5-6	Week 7-8	30 days after treatment Follow-up to 90 days
informed consent	×														
Inclusion and exclusion criteria		×													
Demography, history of disease, history of surgery		×													
History of Cancer Diseases[1]		×													
Tumor evaluation(Tho	×					×				×				×	×

JS001 (V1.2/April 14, 2017) **Protocol Number：HMO-JS001-Ib-CRP-01**

racic, abdominal and pelvic CT or MRI and brain MRI[2])															
Tumor tissue PD-L1 expression [3]	×														×
Comprehensive physical examination		×	×	×[14]	×[14]	×[14]	×[14]	×[14]	×[14]	×[14]	×[14]	×[14]	×[14]	×[14]	×
ECOG score		×	×	×[14]	×[14]	×[14]	×[14]	×[14]	×[14]	×[14]	×[14]	×[14]	×[14]	×[14]	×
Vital signs [4]		×	×	×	×	×	×	×	×	×	×	×	×	×	×
12-lead electrocardiogra m [5]		×	×			×[14]				×[14]				×[14]	×
weight		×	×	×[14]	×[14]	×[14]	×[14]	×[14]	×[14]	×[14]	×[14]	×[14]	×[14]	×[14]	×
height		×													
laboratory examination															
Hematology [6]		×	×[15]	×[14]	×[15]	×[14]	×[15]	×[14]	×[15]	×[14]	×[15]	×[14]	×[15]	×[14]	×

JS001 (V1.2/April 14, 2017) **Protocol Number：HMO-JS001-Ib-CRP-01**

Clinical biochemistry [7]		×	×[15]	×[14]	×[15]	×[14]	×[15]	×[14]	×[15]	×[14]	×[15]	×[14]	×[15]	×[14]	×
Coagulation index [8]		×		×[14]		×[14]		×[14]		×[14]		×[14]		×[14]	×
Urine routine		×	×[15]	×[14]	×[15]	×[14]	×[15]	×[14]	×[15]	×[14]	×[15]	×[14]	×[15]	×[14]	×
Pregnancy test [9]		×													
Virological Indicator [10]		×													×
Thyroid function [11]		×		×[14]		×[14]		×[14]		×[14]		×[14]		×[14]	×
Lymphocyte subsets [12]		×				×[14]				×[14]				×[14]	×
Blood sampling with plasma PK	**See Table 8 for details.**														
Drug Resistant Antibody (ADA)	**See Table 8 for details.**														
Pharmacodynamic Indicators (Receptor Occupancy)	**See Table 8 for details.**														

JS001 (V1.2/April 14, 2017) **Protocol Number：HMO-JS001-Ib-CRP-01**

JS001 treatment		×[16]	×[16]	×[16]	×[16]	×[16]	×[16]	×[16]	×[16]	×[16]	×[16]	×[16]	×[16]	
Acitinib therapy[13]		×	×	×	×	×	×	×	×	×	×	×	×	
Adverse Event Assessment		×												
Combined medication	×													

Remarks：

1. The history of cancer includes the stage of cancer, the date of diagnosis and the means of treatment.
2. At baseline (-28 to 0 days), imaging evaluation of tumors was performed, including head, chest, abdomen and pelvis (if there are other parts, corresponding parts need to be examined); enhanced CT was preferred; if the subjects were allergic to contrast agents, they could use MRI or CT plain scan, bone scan. Tumor evaluation was performed at multiple doses (once every 2 weeks, four times a week), every 2 cycles (+2 weeks) and withdrawal from follow-up (only when the progression of the tumors was uncertain).
3. All subjects should try to collect tumor tissue (preferably fresh tumor samples) for PD-L1 expression detection.
4. Vital signs: including body temperature, heart rate, respiration, blood pressure, multiple drug administration tests: before the first drug administration, every 15 minutes within 1 hour after the beginning of the drug administration, 30 minutes after the end of the drug administration, and every 30 minutes within 1 hour after the end of the drug administration; after the follow-up drug administration, every 30 minutes before, during and after the end of the drug administration: each blood pressure, breath, pulse, body temperature, once, if abnormal, additional measurements are needed. Number of times and prolongation of measurement time to 30 minutes after normal;
5. ECG examinations should be carried out during the screening period (-28 to 0 days), before administration on the first day of each cycle and at the time of withdrawal from follow-up.
6. Blood routine: including whole blood cell count (CBC) and white blood cell (WBC) classification count, red blood cell (RBC) count and platelet count;
7. Blood biochemistry: total protein, albumin, total bilirubin, aspartate aminotransferase (AST), alanine aminotransferase (ALT), alkaline phosphatase (ALP), lactate dehydrogenase (LDH), total cholesterol, HDL, LDL, triglyceride, uric acid, blood urea nitrogen (BUN), creatinine, creatinine clearance rate, electrolyte: sodium (Na+), potassium (K+), chlorine (CI-), calcium, calcium, triglyceride. Phosphorus, amylase, blood sugar and C-reactive protein (CRP).

8. Coagulation function tests: prothrombin time (PT), activated partial thromboplastin time (APTT), fibrinogen (FIB), and international standardized ratio (INR);
9. Pregnancy test: For women of childbearing age, urine (or blood) pregnancy test is necessary.
10. Viral testing can be carried out within 4 weeks before the start of treatment: including total hepatitis B and HBV DNA copy number, hepatitis C, HIV, syphilis;
11. Thyroid function tests: thyroid stimulating hormone (TSH), serum free triiodothyronine (FT3), serum free thyroxine (FT4);
12. Lymphocyte subsets: T lymphocyte subsets (CD3+, CD4+, CD8+), natural killer cells (CD16/56+), immunoglobulin (IgG, IgA, IgM) and complement (C3);
13. Initial treatment was given in each dose group. On the first day, recombinant humanized anti-PD-1 monoclonal antibody injection JS001 was given, and on the second day, axitinib was given orally.
14. Relevant examinations were performed within 3 days before JS001 was given.
15. Choose.
16. JS001 medication window (+2 days)

All assessments will be allowed as unplanned assessments whenever clinical indications are needed during the trial.

Appendix2: Eastern Cooperative Oncology Group (ECOG) Performance Status

Grade	ECOG PERFORMANCE STATUS
0	Fully active, able to carry on all pre-disease performance without restriction (Karnofsky 90-100)
1	Restricted in physically strenuous activity but ambulatory and able to carry out work of a light or sedentary nature, e.g., light house work, office work (Karnofsky 70-80)
2	Ambulatory and capable of all self-care but unable to carry out any work activities; up and about more than 50% of waking hours (Karnofsky 50-60)
3	Capable of only limited self-care; confined to bed or chair more than 50% of waking hours (Karnofsky 30-40)
4	Completely disabled; cannot carry on any self-care; totally confined to bed or chair (Karnofsky 10-20)

Appendix3: NCI Common Terminology Criteria for Adverse Events version 4.0（NCI CTCAE version 4.0）

For toxicity and adverse event reporting, CTC version 4.0 will be used to evaluate toxicity.

Copies of CTCAE version 4.0 can be obtained from the following URL:

http://ctep.cancer.gov/forms/CTCAEv4.pdf.

All adverse events, whether observed by researchers or reported by patients, must be recorded, including details of duration, severity, measures taken to treat and test patients, and final outcomes of patients. Researchers must assess the relationship between each adverse event and the drug tested, as well as the severity of the event.

Appendix4: Response Evaluation Criteria in Solid Tumors (RECIST1.1)

Response Evaluation Criteria in Solid Tumors Version 1.1

As currently there is no formal Chinese version of RICIST1.1 published, this is an internal translation version, and for more details, please refer to the English version at http://ctep.cancer.gov/protocolDevelopment/docs/recist_guideline.pdf.

Abstract

Background

Assessment of the change in tumor burden is an important feature of the clinical evaluation of cancer therapeutics: both tumor shrinkage (objective response) and disease progression are useful endpoints in clinical trials. Since RECIST was published in 2000, many investigators, cooperative groups, industry and government authorities have adopted these criteria in the assessment of treatment outcomes. However, a number of questions and issues have arisen which have led to the development of a revised RECIST guideline (version 1.1). Evidence for changes, summarized in separate papers in this special issue, has come from assessment of a large data warehouse (>6500 patients), simulation studies and literature reviews.

Highlights of revised RECIST 1.1

Major changes include:

Number of lesions to be assessed: based on evidence from numerous trial databases merged into a data warehouse for analysis purposes, the number of lesions required to assess tumor burden for response determination has been reduced from a maximum of 10 to a maximum of five total (and from five to two per organ, maximum).

Assessment of pathological lymph nodes is now incorporated: nodes with a short axis of P15 mm are considered measurable and assessable as target lesions. The short axis measurement should be included in the sum of lesions in calculation of tumor response. Nodes that shrink to <10 mm short axis are considered normal.

Confirmation of response is required for trials with response primary endpoint but is no longer required in randomized studies since the control arm serves as appropriate means of interpretation of data. Disease progression is clarified in several aspects: in addition to the previous definition of progression in target disease of 20% increase in sum, a 5 mm absolute increase is now required as well to guard against over calling PD when the total sum is very small. Furthermore, there is guidance offered on what constitutes ‘unequivocal progression’ of non-measurable/non-target disease, a source of confusion in the original RECIST guideline. Finally, a section on measurement of

new lesions, including the interpretation of FDG-PET scan assessment is included. Imaging guidance: the revised RECIST includes a new imaging appendix with updated recommendations on the optimal anatomical assessment of lesions.

Future work: A key question considered by the RECIST Working Group in developing RECIST 1.1 was whether it was appropriate to move from anatomic unidimensional assessment of tumor burden to either volumetric anatomical assessment or to functional assessment with PET or MRI. It was concluded that, at present, there is not sufficient standardization or evidence to abandon anatomical assessment of tumor burden. The only exception to this is in the use of FDG-PET imaging as an adjunct to determination of progression. As is detailed in the final paper in this special issue, the use of these promising newer approaches requires appropriate clinical validation studies.

Keywords: Response criteria Solid tumors Guidelines

1. Background

1.1. History of RECIST criteria

Assessment of the change in tumor burden is an important feature of the clinical evaluation of cancer therapeutics. Both tumor shrinkage (objective response) and time to the development of disease progression are important endpoints in cancer clinical trials. The use of tumor regression as the endpoint for phase II trials screening new agents for evidence of anti-tumor effect is supported by years of evidence suggesting that, for many solid tumors, agents which produce tumor shrinkage in a proportion of patients have a reasonable (albeit imperfect) chance of subsequently demonstrating an improvement in overall survival or other time to event measures in randomized phase III studies. At present, objective response carries with it a body of evidence greater than for any other biomarker supporting its utility as a measure of promising treatment effect in phase II screening trials. Furthermore, at both the phase II and phase III stage of drug development, clinical trials in advanced disease settings are increasingly utilizing time to progression (or progression-free survival) as an endpoint upon which efficacy conclusions are drawn, which is also based on anatomical measurement of tumor size.

However, both of these tumor endpoints, objective response and time to disease progression, are useful only if based on widely accepted and readily applied standard criteria based on anatomical tumor burden. In 1981, the World Health Organization (WHO) first published tumor response criteria, mainly for use in trials where tumor response was the primary endpoint. The WHO criteria introduced the concept of an overall assessment of tumor burden by summing the products of dimensional lesion measurements and determined response to therapy by evaluation of change from

baseline while on treatment. However, in the decades that followed their publication, cooperative groups and pharmaceutical companies that used the WHO criteria often 'modified' them to accommodate new technologies or to address areas that were unclear in the original document. This led to confusion in interpretation of trial results and in fact, the application of varying response criteria was shown to lead to very different conclusions about the efficacy of the same regimen. In response to these problems, an International Working Party was formed in the mid-1990s to standardize and simplify response criteria. New criteria, known as RECIST (Response Evaluation Criteria in Solid Tumors), were published in 2000. Key features of the original RECIST include definitions of minimum size of measurable lesions, instructions on how many lesions to follow (up to 10; a maximum five per organ site), and the use of unidimensional, rather than dimensional, measures for overall evaluation of tumor burden. These criteria have subsequently been widely adopted by academic institutions, cooperative groups, and industry for trials where the primary endpoints are objective response or progression. In addition, regulatory authorities accept RECIST as an appropriate guideline for these assessments.

2. Purpose of this guideline

This guideline describes a standard approach to solid tumor measurement and definitions for objective assessment of change in tumor size for use in adult and pediatric cancer clinical trials. It is expected these criteria will be useful in all trials where objective response is the primary study endpoint, as well as in trials where assessment of stable disease, tumor progression or time to progression analyses are undertaken, since all of these outcome measures are based on an assessment of anatomical tumor burden and its change on study. There are no assumptions in this paper about the proportion of patients meeting the criteria for any of these endpoints which will signal that an agent or treatment regimen is active: those definitions are dependent on type of cancer in which a trial is being undertaken and the specific agent(s) under study. Protocols must include appropriate statistical sections which define the efficacy parameters upon which the trial sample size and decision criteria are based. In addition to providing definitions and criteria for assessment of tumor response, this guideline also makes recommendations regarding standard reporting of the results of trials that utilize tumor response as an endpoint.

While these guidelines may be applied in malignant brain tumor studies, there are also separate criteria published for response assessment in that setting. This guideline

is not intended for use for studies of malignant lymphoma since international guidelines for response assessment in lymphoma are published separately.

Finally, many oncologists in their daily clinical practice follow their patients' malignant disease by means of repeated imaging studies and make decisions about continued therapy based on both objective and symptomatic criteria. It is not intended that these RECIST guidelines play a role in that decision, except if determined appropriate by the treating oncologist.

3. Measurability of tumor at baseline

3.1. Definitions

At baseline, tumor lesions/lymph nodes will be categorized measurable or non-measurable as follows:

3.1.1. Measurable

Tumor lesions: Must be accurately measured in at least one dimension (longest diameter in the plane of measurement is to be recorded) with a minimum size of:

• 10 mm by CT scan (CT scan slice thickness no greater than 5 mm; see Appendix II on imaging guidance).

• 10 mm caliper measurement by clinical exam (lesions which cannot be accurately measured with calipers should be recorded as non-measurable).

• 20 mm by chest X-ray.

Malignant lymph nodes: To be considered pathologically enlarged and measurable, a lymph node must be ≥15 mm in short axis when assessed by CT scan (CT scan slice thickness recommended to be no greater than 5 mm). At baseline and in follow-up, only the short axis will be measured and followed. See also notes below on 'Baseline documentation of target and non-target lesions' for information on lymph node measurement.

3.1.2. Non-measurable

All other lesions, including small lesions (longest diameter <10 mm or pathological lymph nodes with ≥10 to <15 mm short axis) as well as truly non-measurable lesions. Lesions considered truly non-measurable include leptomeningeal disease, ascites, pleural or pericardial effusion, inflammatory breast disease, lymphatic involvement of skin or lung and abdominal masses/abdominal organomegaly identified by physical exam that is not measurable by reproducible imaging techniques.

3.1.3. Special considerations regarding lesion measurability

Bone lesions, cystic lesions, and lesions previously treated with local therapy require particular comment:

Bone lesions:

• Bone scan, PET scan or plain films are not considered adequate imaging techniques to measure bone lesions. However, these techniques can be used to confirm the presence or disappearance of bone lesions.

• Lytic bone lesions or mixed acute lytic lesions, with identifiable soft tissue components, that can be evaluated by cross sectional imaging techniques such as CT or MRI can be considered as measurable lesions if the soft tissue component meets the definition of measurability described above.

• Osteogenesis lesions are non-measurable.

Cystic lesions:

• Lesions that meet the criteria for radiographically defined simple cysts should not be considered as malignant lesions (neither measurable nor non-measurable) since they are, by definition, simple cysts.

• 'Cystic lesions' thought to represent cystic metastases can be considered as measurable lesions, if they meet the definition of measurability described above. However, if non-cystic lesions are present in the same patient, these are preferred for selection as target lesions.

Lesions with prior local treatment

•Tumor lesions situated in a previously irradiated area, or in an area subjected to other loco-regional therapy, are usually not considered measurable unless there has been demonstrated progression in the lesion. Study protocols should detail the conditions under which such lesions would be considered measurable.

3.2. Specifications by methods of measurements

3.2.1. Measurement of lesions

All measurements should be recorded in metric notation, using calipers if clinically assessed. All baseline evaluations should be performed as close as possible to the treatment start and never more than 4 weeks before the beginning of the treatment.

3.2.2. Method of assessment

The same method of assessment and the same technique should be used to characterize each identified and reported lesion at baseline and during follow-up. Imaging based evaluation should always be done rather than clinical examination unless the lesion(s) being followed cannot be imaged but are assessable by clinical exam.

Clinical lesions: Clinical lesions will only be considered measurable when they are superficial and ≥10 mm diameter as assessed using calipers (e.g. skin nodules). For the case of skin lesions, documentation by color photography including a ruler to

estimate the size of the lesion is suggested. As noted above, when lesions can be evaluated by both clinical exam and imaging, imaging evaluation should be undertaken since it is more objective and may also be reviewed at the end of the study.

Chest X-ray: Chest CT is preferred over chest X-ray, particularly when progression is an important endpoint, since CT is more sensitive than X-ray, particularly in identifying new lesions. However, lesions on chest X-ray may be considered measurable if they are clearly defined and surrounded by aerated lung. See Appendix II for more details.

CT, MRI: CT is the best currently available and reproducible method to measure lesions selected for response assessment. This guideline has defined measurability of lesions on CT scan based on the assumption that CT slice thickness is 5 mm or less. As is described in Appendix II, when CT scans have slice thickness greater than 5 mm, the minimum size for a measurable lesion should be twice the slice thickness. MRI is also acceptable in certain situations (e.g. for body scans). More details concerning the use of both CT and MRI for assessment of objective tumor response evaluation are provided in Appendix II.

Ultrasound: Ultrasound is not useful in assessment of lesion size and should not be used as a method of measurement. Ultrasound examinations cannot be reproduced in their entirety for independent review later and, because they are operator dependent, it cannot be guaranteed that the same technique and measurements will be taken from one assessment to the next (described in greater detail in Appendix II). If new lesions are identified by ultrasound in the course of the study, confirmation by CT or MRI is advised. If there is concern about radiation exposure at CT, MRI may be used instead of CT in selected instances.

Endoscopy, laparoscopy: The utilization of these techniques for objective tumor evaluation is not advised. However, they can be useful to confirm complete pathological response when biopsies are obtained or to determine relapse in trials where recurrence following complete response or surgical resection is an endpoint.

Tumor markers: Tumor markers alone cannot be used to assess objective tumor response. If markers are initially above the upper normal limit, however, they must normalize for a patient to be considered in complete response. Because tumor markers are disease specific, instructions for their measurement should be incorporated into protocols on a disease specific basis. Specific guidelines for both CA-125 response (in recurrent ovarian cancer) and PSA response (in recurrent prostate cancer), have been published. In addition, the Gynecologic Cancer Intergroup has developed CA125

progression criteria which are to be integrated with objective tumor assessment for use in first-line trials in ovarian cancer.

Cytology, histology: These techniques can be used to differentiate between PR and CR in rare cases if required by protocol (for example, residual lesions in tumor types such as germ cell tumors, where known residual benign tumors can remain). When effusions are known to be a potential adverse effect of treatment (e.g. with certain taxane compounds or angiogenesis inhibitors), the cytological confirmation of the neoplastic origin of any effusion that appears or worsens during treatment can be considered if the measurable tumor has met criteria for response or stable disease in order to differentiate between response (or stable disease) and progressive disease.

4. Tumor response evaluation

4.1. Assessment of overall tumor burden and measurable disease

To assess objective response or future progression, it is necessary to estimate the overall tumor burden at baseline and use this as a comparator for subsequent measurements. Only patients with measurable disease at baseline should be included in protocols where objective tumor response is the primary endpoint. Measurable disease is defined by the presence of at least one measurable lesion. In studies where the primary endpoint is tumor progression (either time to progression or proportion with progression at a fixed date), the protocol must specify if entry is restricted to those with measurable disease or whether patients having non-measurable disease only are also eligible.

4.2. Baseline documentation of ‘target’ and ‘non-target’ lesions

When more than one measurable lesion is present at baseline all lesions up to a maximum of five lesions total (and a maximum of two lesions per organ) representative of all involved organs should be identified as target lesions and will be recorded and measured at baseline (this means in instances where patients have only one or two organ sites involved a maximum of two and four lesions respectively will be recorded).

Target lesions should be selected based on their size (lesions with the longest diameter), be representative of all involved organs, but in addition should be those that lend themselves to reproducible repeated measurements. It may be the case that, on occasion, the largest lesion does not lend itself to reproducible measurement in which circumstance the next largest lesion which can be measured reproducibly should be selected.

Lymph nodes merit special mention since they are normal anatomical structures which may be visible by imaging even if not involved by tumor. Pathological nodes

which are defined as measurable and may be identified as target lesions must meet the criterion of a short axis of ≥ 15 mm by CT scan. Only the short axis of these nodes will contribute to the baseline sum. The short axis of the node is the diameter normally used by radiologists to judge if a node is involved by solid tumor. Nodal size is normally reported as two dimensions in the plane in which the image is obtained (for CT scan this is almost the axial plane; for MRI the plane of acquisition may be axial, sagittal or coronal). The smaller of these measures is the short axis. For example, an abdominal node which is reported as being 20 mm × 30 mm has a short axis of 20 mm and qualifies as a malignant, measurable node. In this example, 20 mm should be recorded as the node measurement. All other pathological nodes (those with short axis ≥ 10 mm but <15 mm) should be considered non-target lesions. Nodes that have a short axis <10 mm are considered non-pathological and should not be recorded or followed.

A sum of the diameters (longest for non-nodal lesions, short axis for nodal lesions) for all target lesions will be calculated and reported as the baseline sum diameters. If lymph nodes are to be included in the sum, then as noted above, only the short axis is added into the sum. The baseline sum diameters will be used as reference to further characterize any objective tumor regression in the measurable dimension of the disease.

All other lesions include pathological lymph nodes should be identified as non-target lesions and should also be recorded at baseline. Measurements are not required, and these lesions should be followed as ‘present’, ‘absent’, or in rare cases ‘unequivocal progression’. In addition, it is possible to record multiple non-target lesions involving the same organ as a single item on the case record form (e.g. ‘multiple enlarged pelvic lymph nodes’ or ‘multiple liver metastases’).

4.3. Response criteria

This section provides the definitions of the criteria used to determine objective tumor response for target lesions.

4.3.1. Evaluation of target lesions

Complete Response (CR): Disappearance of all target lesions. Any pathological lymph nodes (whether target or non-target) must have reduction in short axis to<10 mm.

Partial Response (PR): At least a 30% decrease in the sum of diameters of target lesions, taking as reference the baseline sum diameters.

Progressive Disease (PD): At least a 20% increase in the sum of diameters of target lesions, taking as reference the smallest sum on study (this includes the baseline sum if that is the smallest on study). In addition to the relative increase of 20%, the sum must

also demonstrate an absolute increase of at least 5 mm. (Note: the appearance of one or more new lesions is also considered progression).

Stable Disease (SD): Neither sufficient shrinkage to qualify for PR nor sufficient increase to qualify for PD, taking as reference the smallest sum diameters while on study.

4.3.2. Special notes on t

he assessment of target lesions

Lymph nodes:

Lymph nodes identified as target lesions should always have the actual short axis measurement recorded (measured in the same anatomical plane as the baseline examination), even if the nodes regress to below 10 mm on study. This means that when lymph nodes are included as target lesions, the 'sum' of lesions may not be zero even if complete response criteria are met, since a normal lymph node is defined as having a short axis of < 10 mm. Case report forms or other data collection methods may therefore be designed to have target nodal lesions recorded in a separate section where, in order to qualify for CR, each node must achieve a short axis <10 mm. For PR, SD and PD, the actual short axis measurement of the nodes is to be included in the sum of target lesions.

Target lesions that become 'too small to measure':

While on study, all lesions (nodal and non-nodal) recorded at baseline should have their actual measurements recorded at each subsequent evaluation, even when very small (e.g. 2 mm).

However, sometimes lesions or lymph nodes which are recorded as target lesions at baseline become so faint on CT scan that the radiologist may not feel comfortable assigning an exact measure and may report them as being 'too small to measure'. When this occurs, it is important that a value be recorded on the case report form. If it is the opinion of the radiologist that the lesion has likely disappeared, the measurement should be recorded as 0 mm. If the lesion is believed to be present and is faintly seen but too small to measure, a default value of 5 mm should be assigned (Note: It is less likely that this rule will be used for lymph nodes since they usually have a definable size when normal and are frequently surrounded by fat such as in the retroperitoneum; however, if a lymph node is believed to be present and is faintly seen but too small to measure, a default value of 5 mm should be assigned in this circumstance as well).

This default value is derived from the 5 mm CT slice thickness (but should not be changed with varying CT slice thickness). The measurement of these lesions is

potentially non-reproducible; therefore, providing this default value will prevent false responses or progressions based upon measurement error. To reiterate, however, if the radiologist is able to provide an actual measure, that should be recorded, even if it is below 5 mm.

Lesions that split or coalesce on treatment:

As noted in Appendix II, when non-nodal lesions 'fragment', the longest diameters of the fragmented portions should be added together to calculate the target lesion sum. Similarly, as lesions coalesce, a plane between them may be maintained that would aid in obtaining maximal diameter measurements of each individual lesion. If the lesions have truly coalesced such that they are no longer separable, the vector of the longest diameter in this instance should be the maximal longest diameter for the 'coalesced lesion'.

4.3.3. Evaluation of non-target lesions

This section provides the definitions of the criteria used to determine the tumor response for the group of non-target lesions. While some non-target lesions may actually be measurable, they need not be measured and instead should be assessed only qualitatively at the time points specified in the protocol.

Complete Response (CR): Disappearance of all non-target lesions and normalization of tumor marker level. All lymph nodes must be non-pathological in size (<10 mm short axis).

Non-CR/Non-PD: Persistence of one or more non-target lesion(s) and/or maintenance of tumor marker level above the normal limits.

Progressive Disease (PD): Unequivocal progression (see comments below) of existing non-target lesions. (Note: the appearance of one or more new lesions is also considered progression).

4.3.4. Special notes on assessment of progression of non-target disease

The concept of progression of non-target disease requires additional explanation as follows: When the patient also has measurable disease. In this setting, to achieve 'unequivocal progression' on the basis of the non-target disease, there must be an overall level of substantial worsening in non-target disease such that, even in presence of SD or PR in target disease, the overall tumor burden has increased sufficiently to merit discontinuation of therapy. A modest 'increase' in the size of one or more non-target lesions is usually not sufficient to quality for unequivocal progression status. The designation of overall progression solely based on change in non-target disease in the face of SD or PR of target disease will therefore be extremely rare.

For the patient only having the non-measurable disease, this circumstance arises in some phase III trials when it is not a criterion of study entry to have measurable disease. The same general concepts apply here as noted above, however, in this instance there is no measurable disease assessment to factor into the interpretation of an increase in non-measurable disease burden. Because worsening in non-target disease cannot be easily quantified (by definition: if all lesions are truly non-measurable) a useful test that can be applied when assessing patients for unequivocal progression is to consider if the increase in overall disease burden based on the change in non-measurable disease is comparable in magnitude to the increase that would be required to declare PD for measurable disease: i.e. an increase in tumor burden representing an additional 73% increase in ‘volume’ (which is equivalent to a 20% increase diameter in a measurable lesion). Examples include an increase in a pleural effusion from ‘trace’ to ‘large’, an increase in lymphangitic disease from localized to widespread, or may be described in protocols as ‘sufficient to require a change in therapy’. If ‘unequivocal progression’ is seen, the patient should be considered to have had overall PD at that point. While it would be ideal to have objective criteria to apply to non-measurable disease, the nature of that disease makes it impossible; therefore, the increase must be substantial.

4.3.5. New lesions

The appearance of new malignant lesions denotes disease progression; therefore, some comments on measurement of new lesions are important. There are no specific criteria for the identification of new radiographic lesions; however, the finding of a new lesion should be unequivocal: i.e. not attributable to differences in scanning technique, change in imaging modality or findings thought to represent something other than tumor (for example, some ‘new’ bone lesions may be simply healing or flare of pre-existing lesions). This is particularly important when the patient’s baseline lesions show partial or complete response. For example, necrosis of a liver lesion may be reported on a CT scan report as a ‘new’ cystic lesion, which it is not.

A lesion identified on a follow-up study in an anatomical location that was not scanned at baseline is considered a new lesion and will indicate disease progression. An example of this is the patient who has visceral disease at baseline and while on study has a CT or MRI brain ordered which reveals metastases. The patient’s brain metastases are considered to be evidence of PD even if he/she did not have brain imaging at baseline.

If a new lesion is equivocal, for example because of its small size, continued therapy and follow-up evaluation will clarify if it represents truly new disease. If repeat

scans confirm there is definitely a new lesion, then progression should be declared using the date of the initial scan.

While FDG-PET response assessments need additional study, it is sometimes reasonable to incorporate the use of FDG-PET scanning to complement CT scanning in assessment of progression (particularly possible 'new' disease). New lesions based on FDG-PET imaging can be identified according to the following algorithm:

Negative FDG-PET at baseline, with a positive FDG-PET at follow-up is a sign of PD based on a new lesion.

No FDG-PET at baseline and a positive FDG-PET at follow-up:

If the positive FDG-PET at follow-up corresponds to anew site of disease confirmed by CT, this is PD.

If the positive FDG-PET at follow-up is not confirmed as a new site of disease on CT, additional follow-up CT scans are needed to determine if there is truly progression occurring at that site (if so, the date of PD will be the date of the initial abnormal FDG-PET scan).

If the positive FDG-PET at follow-up corresponds to a pre-existing site of disease on CT that is not progressing based on the anatomic images, this is not PD.

4.4. Evaluation of best overall response

The best overall response is the best response recorded from the start of the study treatment until the end of treatment taking into account any requirement for confirmation. On occasion, a response may not be documented until after the end of therapy so protocols should be clear if post-treatment assessments are to be considered in determination of best overall response. Protocols must specify how any new therapy introduced before progression will affect best response designation. The patient's best overall response assignment will depend on the findings of both target and non-target disease and will also take into consideration the appearance of new lesions. Furthermore, depending on the nature of the study and the protocol requirements, it may also require confirmatory measurement. Specifically, in non-randomized trials where response is the primary endpoint, confirmation of PR or CR is needed to deem either one the 'best overall response'. This will be described further below.

4.4.1. Time point response

It is assumed that at each protocol specified time point, a response assessment occurs. Table 1 on the next page provides a summary of the overall response status calculation at each time point for patients who have measurable disease at baseline.

When patients have non-measurable (therefore non-target) disease only, Table 2 is to be used.

4.4.2. Missing assessments and unevaluable designation

When no imaging/measurement is done at all at a particular time point, the patient is not evaluable (NE) at that time point. If only a subset of lesion measurements are made at an assessment, usually the case is also considered NE at that time point, unless a convincing argument can be made that the contribution of the individual missing lesion(s) would not change the assigned time point response. This would be most likely to happen in the case of PD. For example, if a patient had a baseline sum of 50 mm with three measured lesions and at follow-up only two lesions were assessed, but those gave a sum of 80 mm, the patient will have achieved PD status, regardless of the contribution of the missing lesion.

4.4.3. Best overall response: all time points

The best overall response is determined once all the data for the patient is known.

Best response determination in trials where confirmation of complete or partial response IS NOT required: Best response in these trials is defined as the best response across all time points (for example, a patient who has SD at first assessment, PR at second assessment, and PD on last assessment has a best overall response of PR). When SD is believed to be best response, it must also meet the protocol specified minimum time from baseline. If the minimum time does not met when SD is otherwise the best time point response, the patient's best response depends on the subsequent assessments. For example, a patient who has SD at first assessment, PD at second and does not meet minimum duration for SD, will have a best response of PD. The same patient lost to follow-up after the first SD assessment would be considered unevaluable.

Best response determination in trials where confirmation of complete or partial response IS required: Complete or partial responses may be claimed only if the criteria for each are met at a subsequent time point as specified in the protocol (generally 4 weeks later). In this circumstance, the best overall response can be interpreted as in Table 3.

4.4.4. Special notes on response assessment

When nodal disease is included in the sum of target lesions and the nodes decrease to 'normal' size (<10 mm), they may still have a measurement reported on scans. This measurement should be recorded even though the nodes are normal in order not to overstate progression should it be based on increase in size of the nodes. As noted earlier,

this means that patients with CR may not have a total sum of ‘zero’ on the case report form (CRF).

In trials where confirmation of response is required, repeated ‘NE’ time point assessments may complicate best response determination. The analysis plan for the trial must address how missing data/assessments will be addressed in determination of response and progression. For example, in most trials it is reasonable to consider a patient with time point responses of PR-NE-PR as a confirmed response.

Patients with a global deterioration of health status requiring discontinuation of treatment without objective evidence of disease progression at that time should be reported as ‘symptomatic deterioration’. Every effort should be made to document objective progression even after discontinuation of treatment. Symptomatic deterioration is not a descriptor of an objective response: it is a reason for stopping study therapy. The objective response status of such patients is to be determined by evaluation of target and non-target disease as shown in Tables 1–3.

Conditions that define ‘early progression, early death and inevaluability’ are study specific and should be clearly described in each protocol (depending on treatment duration, treatment periodicity).

In some circumstances, it may be difficult to distinguish residual disease from normal tissue. When the evaluation of complete response depends upon this determination, it is recommended that the residual lesion be investigated (fine needle aspirate/biopsy) before assigning a status of complete response. FDG-PET may be used to upgrade a response to a CR in a manner similar to a biopsy in cases where a residual radiographic abnormality is thought to represent fibrosis or scarring. The use of FDG-PET in this circumstance should be prospectively described in the protocol and supported by disease specific medical literature for the indication. However, it must acknowledge that both approaches may lead to false positive CR due to limitations of FDG-PET and biopsy resolution/sensitivity.

Table 1 Time Point Response: Patients with Target (+/- non-target) Disease

Target Lesions	Non-target Lesions	New Lesions	Overall Response
CR	CR	No	CR
CR	Non-CR / Non-PD	No	PR
CR	Not evaluated	No	PR
PR	Non-PD or not all evaluated	No	PR
SD	Non-PD or not all	No	SD

	evaluated		
Not all evaluated	Non-PD	No	NE
PD	Any	Yes or No	PD
Any	PD	Yes or No	PD
Any	Any	Yes	PD
CR = complete response	PR = partial response	SD = stable disease	PD = progressive disease<br>NE = unevaluable

Table 2 Time Point Response - Patients with Non-target Disease Only

Non-target Lesions	New Lesions	Overall Response
CR	No	CR
Non-CR/Non-PD	No	Non-CR/Non-PD
Not all evaluated	No	NE
Unequivocal PD	Yes or No	PD
Any	Yes	PD

Note: ‘Non-CR/non-PD’ is preferred over ‘stable disease’ for non-target disease since SD is increasingly used as endpoint for assessment of efficacy in some trials so to assign this category when no lesions can be measured is not advised.

For equivocal findings of progression (e.g. very small and uncertain new lesions; cystic changes or necrosis in existing lesions), treatment may continue until the next scheduled assessment. If at the next scheduled assessment, the progression is confirmed, the date of progression should be the earlier date when progression was suspected.

Table 3 Best Overall Response When confirmation of CR and PR Required

Overall Response at First Time point	Overall Response at Subsequent Time point	Best Overall Response
CR	CR	CR
CR	PR	SD, PD or PR[a]
CR	SD	SD provided minimum criteria for SD duration met, otherwise, PD
CR	PD	SD provided minimum criteria for SD duration met, otherwise, PD
CR	NE	SD provided minimum criteria for

		SD duration met, otherwise NE
PR	CR	PR
PR	PR	PR
PR	SD	SD
PR	PD	SD provided minimum criteria for SD duration met, otherwise, PD
PR	NE	SD provided minimum criteria for SD duration met, otherwise NE
NE	NE	NE

CR = complete response, PR = partial response, SD = stable disease, PD = progressive disease, and NE = unevaluable.

[a] If a CR is truly met at first time point, then any disease seen at a subsequent time point, even disease meeting PR criteria relative to baseline, makes the disease PD at that point (since disease must have reappeared after CR). Best response would depend on whether minimum duration for SD was met. However, sometimes ‘CR’ may be claimed when subsequent scans suggest small lesions were likely still present. In fact, the patient had PR, not CR at the first time point. Under these circumstances, the original CR should be changed to PR and the best response is PR.

4.5. Frequency of tumor re-evaluation

Frequency of tumor re-evaluation while on treatment should be protocol specific and adapted to the type and schedule of treatment. However, in the context of phase II studies where the beneficial effect of therapy is not known, follow-up every 6–8 weeks (timed to coincide with the end of a cycle) is reasonable. Smaller or greater time intervals than these could be justified in specific regimens or circumstances. The protocol should specify which organ sites are to be evaluated at baseline (usually those most likely to be involved with metastatic disease for the tumor type under study) and how often evaluations are repeated. Normally, all target and non-target sites are evaluated at each assessment. In the selected circumstance, certain non-target organs may be evaluated less frequently. For example, bone scans may need to be repeated only when complete response is identified in target disease or when progression in bone is suspected.

After the end of the treatment, the need for repetitive tumor evaluations depends on whether the trial has as a goal the response rate or the time to an event (progression/death). If ‘time to an event’ (e.g. time to progression, disease-free survival, progression-free survival) is the main endpoint of the study, then routine scheduled re-

evaluation of protocol specified sites of disease is warranted. In randomized comparative trials in particular, the scheduled assessments should be performed as identified on a calendar schedule (for example: every 6–8 weeks on treatment or every 3–4 months after treatment) and should not be affected by delays in therapy, drug holidays or any other events that might lead to imbalance in a treatment arm in the timing of disease assessment.

4.6. Confirmatory measurement/duration of response

4.6.1. Confirmation

In non-randomized trials where response is the primary endpoint, confirmation of PR and CR is required to ensure responses identified are not the result of measurement error. This will also permit appropriate interpretation of results in the context of historical data where response has traditionally required confirmation in such trials However, in all other circumstances, i.e. in randomized trials (phase II or III) or studies where stable disease or progression are the primary endpoints, confirmation of response is not required since it will not add value to the interpretation of trial results. However, elimination of the requirement for response confirmation may increase the importance of central review to protect against bias, in particular in studies which are not blinded.

In the case of SD, measurements must have met the SD criteria at least once after study entry at a minimum interval (in general not less than 6–8 weeks) that is defined in the study protocol.

4.6.2. Duration of overall response

The duration of overall response is measured from the time measurement criteria are first met for CR/PR (whichever is first recorded) until the first date that recurrent or progressive disease is objectively documented (taking as reference for progressive disease the smallest measurements recorded on study). The duration of overall complete response is measured from the time measurement criteria are first met for CR until the first date that recurrent disease is objectively documented.

4.6.3. Duration of stable disease

Stable disease is measured from the start of the treatment (in randomized trials, from date of randomization) until the criteria for progression are met, taking as reference the smallest sum on study (if the baseline sum is the smallest, this is the reference for calculation of PD). The clinical relevance of the duration of stable disease varies in different studies and diseases. In a particular trial, if the proportion of patients maintaining stable disease with the minimum duration were as the endpoint, the

protocol should specify the minimal time interval required between two measurements for determination of stable disease.

Note: The duration of response and stable disease as well as the progression-free survival are influenced by the frequency of follow-up after baseline evaluation. It is not in the scope of this guideline to define a standard follow-up frequency. The frequency should take into account many parameters including disease types and stages, treatment periodicity and standard practice. However, these limitations of the precision of the measured endpoint should be taken into account if comparisons between trials are to be made.

4.7. Progression-free survival/proportion progression-free

4.7.1. Phase II trials

This guideline focuses primarily on the use of objective response endpoints for phase II trials. In some circumstances, ‘response rate’ may not be the optimal method to assess the potential anticancer activity of new agents/regimens. In such cases ‘progression-free survival’ (PFS) or the ‘proportion progression-free’ at landmark time points, might be considered appropriate alternatives to provide an initial signal of biologic effect of new agents. It is clear, however, that in an uncontrolled trial, these measures are subject to criticism since an apparently promising observation may be related to biological factors such as patient selection and not the impact of the intervention. Thus, phase II screening trials utilizing these endpoints are best designed with a randomized control. Exceptions may exist where the behavior patterns of certain cancers are so consistent (and usually consistently poor), that a non-randomized trial is justifiable. However, in these cases it will be essential to document with care the basis for estimating the expected PFS or proportion progression-free in the absence of a treatment effect.

The following contents are Phase III Evaluation Endpoints, Individual Evaluation, Results Reports, etc. See English version for details.

Appendix5: Immune-Related Response Criteria (irRC)

The content and characteristics of the new immunotherapy efficacy evaluation criteria:

1. **Antitumor response based on total measurable tumor burden**

Antitumor response based on total measurable tumor burden. For the irRC, only index and measurable new lesions are taken into account (in contrast to conventional WHO criteria, which do not require the measurement of new lesions, nor do they include new lesion measurements in the characterization of evolving tumor burden). At the baseline tumor assessment, the sum of the products of the two largest perpendicular diameters (SPD) of all index lesions (five lesions per organ, up to 10 visceral lesions and five cutaneous index lesions) is calculated. At each subsequent tumor assessment, the SPD of the index lesions and of new, measurable lesions (⩾5 × 5 mm; up to 5 new lesions per organ: 5 new cutaneous lesions and 10 visceral lesions) are added together to provide the total tumor burden:

$$\text{Tumor Burden} = \text{SPD}_{\text{index lesions}} + \text{SPD}_{\text{new, measurable lesions}}$$

A comparison of the use of SPD in WHO criteria versus the use of tumor burden in irRC is presented in Table 1.

2. **Time-point response assessment using irRC.**

The response evaluation of iRECIST refers to the comparison of overall tumor burden and baseline tumor burden to observe how much the increase/decrease of tumor burden is. Investigators should classify the response by 2 observational points in a row, whose time interval should be more than 4 weeks: irCR—all the lesions vanished completely; irPR—tumor burden reduction ⩾30% compared with baseline; irSD—not met definition of irCR or irPR without irPD; irPD—tumor burden increase ⩾20% compared with the lowest tumor burden.

Comparison between WHO criteria and the irRC

	WHO	irRC
New, measurable lesions (i.e., ⩾5 × 5 mm)	Always represent PD	Incorporated into tumor burden
New, nonmeasurable lesions (i.e., <5 × 5 mm)	Always represent PD	Do not define progression (but preclude irCR)
CR	Disappearance of all lesions in two consecutive observations not less than 4 wk apart	Disappearance of all lesions in two consecutive observations not less than 4 wk apart
PR	⩾50% decrease in SPD of all index lesions compared with baseline in two observations at least 4 wk apart, in absence of new lesions or unequivocal progression of non-index lesions	⩾50% decrease in tumor burden compared with baseline in two observations at least 4 wk apart

SD	50% decrease in SPD compared with baseline cannot be established nor 25% increase compared with nadir, in absence of new lesions or unequivocal progression of non-index lesions	50% decrease in tumor burden compared with baseline cannot be established nor 25% increase compared with nadir
PD	At least 25% increase in SPD compared with nadir and/or unequivocal progression of non-index lesions and/or appearance of new lesions (at any single time point)	At least 25% increase in tumor burden compared with nadir (at any single time point) in two consecutive observations at least 4 wk apart

Appendix6: Content of Informed Consent

Informed consent and patient information manual should include the following contents:

(a) The experiment involves research.

(b) Test purposes.

(c) The possibility of trial treatment and randomized allocation to each treatment.

(d) Test procedures to be followed, including all intrusive processes.

(e) Responsibility of patients.

(f) The characteristics of the experiment are experimental.

(g) Reasonable and predictable risks or inconveniences to patients and, if present, to embryos, fetuses or lactating babies.

(h) Reasonable expected benefits. When there is no specific clinical benefit for the patient, the patient should be made aware of this.

(i) Patients may be provided with alternative processes or courses of treatment, as well as significant potential benefits and risks.

(j) Provide compensation and/or treatment for patients with test-related injuries.

(k) Provide the patients who participated in the trial with anticipated pro rata payments, if any.

(l) Provide anticipated costs for patients participating in the trial if they occur.

(m) It is voluntary for patients to participate in the trial and patients can refuse to participate or withdraw from the trial at any time without penalty or loss of benefit.

(n) Inspectors, inspectors, IRB/EC and regulatory authorities (including overseas ones), in order to verify the clinical trial procedures and/or information, will authorize them to enter directly into the patient's original medical history records. To the extent permitted by applicable laws and regulations, records will be provided to regulatory authorities (including overseas regulatory authorities) without compromising patient confidentiality, and an informed contract will be signed. Letter of intent means that the patient or a legally recognized representative of the patient has authorized the entry of the person concerned.

(o) Recognizable patient records shall be kept confidential and shall not be made public to the extent permitted by applicable laws and regulations. If the test results are published, the patient's identity will be kept confidential.

(p) If access to information may be related to whether the patient voluntarily continues to participate in the trial, the patient or the legally recognized representative of the patient will be notified in real time.

(q) Contacts for further information on trial and trial patients' rights and interests, as well as contacts in the event of trial-related injuries.

(r) Predictable circumstances and/or reasons for the possible termination of the patient's participation in the trial.

(s) Expected time for patients to participate in the trial.

(t) The approximate number of patients involved in the trial.

(u) Reporting process of pregnancy, including spouse's pregnancy.

Appendix7: Statute of the Data and Safety Monitoring Board

The Data and Security Monitoring Board (DSMB) is the main data and security advisory group of bidders.

Regular meetings will be held to discuss the progress of the trial, in particular whether dose-limiting toxicity, dose escalation and approaching the maximum tolerable dose (be aware of the four-level cumulative toxicity occurring during the prolongation period).

DSMB regularly evaluates the safety results of trials, evaluates whether excessive adverse reactions occur in treatment, judges whether the overall safety of trials is acceptable, and makes recommendations for bidders, who are responsible for accepting, rejecting or modifying DSMB recommendations.

DSMB will specially assess adverse event data, as well as summary reports of all serious adverse events and laboratory data already available. Individual cases can also be assessed if deemed appropriate or necessary to determine whether safety problems occur. After evaluation, DSMB can recommend new action measures for specific treatment groups, or other appropriate measures to address common test safety issues that may arise. If deemed necessary, DSMB may at any time recommend that the entire test plan be suspended or permanently interrupted.

Composition of DSMB

Members include three clinicians (2 oncologists and 1 statistician), all of whom have past experience and expertise in routine clinical trials and have specialized DSMB experience. DSMB will receive materials directly from the data management center, which will be provided in a format requiring evaluation. Members of the committee may not participate in the trial as a principal or collaborating researcher or as a physician in charge of the subject.

Selection of DSMB Members

The chairman and members of DSMB will be chosen by the bidders. If members are unable to continue attending the DSMB meeting, the DSMB Chairman will recommend that the sponsor be replaced.

The members to be selected are:

Name list	Company	Title
Chengxu Cui	Cancer Hospital, Chinese Academy of Medical Sciences	Chief physician
Jifang Gong	Beijing Cancer Hospital	Deputy chief physician
Yanyan Song	Department of Biostatistics, Medical College of Shanghai Jiaotong University	Statistician

Duties and functions of DSMB

DSMB will be responsible for supervising test data related to safety considerations for bidders. At the beginning, DSMB will:

1. Establish the safety and related parameters to be monitored, the frequency of monitoring and evaluation by the committee, the evaluation methods and the criteria for providing recommendations to bidders.
2. DSMB reviews the data generated from the trial and regularly reviews safety incidents and recommends that the bidders take one of the following measures:

   (a) Interruption of trials (and rules for interruption in turn according to good medical norms).

   b) Modify the test plan. Modifications may include, but are not limited to, changes in selection/exclusion criteria, frequency of follow-up for safety monitoring, modification of test procedures, modification of observations, and duration of tracking.

   c) Continue to carry out tests in the light of the test plan and various relevant amendments.
3. DSMB will evaluate safety data after each patient in each population has completed a 21-day treatment cycle and make a decision on whether there is a dose-limiting toxicity.
4. Determine whether to increase the dose.
5. Determine when the maximum tolerable dose has been achieved.

Responsibilities of Bidders

The sponsor is responsible for DSMB for the following:

- Adequate resources are required for DSMB according to its designated functions.
- The patient's clinical data were prepared and sent to DSMB.
- Continue to be responsible for timely communication of all relevant regulatory information with individual researchers, the Institutional Review Committee (IRB) and the regulatory authorities of the State Food and Drug Administration (CFDA).

DSMB Meeting

Regularly scheduled meetings

Regular meetings will be held:

-Once all three patients in a population have completed an observation period to evaluate whether DLTs occur or the next population is about to begin;

-When MTD is expected to appear;

-If grade 4 toxicity occurs during the prolongation period.

If DSMB or bidders deem it necessary, further meetings will be held.

Quorum

At least three members of the committee are required at regular meetings, teleconferences or in order to vote on proposals for bidders.

Vote

DSMB votes on all proposals to be submitted to the bidders. For the purpose of

voting, members of the Committee must attend periodically scheduled meetings convened or attend by telephone. The participants voted by a simple majority to adopt proposals, motions or proposals submitted to the sponsor.

The process of making suggestions for bidders

DSMB proposals submitted to the sponsor for timely voting and adoption shall be submitted in writing to the sponsor within seven working days after the conclusion of the meeting at which the proposals were formed and adopted. If necessary, the sponsor is responsible for communicating the proposal with a separate researcher, IRB and CFDA.

Minutes of meeting

Under the supervision of the Chairman of the Committee, a detailed summary of each DSMB meeting is prepared. The minutes of the above meetings shall be sent to the sponsor in a timely manner. Since the trial treatment does not blind bidders, researchers and IRB, the minutes of the meeting will contain data disaggregated by the treatment population.

Form of conference

The meeting will consist of open and closed segments. During the initial opening part of the meeting, representatives of bidders, chairmen of DSMB trials or invited guests may be invited to make brief speeches and receive inquiries from members of DSMB at any time. Discussions on safety information and test progress will take place during the closed session, which will be attended by DSMB members and designated statisticians or data managers.

Secrecy

All members should keep their reports, discussions and minutes confidential.
